# Metatranscriptomic response of deep ocean microbial populations to infusions of oil and/or synthetic chemical dispersant

**DOI:** 10.1128/aem.01083-24

**Published:** 2024-07-23

**Authors:** Tito D. Peña-Montenegro, Sara Kleindienst, Andrew E. Allen, A. Murat Eren, John P. McCrow, Jonathan Arnold, Samantha B. Joye

**Affiliations:** 1 Department of Marine Sciences, University of Georgia, Athens, Georgia, USA; 2 Institute of Bioinformatics, University of Georgia, Athens, Georgia, USA; 3 Grupo de Investigación y Desarrollo en Ciencias, Tecnología e Innovación (BioGRID), Sociedad de Doctores e Investigadores de Colombia (SoPhIC), Bogotá, Colombia; 4 Microbial and Environmental Genomics, J. Craig Venter Institute, La Jolla, California, USA; 5 Integrative Oceanography Division, Scripps Institution of Oceanography, UC San Diego, La Jolla, California, USA; 6 Department of Medicine, University of Chicago, Chicago, Illinois, USA; 7 Josephine Bay Paul Center, Marine Biological Laboratory, Woods Hole, Massachusetts, USA; 8 Department of Genetics, University of Georgia, Athens, Georgia, USA; University of Delaware, Lewes, Delaware, USA

**Keywords:** *Colwellia*, *Marinobacter*, deepwater horizon oil spill, Corexit, metatranscriptome, mobilome, giant virus

## Abstract

Oil spills are a frequent perturbation to the marine environment that has rapid and significant impacts on the local microbiome. Previous studies have shown that exposure to synthetic dispersant alone did not enhance heterotrophic microbial activity or oxidation rates of specific hydrocarbon components but increased the abundance of some taxa (e.g., *Colwellia*). In contrast, exposure to oil, but not dispersants, increased the abundance of other taxa (e.g., *Marinobacter*) and stimulated hydrocarbon oxidation rates. Here, we advance these findings by interpreting metatranscriptomic data from this experiment to explore how and why specific components of the microbial community responded to distinct organic carbon exposure regimes. Dispersant alone was selected for a unique community and for dominant organisms that reflected treatment- and time-dependent responses. Dispersant amendment also led to diverging functional profiles among the different treatments. Similarly, oil alone was selected for a community that was distinct from treatments amended with dispersants. The presence of oil and dispersants with added nutrients led to substantial differences in microbial responses, likely suggesting increased fitness driven by the presence of additional inorganic nutrients. The oil-only additions led to a marked increase in the expression of phages, prophages, transposable elements, and plasmids (PPTEPs), suggesting that aspects of microbial community response to oil are driven by the “mobilome,” potentially through viral-associated regulation of metabolic pathways in ciliates and flagellates that would otherwise throttle the microbial community through grazing.

**IMPORTANCE:**

Microcosm experiments simulated the April 2010 Deepwater Horizon oil spill by applying oil and synthetic dispersants (Corexit EC9500A and EC9527A) to deep ocean water samples. The exposure regime revealed severe negative alterations in the treatments’ heterotrophic microbial activity and hydrocarbon oxidation rates. We expanded these findings by exploring metatranscriptomic signatures of the microbial communities during the chemical amendments in the microcosm experiments. Here we report how dominant organisms were uniquely associated with treatment- and time-dependent trajectories during the exposure regimes; nutrient availability was a significant factor in driving changes in metatranscriptomic responses. Remarkable signals associated with PPTEPs showed the potential role of mobilome and viral-associated survival responses. These insights underscore the time-dependent environmental perturbations of fragile marine environments under oil and anthropogenic stress.

## INTRODUCTION

Oil spills occur frequently in the marine environment, sometimes with calamitous consequences (1). Most marine oil spills have involved accidents in the coastal ocean (e.g., Exxon Valdez and MC20 Taylor Energy accidents) (2, 3), but offshore spillage of oil occurs as well, via operational discharges and drilling accidents, e.g., the Ixtoc oil well blowout in Mexico in 1979 (3) and the Deepwater Horizon (DWH) oil well blowout in the Gulf of Mexico in 2010 (1). The DWH oil disaster resulted from a massive explosion following the discharge of hydrocarbons, which continued for 84 days and released approximately 4.9 million barrels of oil and around 250,000 metric tons of natural gas into the Gulf of Mexico at a depth of 1,500 m. Such discharges provide acute, chronic, or pulsed inputs of oil into the marine environment that lead to modifications in the structure of marine microbial communities and can substantially alter microbial community function (1).

Given the magnitude of the DWH oil spill, synthetic chemical dispersants were used (4), both at the discharging wellhead and along surface oil slicks, as a response measure to move the oil from the organic phase (slick, discharge plume) to the aqueous phase to minimize environmental damage (e.g.*,* oiled beaches and marshes) and accelerating microbial oil biodegradation via the creation of small droplets with enhanced bioavailability (4). Synthetic chemical dispersants comprise petroleum distillates and non-ionic and anionic surfactants (5). These organic substrates are extremely labile, offering microbial populations an organic carbon source to fuel their metabolism (6, 7). Some 7 million liters of synthetic dispersant was applied in response to the DWH oil spill—2.9 million liters was applied at the discharging wellhead at the seabed—and while it is clear that synthetic dispersants altered microbial communities and potentially slowed oil degradation (8), exactly how dispersant-derived organic carbon modulated this response is unknown.

This work builds on the foundational work of Kleindienst et al. (9), which simulated the environmental conditions in the hydrocarbon-rich 1,100-m deepwater plume that formed during the DWH spill. In the Kleindienst et al. experiment (9), deep water (~200 L) from about 1,400-m water depth above an oil plume at a natural oil seep site (Green Canyon 600 in the Northern Gulf of Mexico) was collected on board a research vessel and returned to the laboratory for manipulation in a laboratory experiment. Several reports were generated from the Kleindienst et al. experiment (9), showing (i) changes in oil composition across treatments (10), (ii) patterns of the microbial production of transparent exopolysaccharides across treatments (11), and (iii) species-specific adaptations of *Marinobacter* and *Colwellia*, two key microbial responders to oil and dispersant infusions using metatranscriptomic data in the context of pangenomes (12). We used metatranscriptomic data collected from the Kleindienst et al. experiment to compare and contrast community-level expression signatures to reveal how microbial communities responded to distinct organic carbon exposure regimes of synthetic dispersants and/or oil.

The Kleindienst et al. experiment (9) involved exposing replicate (*n* = 3) microcosms to a matrix of controlled conditions to isolate the community response to oil versus dispersant exposure. Treatments were amended with a water-accommodated fraction (WAF) of oil-only, the synthetic dispersant (DISP) Corexit 9500, oil and Corexit mixtures (i.e., chemically enhanced water-accommodated fraction — CEWAF), and CEWAF plus nutrients (CEWAFN). The response of Gulf deepwater microbial populations to these additions was tracked over five time points in a span of 6 weeks (*t*_0_ = 0, *t*_1_ = 7, *t*_2_ = 17, *t*_3_ = 28, and *t*_4_ = 42 days), while replicate samples were sacrificed to assess microbial community composition, microbial activity, chemistry, and metatranscriptomics. Here, we advance the findings of Kleindienst et al. (9) by interpreting metatranscriptomic data from their experiment to explore how and why specific components of the microbial community responded to distinct exposure regimes.

## RESULTS

Transcriptomic libraries (*n* = 27) ranged in size from 4.6 to 18.75 million reads, with a mean of 10.58 million reads per sample and an average read length of 97 bp. About 44.38% of reads remained after quality control and removal of sequencing artifacts and duplicates (Fig. S1A). Predicted features were assigned to 68% of these reads, while 2.74% were associated with rRNA transcripts (Fig. S1B). In total, 11.3 million reads (30.18% of the initial reads) mapped taxonomic features with an average of 1,638 reads assigned to archaea, 403,781 reads assigned to bacteria, 5,357 reads assigned to eukaryotes, and 10,542 reads assigned to viruses (Fig. S2; Supplemental Data 1). Roughly 3.1 million reads per library were annotated at the functional level.

We observed significant differences in species diversity across treatments [one-way analysis of variance (ANOVA), *R*^2^ = 0.21, *P* < 0.0001]. Except for the biotic control and dispersant treatments, the samples showed differences of more than 1,000 species between the early stages of the experiment (*t*_0_ and *t*_1_) and the end of the experiment (*t*_4_), possibly driven to some degree by limitations in sequencing depth or inherent variability on diversity richness at a given sample (Fig. S3). For instance, using a sampling depth of 13.9 million reads as the cutoff, after 42 days of dispersant exposure, only ~4,095 species were recovered (i.e*.*, ɑ-diversity = 27); this was the lowest number compared to the WAF (~5,000 species), CEWAF (~6,000 species) and CEWAFN (~5,000 species) treatments (Fig. S3). Additionally, the ɑ-diversity index for the CEWAF ± nutrient treatments increased over time, while the rest of the treatments declined over time (Fig. S3F).

**Fig 1:**
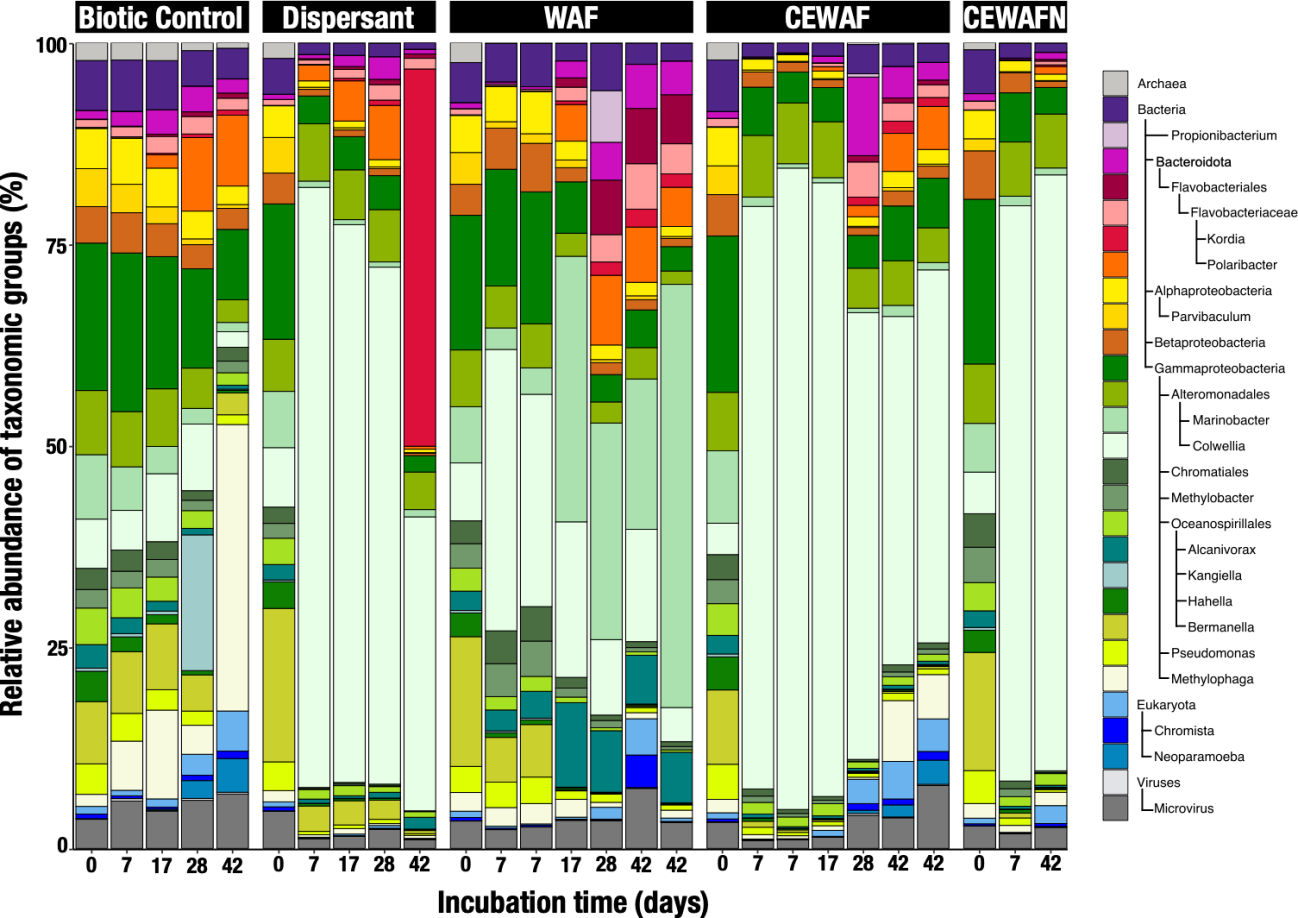
Relative abundance of merged taxonomic ranks in the Kleindienst et al. metatranscriptomic libraries at a minimum allowed resolution of 4% based on taxonomy assignment performed in MG-RAST.

Exposure to synthetic dispersants generated taxa-specific responses in expression that modulated the community response to different chemical combinations of oil and/or dispersant. The taxonomic profile of the active population identified by the annotated transcripts resembled the community structure shown through 16S rRNA gene sequencing (9). All dispersant-amended samples showed significant transcriptional enrichment for *Colwellia* (*t*-test, *P* < 0.0001)*,* an organism known for its role in hydrocarbon and dispersant degradation (13). After one week, the relative abundance of *Colwellia* transcripts increased from 3.9%–7.4% to 71.4%–79.6% in dispersant-only and CEWAF ± nutrient treatments (Fig. 1) and from 7.2% to 26.3%–34.9% in WAF treatments. *Colwellia* increased by 30.6% in gene expression compared to a 2.5% increase in abundance (16S rRNA gene counts) in the WAF treatment. *Marinobacter* accounted for a significant increase of transcriptional signals in WAF treatments compared to the rest of the treatments (*t*-test, *P* = 0.0206), with a relative increase from 7.0% to 18.7%–52.5% after 4 weeks (Fig. 1). In dispersant-only and CEWAF ± nutrient treatments, *Marinobacter* transcripts decreased from 6.0%–9.0% to 0.5%–0.8%. After the first week, the *Colwellia* transcriptomic response declined in WAF treatments, while that of *Marinobacter* increased. After 6 weeks of dispersant-only exposure, increased expression (up by 46.8%) by *Kordia* was observed; this relative increase was far more pronounced than the relative 16S rRNA gene counts of *Kordia* (up by 11.8%) (9). Although the difference in *Kordia* proportions (metatranscriptomic versus 16S signals) was not significant (*t*-test, *P* = 0.243), their trends over time showed significant differences (least square fit significant parameters, dispersant *P* = 0.0068, time *P* = 0.0134, dispersant × time *P*=0.0003).

**Fig 2:**
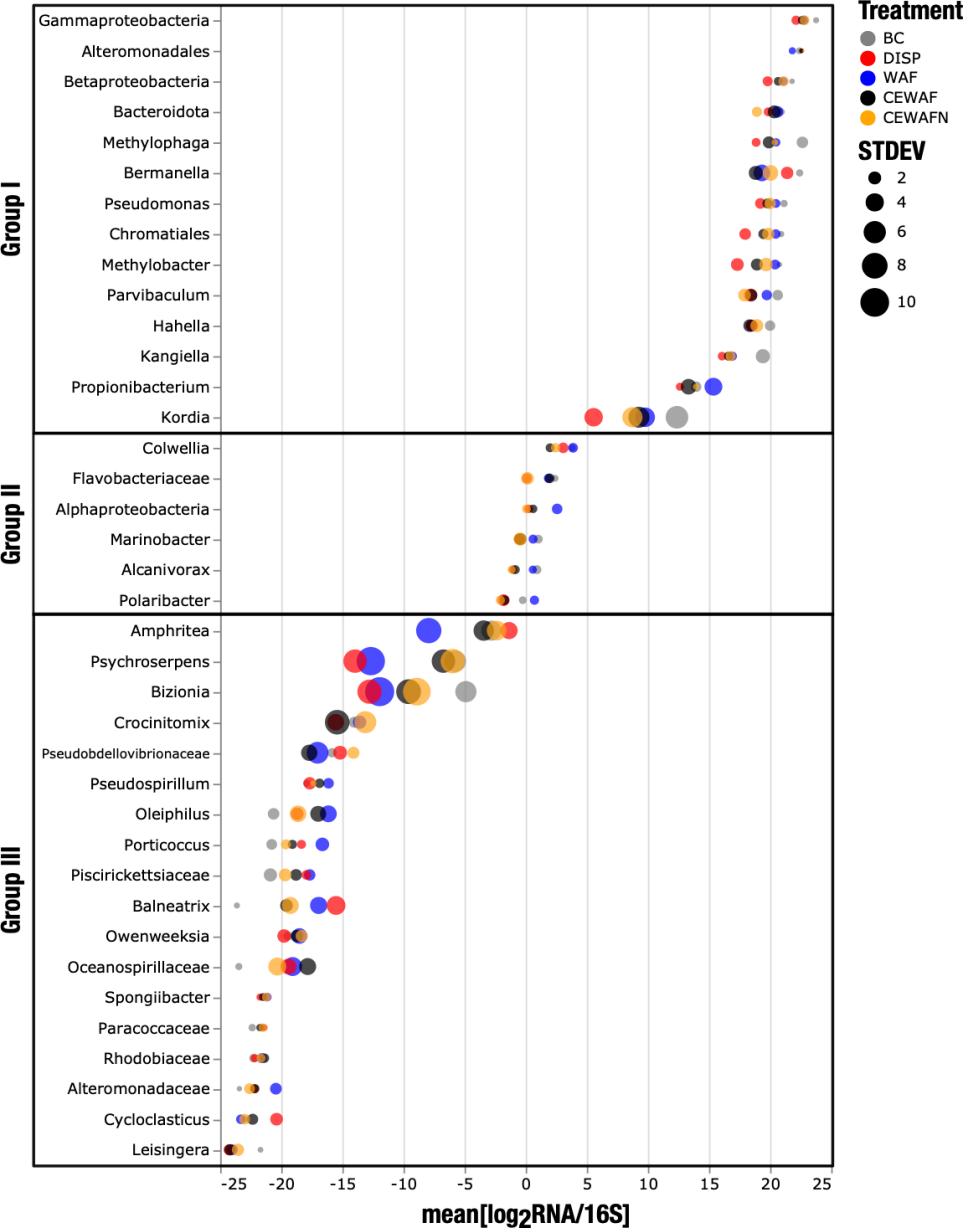
Indigenous hydrocarbon degraders are consistently found in 16S rRNA gene sequences as well as in transcriptomic libraries. LRD ratio distribution across Kleindienst et al. metatranscriptomic libraries. Taxonomic groups are sorted from top to bottom by descending mean of LRD scores. Biosynthetic capacity is expected to be the greatest in Group I (mean LRD >3.5), followed by Group II (|mean LRD| ≤3.5), and the smallest for Group III (mean LRD <−3.5). LRD, log-transformed RNA:DNA; BC, biotic control; DISP, dispersant; WAF, oil; CEWAF, dispersant + oil; CEWAFN, CEWAFN, CEWAF + nutrients; STDEV, standard deviation.

The ratio of total RNA to DNA has been proposed as an estimator for multiple physiological parameters (i.e., tissue development, growth fitness, and effective phenotype) (14). Here, we calculated the mean log-transformed RNA:DNA (LRD) ratio as an approach to describe the level of effective transcriptional activity (i.e*.,* synthetic capacity) between transcriptomic and 16S rRNA gene signals (9) across treatments. The largest fraction of relative counts at the transcriptomic and 16S rRNA gene level were distributed proportionately across treatments (Fig. 2, Group II: |mean LRD| ≤3.5), including hydrocarbon degraders such as *Marinobacter* and *Colwellia*. The group of organisms that had a larger relative synthetic capacity (Fig. 2, Group I: mean LRD >3.5) was composed of members associated typically with methylotrophic metabolism (*Methylophaga* and *Methylobacter*), oil influence (*Bermanella*), hydrocarbon degradation (*Pseudomonas*), and alkylbenzenesulfonate degradation (*Parvibaculum*) (15). Organisms with a low LRD index (Fig. 2, Group III: mean LRD <−3.5) included members of the family *Oceanospirillaceae*, such as *Amphritea*, *Pseudospirillum*, and *Balneatrix*; hydrocarbon degraders, including *Oleiphilus*, *Porticoccus*, and *Cycloclasticus*; as well as members of *Flavobacteria*, including *Bacteroidota*, *Pseudobdellovibrionaceae*, and *Spongibacter*. All LRD values presented in this analysis showed a significant correspondence across taxa (one-way ANOVA, *R*^2^ = 0.97, *P* < 0.0001).

**Fig 3:**
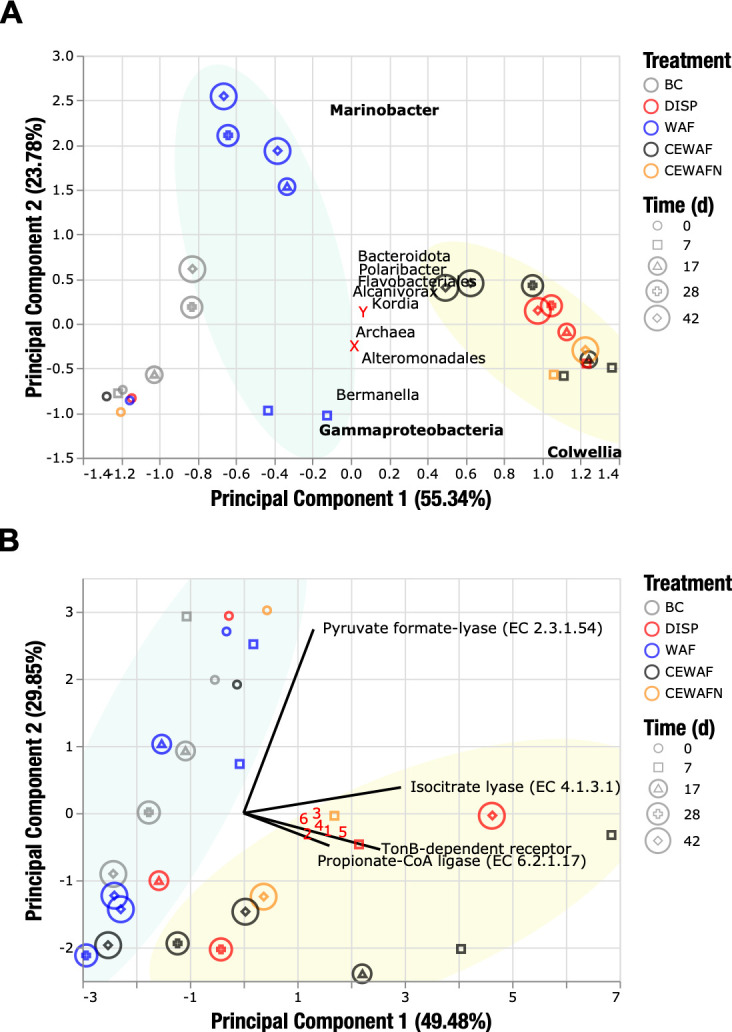
Diversity and functional dimensional analysis of the Kleindienst et al. metatranscriptomic data set. (A) Principal coordinate analysis of relative abundance of taxonomic groups. Near the X label, we found the following microbial groups: *Alphaproteobacteria*, *Betaproteobacteria*, *Oceanospirillales*, *Methylobacter*, *Parvibaculum*, Chromatiales, and *Hahella*. Near the Y label, we found the following microbial groups: *Neoparamoeba*, *Kangiella*, *Methylophaga*, *Chromista*, *Propionibacterium*, and *Microvirus*. (B) Functional expression of gene abundances assigned to the Subsystems for the Environmental and Ecological Data (SEED) subsystems: motility and chemotaxis, carbohydrates, membrane transport, and respiration. Solid lines represent the top 10 loading vectors explaining the expression signal variability in the analysis. Numbers in red are as follows: 1, 2-methylcitrate dehydratase FeS (EC 4.2.1.79); 2, acetoacetyl-coenzyme A reductase (EC 1.1.1.36); 3, aconitate hydratase 2 (EC 4.2.1.3); 4, acetolactase synthase large subunit (EC 2.2.1.6); 5, acetyl-coenzyme A synthetase (EC 6.2.1.1); and 6, malate synthase G (EC 2.3.3.9).

Beta-diversity was assessed via Bray-Curtis dissimilarity-based principal component analysis (PCA) of metatranscriptomic reads (Fig. 3A). Consistent with the taxonomic profile (Fig. 1), all treatments amended with dispersants were positively correlated with *Colwellia*. Oil-only samples occupied a separate cluster transitioning from a broad positive association with *Gammaproteobacteria* to a positive association with *Marinobacter* specifically. A third cluster comprising the biotic control spanned a positive association with *Gammaproteobacteria* with small positive contributions from *Marinobacter*, *Bacteroidota*, *Polaribacter*, *Flavobacteriales*, and *Alcanivorax*.

The interaction of oil ± dispersants and time was correlated significantly with changes in phylogenetic distances associated with the evolution of the microbial community. We performed a permutational multivariate analysis of variance on (i) taxonomic dissimilarity (i.e., Bray-Curtis) distances and (ii) phylogenetic dissimilarity via weighted mean pairwise distances (MPDs) and weighted mean nearest taxon distances (MNTDs). Dispersant (*R*^2^ = 0.27, *P* = 0.001) and time (*R*^2^ = 0.18, *P* = 0.001) terms significantly explained the variability of the Bray-Curtis distance profile. Similarly, dispersant (*R*^2^ = 0.09, *P* = 0.001), time (*R*^2^ = 0.06, *P* = 0.015), and dispersant × time (*R*^2^ = 0.05, *P* = 0.037) explained the MPD phylogenetic distance profile. In contrast, oil × time (*R*^*2*^ = –0.17, *P* = 0.010) and dispersant × oil × time (*R*^2^ = –0.59, *P* = 0.005) explained the MNTD phylogenetic distance profile, indicating that higher levels of dissimilar transcriptional responses were observed in dispersant treatments and over time. By weighting transcriptional abundances and phylogenetic proximity among taxa, we observed that the interaction terms oil × time and dispersant oil × time became significant for explaining MNTD dissimilarity distances in the data set. Additionally, we observed shifts of time-dependent trends of pathways in dispersant-only versus chemically enhanced water-accommodated oil fraction with or without nutrient treatments (Fig. S5 and S6).

**Fig 4:**
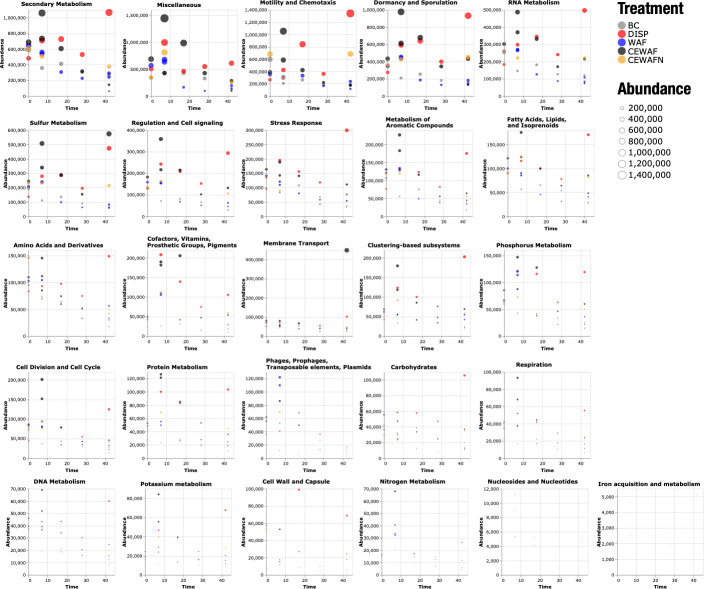
Abundance of gene expression based on automated SEED subsystems in MG-RAST for the Kleindienst et al. metatranscriptomic data set. Observations are color-coded by treatment. Dot sizes are proportional to the absolute abundance in a library. BC, biotic control; DISP, dispersant; WAF, oil; CEWAF, dispersant + oil; CEWAFN, CEWAFN, CEWAF + nutrients.

In the first 2 weeks after chemical exposure, the CEWAF treatment exhibited a relative expression increase in 20 functional categories (Fig. 4; Fig. S4), including “secondary metabolism,” “motility and chemotaxis,” “dormancy and sporulation,” “sulfur metabolism,” and “stress response” categories. These enriched categories ranged around ~300,000 to over 1 million mapping reads at peak expression across treatments (Fig. 4). In contrast, the dispersant treatment showed a delayed transcriptional response in the same categories. In 17 out of 20 categories, two behaviors occurred: (i) a fast, strong response near *t*_1_ and then a decay for the CEWAF treatment, and (ii) a slow incremental response with a maximum peak at *t*_4_ for the dispersant treatment. “Phages, prophages, transposable elements, and plasmids” were the only functional category where the oil-only treatment showed the largest relative expression peak across treatments. These results would suggest transcriptional responses acting simultaneously upon several metabolic functions of the community. In contrast, other functional categories showed different transcriptional profiles. For instance, phages, prophages, transposable elements and plasmids were the only functional group where the WAF treatment reached the largest relative abundance after 1 week of incubation. The “cell wall and capsule” category showed the largest relative abundance for the dispersant-only treatment at *t*_3_ and *t*_4_.

Here we also observed a strong signature expression of the *rebH* (DISP *P* value_adj_ = 0.001, WAF *P* value_adj_ = 0.001, CEWAF *P* value_adj_ = 0.001, and CEWAFN *P* value_adj_ = 0.003) and *prnA* (DISP *P* value_adj_ = 0.038, WAF *P* value_adj_ = 0.025, CEWAF *P* value_adj_ = 0.012, and CEWAFN *P* value_adj_ = 0.018) genes, coding for a flavin-dependent tryptophan halogenase (member of the secondary metabolism category) at the early stages of the experiment and with higher abundance for all dispersant amended treatments. All differentially expressed (DE) *rebH* and *prnA* transcripts were assigned to *Pelagibacter* phages, cyanobacteria-host phages, and *Colwellia* sp. MT41. The role of tryptophan halogenase, a precursor of the antibiotic pyrrolnitrin, suggests competition mechanisms for *Colwellia* and surrounding viral and phage-controlled microbial members (Supplemental Data 1).

To further assess the influence of the chemical exposure on the metabolic variation among the metatranscriptomes, a PCA of the functional feature abundance across the SEED annotation levels was conducted (Fig. 3B and 4). Dispersant-amended samples showed a clustering pattern separated from the WAF and biotic controls at the level of functions of major non-housekeeping modules (Fig. 3B). Biotic control and WAF libraries showed similar clustering trends along the pyruvate formate lyase (PFL) (EC 2.3.1.54) loading vector. Higher expression of PFL was strongly associated with the *t*_0_ libraries. On the other hand, CEWAF (*t*_1_ and *t*_2_) and dispersant (*t*_4_) samples showed a strong positive association with PC1, supported by the contribution of isocitrate lyase (EC 4.1.3.1), TonB-dependent receptor (TBDR), propionate-coenzyme A ligase (EC 6.2.1.17), and other features involved in the biosynthesis of amino acids; biosynthesis of storage compounds (i.e., polyhydroxybutanoate biosynthesis via acetoacetyl-coenzyme A reductase EC 1.1.1.36); generation of metabolite and energy precursors; and degradation of carboxylates, carbohydrates, and alcohols.

Dispersants and CEWAF treatments showed similar trends in contrast to the unique pattern in the oil-only treatment. This observation was consistent across SEED annotation levels and subsets of functional categories (Fig. 3B; Fig. S7). Over time, transcriptional profiles shifted toward a negative association with PC1 and PC2 for all except the dispersant-only treatments, potentially indicating systematic transcriptional changes associated with either the transition from an open-water system to a microcosm setting or the influence of unique perturbations of the dispersant-only treatment.

**Fig 5:**
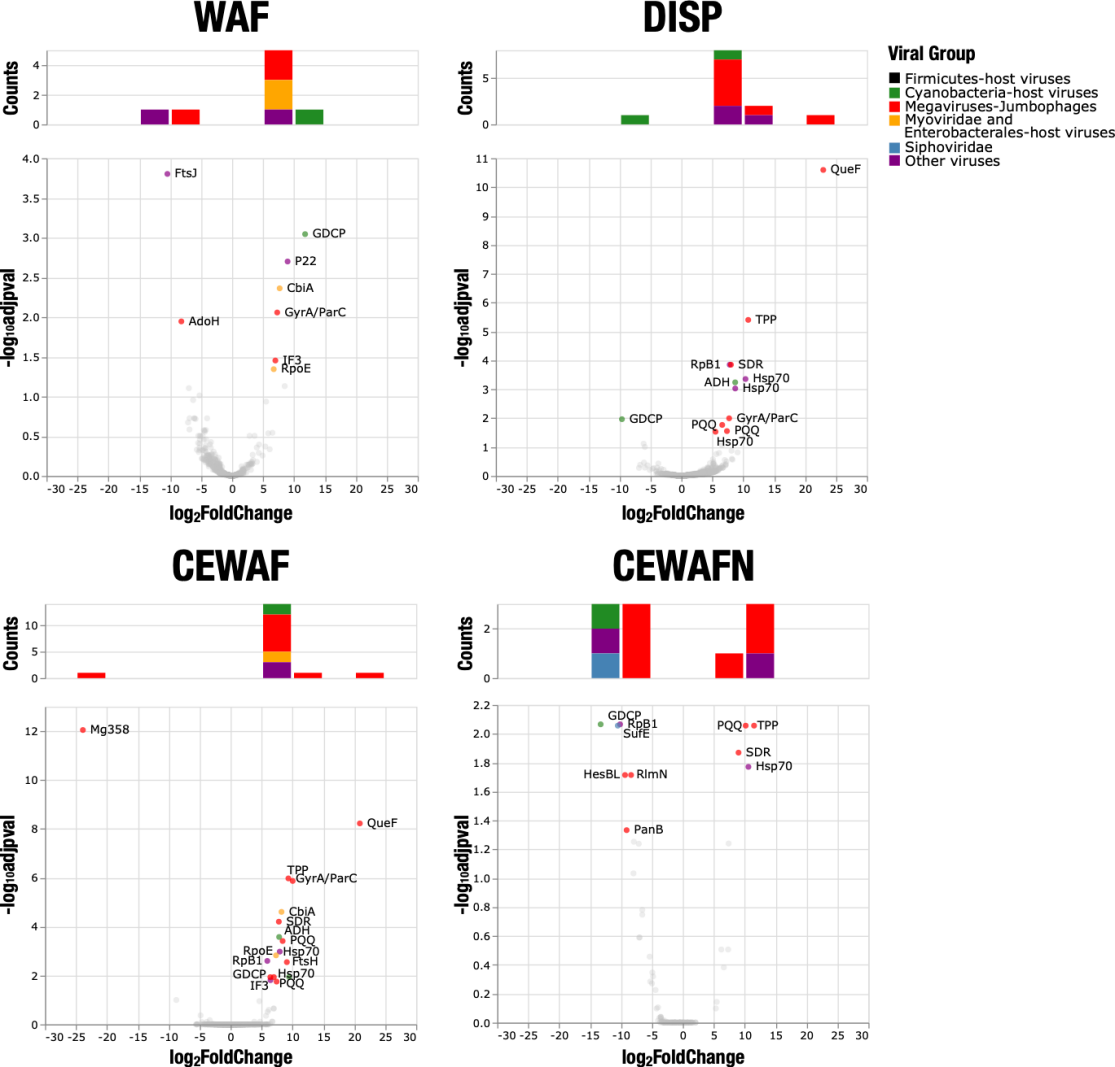
Volcano plots of genes assigned to viruses or prophages. Genes with *P* value_adj_ of <0.5 were considered as differentially expressed and are highlighted with their assigned functional and viral groups. Histograms on top of each volcano plot represent the distribution of DE genes by viral group and log2FoldChange. The two most common DE genes were Hsp70 (*n* = 6) and PQQ (*n* = 6), although several genes were frequently perturbed across treatments (e.g.*,* GDCP *n* = 4, SDR *n* = 3, TPP *n* = 3, and RpB1 *n* = 3).

Forty-seven DE genes associated with viruses or prophages were identified in the transcriptomic data set, ranging from 8 (WAF treatment) to 17 (CEWAFN) genes per treatment (Fig. 5; Supplemental Data 1). Over half of them were assigned to megaviruses or jumbo phages, while the rest covered cyanobacteria-host viruses (12.8%), *Chrysochromulina ericina* virus (17%), and other viral groups. Most viral DE genes were upregulated (78.7%).

The stress response chaperone protein Hsp70 was consistently upregulated in all the treatments (DISP *P* value_adj_ = 0.010, WAF *P* value_adj_ = 0.012, CEWAF *P* value_adj_ = 0.018, and CEWAFN *P* value_adj_ = 0.008) and was associated with a *Chrysochromulina ericina* virus or a *Bathycoccus* sp. RCC1105 virus BpV1 (Supplemental Data 1). Similarly, the quinoprotein ethanol dehydrogenase PQQ coding gene was upregulated in all the treatments and was associated with *Pandoravirus inopinatum* or *Pithovirus sibericum*, both classified as megaviruses (16). As expected, genes involved in translation, transcription, and replication, such as GyrA, RpB1, RpoE, Mg358, and IF3, dominated the upregulation of viral genes for the WAF, dispersant, and CEWAF treatments.

A few viral signatures were exclusively significant for certain species and treatments. An AdoH-AhcY ortholog, similar to SAH hydrolases of potent antiviral activity (17), was exclusively downregulated in the WAF treatment. This result could suggest that dispersants inhibited natural antiviral genes acting in place. Similarly, the major capsid gene P22 associated with the *Alteromonas* phage vB_AmaP_AD45-P1 was only upregulated in the WAF treatment.

## DISCUSSION

Detailed analyses of metatranscriptomic data from the Kleindienst et al. (9) microcosm experiment revealed interesting dynamics that were not apparent in the initial assessment. This new information helped elucidate the underlying response modes of the microbial population to differential exposure of oil, dispersant, and nutrients. Dispersant alone was selected for a unique community and for dominant organisms that reflected unique treatment- and time-dependent responses. Similarly, oil alone was selected for a community that was distinct from treatments amended with dispersants. In particular, dispersant- versus oil-amended treatments were selected for different dominant taxa, with *Colwellia* transcripts dominating dispersant treatments and *Marinobacter* transcripts dominating oil-only treatments, mirroring the results of Kleindienst et al. (9). The species-specific responses of these two dominant microorganisms were presented separately (12), but general aspects of their response are discussed here. Dispersant amendment also led to diverging functional profiles among the different treatments. The presence of oil and dispersants with added nutrients led to substantial differences in microbial responses, likely suggesting increased fitness driven by the presence of additional inorganic nutrients. Finally, the oil-only additions led to unanticipated increases in the expression of phages, prophages, transposable elements, and plasmids (PPTEPs), suggesting that aspects of microbial community response to oil is driven by the “mobilome” (18), mobile genetic elements in the microbiome.

### Dispersants altered expression of key microorganisms: *Colwellia* and *Marinobacter*

Comparison of results from 16S rRNA gene sequencing and metatranscriptomic libraries from the Kleindienst et al. (9) experiment provided insight into the response of microbial communities to an environmental perturbation, in this case, exposure to different cocktails of organic carbon that were derived from oil and/or dispersant. Based on 16S rDNA gene sequence data, two microorganisms*—Colwellia* and *Marinobacter*—dominated in different treatments: *Colwellia* was more abundant in dispersant-amended treatments, while *Marinobacter* was more abundant in oil-amended treatments. Metatranscriptomics data helped explain the success of these organisms in different experimental treatments (12).

Dispersants increased transcriptomic activity of *Colwellia* spp. (Fig. 1 and 3), in agreement with the original microcosm paper (9) that documented increases in *Colwellia* using 16S rDNA gene sequence data. Similar increases in abundance of *Colwellia* were observed in samples from the DWH ~1,000-m-deep hydrocarbon plume with oil (19), low-molecular-weight dissolved alkanes (20), a Corexit component (i.e., dioctyl sodium sulfosuccinate) (21), and other hydrocarbons with dispersant regimes (22, 23).

*Colwellia* is a psychrophile with substantial metabolic flexibility (24, 25). Genomic data illustrates a broad metabolic potential for *Colwellia* since its genome encodes the capacity for fermentation (propionate catabolism), beta-oxidation of fatty acids, glycolysis, the tricarboxylic acid cycle, and the pentose phosphate cycle (24). Previous studies have shown that *Colwellia* easily metabolizes glucose, acetate, and lactate (25). Furthermore, *Colwellia* may play a role in ethane and propane cycling (22). *Colwellia* produces polyhydroxyalkanoates as carbon and energy reserves, potentially accounting for the documented upregulation of these compounds in dispersant-amended treatments (Fig. 3B). *Colwellia* is particularly well-suited for polyhydroxyalkanoate production (26). The *Colwellia psychrerythraea* genome (24) contains a diverse suite of genes involved in polyhydroxyalkanoate metabolism, including several that lack homologs in other lineages. Though *Colwellia* spp. have not been shown to oxidize oil- or dispersant-derived organic compounds, genomic data suggest that these microbes could play important roles either in primary or secondary oxidation of oil- and dispersant-derived organic matter (24).

*Marinobacter* is one of the most common genera found in the global ocean and, as such, plays an important role in pelagic biogeochemical cycles (27). *Marinobacter* exploits environmental niches and survives, even thrives, under various conditions (27, 28). *Marinobacter* is known to oxidize saturated hydrocarbons (alkanes), cycloalkanes, and aromatics (toluene and polycyclic aromatic hydrocarbons) through coupling to aerobic or anaerobic (nitrate reduction) respiration. Unlike *Colwellia*, *Marinobacter* is not a psychrophile; quite the opposite, it is able to thrive in surface and deep waters, deep-sea sediments, and beach sands.

The addition of dissolved oil as a WAF without dispersants stimulated *Marinobacter*’s transcriptomic signals (Fig. 1 and 3). In contrast to *Colwellia*, dispersants limited the growth and replication of *Marinobacter* (9) and also muted their transcriptional activity. The stimulation of *Marinobacter’s* growth in oil-exposed microcosms, in the absence of dispersants, is consistent with reports of *Marinobacter* thriving in oil-rich marine snow flocs generated in the laboratory (29) and in pyrosequencing surveys of *in situ* seawater samples impacted by the DWH oil spill (30, 31). The transcriptomic data suggest that the reduction in *Marinobacter* abundance and activity (9) was not due to competition with *Colwellia* but rather a function of reduced activity, as evidenced by the reduction of transcriptional signal in dispersant treatments. This pattern suggests that dispersants inhibited the metabolic activity of the cosmopolitan oil degrader, *Marinobacter*, reducing its role in oil degradation in treatments amended with dispersants.

### Chemical exposure led to rapidly diverging functional profiles

Analyzing the metatranscriptomic data from the Kleindienst et al. (9) experiment also revealed the ecophysiological responses of all microbial groups. The LRD ratio assesses ecophysiological activity where a higher relative cell synthetic capacity, e.g., higher transcription read counts (RNA) relative to DNA, correlates with growth and nutritional status (32, 33). The underlying assumption of the LRD is that the amount of DNA in a system is stable under changing environmental conditions, while the amount of RNA is altered in response to changing conditions. Generally speaking, organisms thriving under a certain set of conditions will have a higher LRD ratio, while organisms poorly adapted to a given set of conditions will have a lower LRD ratio.

The LRD Group I, with a mean LRD of >3.5, likely contained highly active microorganisms given the extreme enrichment of transcription read counts over 16S rRNA gene read counts (Fig. 2). Methylotrophs (i.e., *Methylophaga* and *Methylobacter*) and native hydrocarbon degraders (i.e., *Bermanella* and *Parvibaculum*) were affiliated with this group (15, 34), consistent with previous reports of active indigenous oil degraders including members of these genera (35). *Methylophaga* was also stimulated by the by-products of high-molecular-weight dissolved organic matter metabolism in seawater, suggesting that they are important components of aerobic microbial associations that play key roles in organic carbon turnover (36).

To the best of our knowledge, this is the first report of transcriptional enrichment of *Kordia* in response to dispersants (Fig. 1). Previous studies reported ecological succession of bacterial clades with *Flavobacteriaceae* and *Paracoccaceae* in late August and September 2010 (37, 38). *Kordia*, a member of the *Flavobacteriaceae* family, was recently described at the pangenomic level (39), revealing that its core pangenome comprised a large fraction of cell wall and membrane biogenesis genes, peptidase, and TBDR encoding genes. At *t*_4_ in the dispersant-only treatment, *Kordia* increased transcriptional activity of TBDR, glyoxylate shunt, membrane biogenesis, and peptidase biosynthesis (Fig. 3B and 4; Fig. S4), matching previous descriptions of *Kordia* as an active player in niche colonization (39). Interestingly, the role of *Kordia* in the dispersant treatment was not apparent from the 16S rDNA gene sequence data. Clearly, the role of *Kordia* in dispersant degradation warrants further exploration.

LRD Group II contained taxa with the largest contribution to the microbial transcriptomic profile, underscoring its effective and rapid response to the different chemical cocktails. This group included indigenous hydrocarbon degraders *Oceanospirillales*, *Colwellia*, and *Marinobacter* (Fig. 2), as well as several other known oil degraders, e.g., *Alcanivorax*, a slow-growing *n*-alkane degrading member of the *Gammaproteobacteria* (40), and *Polaribacter*, a member of the Flavobacteria, known to degrade polysaccharides (41) as well as hydrocarbons (7). The LRD Group II also included various members of the *Flavobacteriaceae*, the largest family in the phylum *Bacteroidota*, with members capable of aerobic and microaerophilic degradations of polysaccharides and members of the *Alphaproteobacteria*, a class of bacteria in the phylum *Pseudomonadota*.

Members of LRD Group III were poorly adapted for the experimental conditions relative to members of LRD Group I and LRD Group II. LRD Group III included members of the family *Oceanospirillaceae*, such as *Amphritea*, *Pseudospirillum*, and *Balneatrix*, some hydrocarbon degraders, including *Oleiphilus*, *Porticoccus*, *Cycloclasticus*, *Paracoccaceae*, *Rhodobiaceae*, and *Alteromonadaceae*, and members of *Flavobacteria*, *Bacteroidota*, *Pseudobdellovibrionaceae*, and *Spongibacter* (Fig. 2). Many hydrocarbon degraders are slow growing in nature, and membership in LRD Group III could reflect time-sensitive, adaptive responses that occur across different groups of hydrocarbon-degrading bacteria over longer time scales. This pattern may also reflect specific niche-adaptation strategies in hydrocarbon-degrading microbial communities.

### From metabolic variations across treatments to viral-phage DE genes

The strongest transcriptomic signals were observed in the “secondary metabolism,” “miscellaneous,” “motility and chemotaxis,” “dormancy and sporulation,” and “RNA metabolism” categories (Fig. 4). The expression trends observed over time usually showed either an expression peak near *t*_1_ for the CEWAF treatment or toward *t*_4_ for the dispersant treatment. The unique trajectories observed in the expression profiles across treatments suggest strongly that different cocktails of complex organic carbon led to distinct time-dependent responses of the microbial populations. These results are consistent with significant shifts observed in the differentially expressed meta (DEM)-pangenome of *Colwellia* and *Marinobacter* (12), as well as the clustering transitions of taxonomic and functional profiles in the microbial community (Fig. 3)

Pyrrolnitrin is a secondary metabolite derived from tryptophan and was first isolated from *Pseudomonas pyrrocinia*. It is described as a broad-spectrum antifungal and antibacterial agent with application in the control of soilborne pathogens and postharvest diseases, including *Rhizoctonia solani* and *Botrytis cinerea* (42). Pyrrolnitrin and other halometabolites have been isolated from marine microorganisms, including pentabromopseudilin, found in *Pseudomonas bromoutilis* (43), and bromopyrroles, found in *Pseudoaltermonas* spp. (44). Although pyrrolnitrin has been widely used to treat human infections, including the fungicide fludioxonil (45), its biosynthesis pathway is still not fully understood. Finding the association of the expression of RebH and PrnA coding sequences with *Pelagibacter* phages, cyanobacteria-host phages, and *Colwellia* sp. MT41 suggests a new mechanism that potentially improves the ecological fitness of *Colwellia* and phage-controlled microbes to dispersant-polluted environments. We hypothesize two possible explanations:

1. The biosynthesis of intermediates with bacteriocide activity. The reaction kinetics of flavin-dependent tryptophan halogenase requires first the completion of flavin redox reactions followed by substrate chlorination (46, 47), leading to the formation of harmful reactive species such as hypochlorite and peroxide.
2. A still unknown flavin-dependent tryptophan halogenase may be participating in the redox modulation of virus-host receptor interactions. Pathway coordination aiming for redox balance has been associated with the successful reactivation of viral infectivity through stable receptor recognition. This phenomenon has been described previously, including virus-host receptor interactions of human diseases such as severe acute respiratory syndrome coronavirus 2 and HIV (48, 49).

Both hypotheses remain open to further investigation.

To inspect expression changes in PPTEPs, we selected gene counts associated with phages or viruses after performing DE analysis to the complete data set. The megaviruses and jumbo phages were the only functional group with the highest relative peak in the oil-only treatment (Fig. S4). Further inspection of DE genes assigned to viral groups revealed that most viral DE genes were upregulated in all of the amended treatments, while downregulation was observed only in the CEWAFN treatment (Fig. 5).

Megaviruses are important, yet poorly understood, components of ocean ecosystems (16, 18, 50, 51). Megaviruses can infect the ciliate and flagellate grazers that impart top-down control on microbial populations (52, 53). As such, an intriguing possibility is that the ingrowth of megaviruses in response to oil exposure could relax predation and allow populations of oil degraders to bloom in the absence of predators. Megaviruses may serve a key role in the case of oil spills and the microbial response to them, i.e., the presence of megaviruses may promote the success of oil-degrading microbial communities directly (54). Genomic comparisons of megaviruses isolated in coastal waters revealed the paradox of megaviruses showing convergent evolution, while the number of unique protein-coding genes overwhelmingly outnumbered their core genes (55). Hence, this is an open opportunity to identify the role of the adaptive pangenome of megaviruses through the lens of a metatranscriptomic data set. Additional work is under way to create a megavirus DEM-pangenome following the procedure in Peña-Montenegro et al. (12, 56).

The viral repertoire was different in the CEWAF versus the CEWAFN treatment. The inherent stress imposed on the microbial community through nutrient limitation appears to have led to a more substantial viral-induced stress response in the CEWAF treatment compared to the CEWAFN treatment (Fig. 5). These results could be associated with changes in the microbial activity and the formation of marine oil snow patterns reported by Kleindienst et al. (9), as well as perturbations at the expression level, including lipid metabolism and hydrocarbon degradation genes, in *Colwellia* (12). Such positive stimulation for degradation and stress response activities under nutrient amendment could be indicative of an underlying nutrient-limited system, as suggested elsewhere (57). Previous studies showed how environmental conditions impacted the relationship between viruses and bacteria using multivariate models demonstrating that environmental factors, such as inorganic nutrient concentrations, are important predictors of host and viral abundance across a broad range of temporal and spatial scales (58). Similarly, trophic interactions in the microbial food web, such as predation and the availability of limiting nutrients, were shown to affect the structure and function of viral and prokaryote communities (59). We suspect that the absence of nutrients, i.e., nutrient limitation, could have triggered stress responses in the microbial communities, with a collateral effect on the activation of viral communities.

### Conclusions

Comparison of results from 16S rRNA gene sequencing and metatranscriptomic libraries from the Kleindienst et al. (9) experiment provided insight into the response of microbial communities to an environmental perturbation, in this case, exposure to differential cocktails of organic carbon that were derived from oil and/or dispersant. The presence of dispersant increased transcriptomic activity of *Colwellia* spp., and *Colwellia* may have been involved in the degradation of oil or dispersant components in the microcosms, as well as in field samples (22, 23). In contrast, dispersants limited the growth and replication of *Marinobacter* (9) and also muted their transcriptional activity, reducing its role in oil degradation in treatments amended with dispersants. The LRD ratio proved to be a valid tool to assess microbial groups’ relative ecophysiological activity levels across treatments and in response to nutritional status, showing that some oil-degrading microorganisms are more adept at responding to oil or oil plus dispersant exposure than others. Transcriptional data revealed a potentially important role for *Kordia* in the metabolism of dispersant-derived organic matter; the importance of these organisms was not clear from 16S rRNA gene composition data. The upregulation of genes from megaviruses, especially in oil-only treatments, suggests an important role for these organisms in shaping the oil-spill-perturbed microbiome. Accelerated activity of megaviruses could relieve grazing pressure on oil-degrading microorganisms, hence promoting their rapid growth in response to oil infusions. The presence of dispersants may inhibit this, which could explain why different microbial populations thrive under conditions of oil versus dispersant exposure.

## MATERIALS AND METHODS

### Microcosm setup

Methods in this section were described in detail and performed by Kleindienst et al. (9). In brief, deep water was collected at 1,178-m depth in the northern Gulf of Mexico (latitude 27.3614, longitude −90.6018). Water was stored in 20-L carboys and transported to the University of Georgia laboratory. Seventy-two glass bottles (1.8-L sample per bottle) were incubated on a roller table. Each treatment aimed to simulate the DWH deepwater plume. Treatments resulted from the mixture of pasteurized seawater with WAF of oil-only (DWH plume *in situ* dissolved organic carbon ~150 µM), synthetic DISP Corexit 9500 (~19 µg/L), and CEWAF (WAF + Corexit 9500 ~ 19 µg/L). CEWAFN treatment was amended with 10-µM ammonium chloride, 10-µM potassium nitrate, 1-µM potassium phosphate, final concentrations, and trace metals. Sampling was performed over five time points in a span of 6 weeks (*t*_0_ = 0 day, *t*_1_ = 7 days, *t*_2_ = 17 days, *t*_3_ = 28 days, and *t*_4_ = 42 days), except for the CEWAFN treatment only, including *t*_0_, *t*_1_, and *t*_4_. The biotic control was not amended.

### Sampling and sequencing

Filters were frozen in liquid nitrogen, kept on dry ice for shipping, and stored in the laboratory at −80°C. RNA was purified from filters using the Trizol reagent (Life Technologies, Carlsbad, CA, USA) and treated with DNase (Qiagen, Valencia, CA, USA). RNA quality was analyzed using a 2100 Bioanalyzer with Agilent RNA 6000 Nano Kits (Agilent Technologies, Santa Clara, CA, USA) and quantified using the Qubit Fluorometric Quantification system (Thermo Fisher, Waltham, MA, USA). Total RNA (10–100 ng) was subjected to amplification and cDNA synthesis using the Ovation RNA-Seq System (version 2) (NuGEN). One microgram of the resulting high-quality cDNA pool was fragmented to a mean length of 200 bp, and Truseq (Illumina) libraries were prepared and subjected to paired-end sequencing via Illumina HiSeq.

### Data workflow

Paired-end libraries were imported into FASTQC (http://www.bioinformatics.bbsrc.ac.uk/projects/fastqc) to scan for sequencing quality. Adapter removal, read, and quality trimming were completed in Trimmomatic (version 0.36) (60), where a 4-bp sliding window was applied to retain bases with quality scores greater than 20. Trimmed paired-end reads were assembled with Trinity (version r20140717) (61) to generate *de novo* reference transcriptomes (mean contig length: 460 bp, mean N50: 487 bp, mean total: 34 million bp) for each of the samples. We used Prodigal (version 2.6.3) (62) with default settings to identify open reading frames. Reference assembly annotations were determined by BLASTX (version 2.2.31) query searches against the National Center for Biotechnology Information (NCBI) non-redundant protein database, SwissProt, and TrEMBL (63, 64). Gene ontology annotations were determined by mapping SwissProt/TrEMBL IDs to the UniProt-GOA database (65). Viral contigs were confirmed with MetaPhinder (66). To compensate for potential data processing biases of our selected annotation procedure, we included an automatic annotation strategy as follows. Paired-end reads for each library were merged and imported into MG-RAST (version 4.0.3) (67). Taxonomic and functional hits were queried against the MG-RAST Subsystems database (≤1e−4, ≥33% identity, minimum alignment length 15 aa). Annotation calls from BLASTX and MG-RAST were integrated by using the totalannotation.py script found in the De Wit et al. pipeline (68).

Given the complex and variable taxonomic labeling systems across the annotation sources, we developed an R script to (i) translate from variable taxonomic labels into a unified and restricted vocabulary space and (ii) aggregate a large list of taxonomic labels into a smaller list by combining taxonomic calls of low relative abundance into higher-rank taxonomic groups until reaching a minimum relative abundance threshold. This tool is detailed in our dedicated GitHub module (https://github.com/biotemon/TaxonomyModule). The input for the script was an array with observations in tuple format (*sample_id*, *query*, and *counts*). Then, the script generated a matrix representation of the query label in terms of the SQLite reference table. If relative *counts* (*r*) for a given *query* representation (*q*) were below a minimum threshold (*T*) (e.g., 4%), the script merged *r* to the next higher taxonomic rank until *r* ≥ *T*.

### Differential expression analysis

We aligned all libraries to assembled transcriptomes using Bowtie2 (69). To contrast gene expression with respect to the biotic control, we used HTSeq-count to generate the read counts of genes recruited by the reference genomes (70). Profiles were normalized across all samples using the regularized logarithm transformation as implemented in DESeq2 using a generalized linear model (71). Then, statistical inference was performed using the negative binomial Wald test with Cook’s distance to control for outliers (72). Those genes with an adjusted *P* value of <0.05 (using the Benjamini-Hochberg method) were classified as DE genes (73).

### LRD and diversity analysis

To calculate LRD ratios, we used operational taxonomic unit (OTU) abundance counts obtained from the Kleindienst et al. data set (9). Raw 16S rRNA gene amplicon sequences are available under the NCBI Bioproject PRJNA253405. Relative OTU abundance triplicates were averaged ($\underline{d}$) and subjected to a higher-rank taxonomic merging with a minimum relative abundance threshold of 4%. LRD index is defined as


$$LRD_{ij}=\frac{1}{n}\sum_{t=1}^{n}\log_{2}\frac{r_{ijt}}{d_{ijt}}$$

where the log-fold ratio between metatranscriptomic (*r*) and 16S rDNA gene ($\underline{d}$) relative counts are averaged across time (*t*) for each taxonomic group (*i*) and treatment (*j*).

Rarefaction and alpha-diversity analyses of metatranscriptomic data sets were performed through MG-RAST. To evaluate beta-dispersion at the expression level of the microbial communities between all treatments, beta-phylogenetic differences weighted by abundance were tested using comdist (the average MPD for each species in a sample to all species in another sample) and comdistnt (the average MNTD for all species in a sample to the nearest neighbors in another sample) using the package Picante (74). A sequence-based reference phylogenetic tree was used as input parameter for the MPD and MNTD distance matrix calculations. To build the reference phylogenetic tree, we followed procedures described previously (75). In brief, taxonomic representatives shown in Fig. 1 were selected for searches of archaeal, bacterial, and eukaryotic ribosomal small subunit sequences as well as viral capside and coat protein coding sequences. A full description of taxonomic representative sequences is available in Supplemental Data 2. Sequences were aligned in MAFFT. Maximum likelihood phylogenetic trees of aligned sequences were inferred with RAxML using the general time-reversible model for substitution and the GAMMA model for rate heterogeneity. Tree topologies were checked by 100 bootstrapping replicates.

Bray-Curtis dissimilarity among all treatments was calculated as an abundance-weighted measure of beta-diversity. The output distance matrix was visualized via PCA ordinations using the pcoa function from the ape package. The function adonis from the vegan package was used to examine the significant relationship between distance matrices (*U*) (i.e., Bray-Curtis dissimilarity, MPD, or MNTD) and experimental factors: dispersant (*D*), WAF (*O*), nutrients (*N*), and time (*t*). Each test comprised 999 permutations. The testing model was defined as follows:


U=O+D+O×D+O×D×N+t+O×t+D×t+O×D×t+O×D×N×t

## Data Availability

Raw sequencing reads generated for this study can be found in the Sequence Read Archive under BioProject PRJNA640753. MG-RAST annotation output is publicly available at https://www.mg-rast.org/linkin.cgi?project=mgp84528. All scripts are found on GitHub (https://github.com/biotemon/K2015 and https://github.com/biotemon/TaxonomyModule). Taxonomy rank database and all other data are available in the Open Science Framework repository at https://osf.io/fu9bw/.

## AFTER EPUB

*[This article was published on 23 July 2024 with a statement missing from Acknowledgments. The Acknowledgments were corrected in the current version, posted on 26 July 2024.]*

## SUPPLEMENTAL MATERIAL

SuppCode_1 aem.01083-24-s0001.pdf Supplemental code part 1.

SuppCode_2 aem.01083-24-s0002.pdf Supplemental code part 2.

SuppData1 aem.01083-24-s0003.xlsx Differential expression analysis and annotation of predicted gene sequences in the Kleindienst et al. metatranscriptome.

SuppData2 aem.01083-24-s0004.xlsx Representative sequences to build a guiding phylogenetic tree for phylobetadiversity analysis of the metatranscriptomic dataset.

Supplemental figures aem.01083-24-s0005.pdf Figures S1 to S7.

ASM does not own the copyrights to Supplemental Material that may be linked to, or accessed through, an article. The authors have granted ASM a non-exclusive, world-wide license to publish the Supplemental Material files. Please contact the corresponding author directly for reuse.
