# Supplementary material for: Metatranscriptomic response of deep ocean microbial populations to infusions of oil and/or synthetic chemical dispersant
Source: Appl Environ Microbiol. 2024 Jul 23;90(8):e01083-24. doi: 10.1128/aem.01083-24 (PMC11337851; doi:10.1128/aem.01083-24)
Supplement: SuppCode_1 — Supplemental code part 1. [file aem.01083-24-s0001.pdf]

Supporting code used for:

Metatranscriptomic response of deep ocean microbial populations to infusions of oil and/or synthetic chemical dispersant
– in submission to Applied and Environmental Microbiology

Click here for the code of:

- Figure 1
- Figure 2
- Figure 3A
- Figure 3B
- Figure 4
- Supplementary Figure 1
- Supplementary Figure 2
- Supplementary Figure 3
- Supplementary Figure 4
- Supplementary Figure 5 and 6
- Supplementary Figure 7

These results were also referenced in the working paper:

Peña-Montenegro et al. Colwellia and Marinobacter metapangenomes reveal species-specific responses to oil and dispersant exposure in deepsea microbial communities https://doi.org/10.1101/2020.09.28.317438

```
In [1]:

# Import libraries
import altair as alt
from altair import datum
from IPython.display import SVG, display
import math
import matplotlib.pyplot as plt import numpy as np
import os
import pandas as pd import re
from scipy.optimize import curve_fit
from sklearn.decomposition import PCA
from sklearn.preprocessing import StandardScaler
from sklearn.metrics import r2_score
import statsmodels.api as sm
from statsmodels.stats.outliers_influence import summary_table
%matplotlib inline
alt.renderers.enable('default')
```

Out[1]: RendererRegistry.enable('default')

Supplementary Figure 1

In [2]:

```
#Loading data

Location = './sk_summary_data.csv' sk_df = pd.read_csv(Location)


#Subset columns and melting table into long format
qc_df=sk_df.loc[:,['sample_id','passed_qc','failed_qc']] qc_df_longformat
= pd.melt(qc_df, id_vars=['sample_id'], var_name='Quality Check',
value_name='Reads')

#Generating Plot

figS1A = alt.Chart(qc_df_longformat).mark_bar().encode( alt.Color('Quality
Check', legend=alt.Legend(title='Quality Check'),
scale=alt.Scale(domain=['passed_qc', 'failed_qc'], range=['#EFCC00',
'#c7c7c7']),),alt.X('sample_id:N',
axis=alt.Axis(title='sample_id'),),y='Reads:Q',)

figS1A
```

Out[3]:

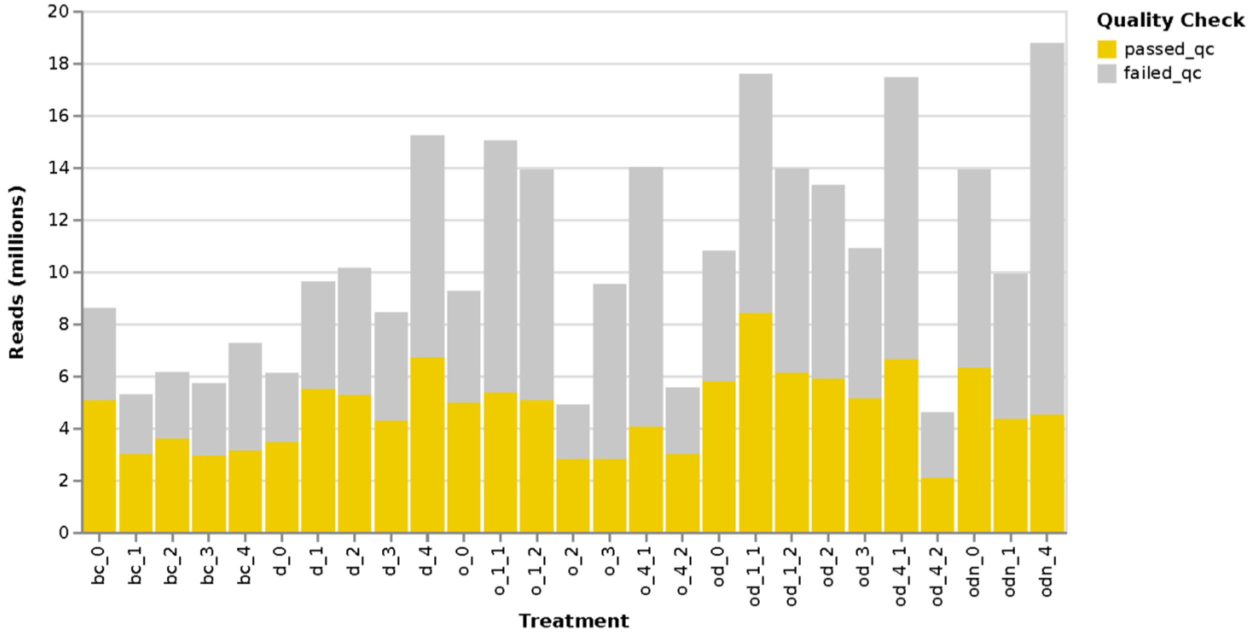

In [4]:

```
#Subset columns

rrna_df=sk_df.loc[:,['sample_id','rna_reads','pred_prot_reads','unknown_
fun_reads']]
rrna_df.columns=['Treatment','B','C','A']

#melting table into long format

rna_df_longformat = pd.melt(rrna_df, id_vars=['Treatment'], var_name='Qu
ality Check', value_name='Reads')
rrna_df_longformat = pd.melt(rrna_df, id_vars=['Treatment'], var_name='A
nnotation', value_name='Reads')

#Generating Plot

figS1B = alt.Chart(rrna_df_longformat).mark_bar().encode(
alt.Color('Annotation:N',
legend=alt.Legend(title='Predicted Features'),
scale=alt.Scale(domain=['A', 'B', 'C'], range=['#c7c7c7', '#EFCC00',
'#1f77b4']),),alt.X('Treatment:N',
axis=alt.Axis(title='Treatment'),),y='Reads', )

#Where A= Unknown Function B= rRNA genes C= Predicted
#We used ABC notation to force the order of the stacked bar

figS1B
```

Out[4]:

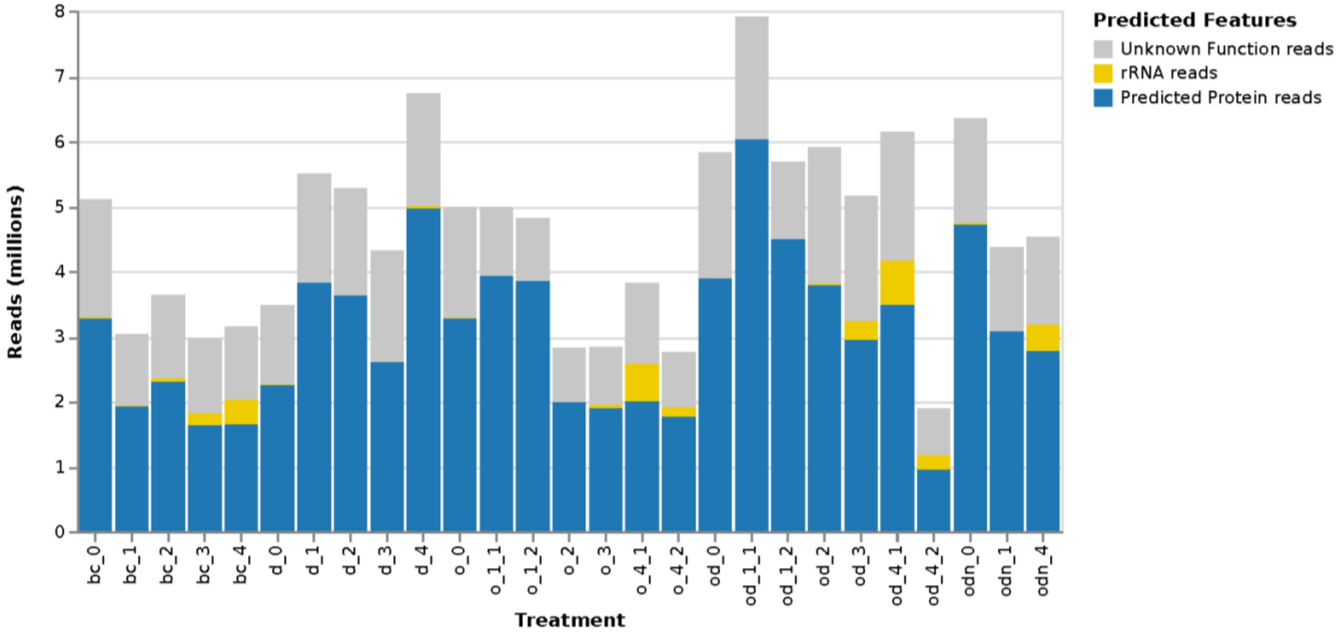

Supplementary Figure 2

In [5]:

```
#Subset columns
cog_df=sk_df.loc[:,['sample_id','cog_cell_proc', 'cog_info_storage',
'cog_metabolism', 'cog_poorly']]
plot_df = cog_df plot_df.columns=['Treatment','B','C','D','A']

#Normalizing table by column sums.
plot_df_2 = plot_df.drop('Treatment',axis=1)
vec = plot_df_2.sum(axis=1)
plot_df_2 = plot_df_2.div(plot_df_2.sum(axis=1),axis=0).mul(100)
plot_rel_df = plot_df['Treatment'].to_frame().join(plot_df_2)
the_leg = 'COG Annotation'
the_leg_t = 'COG Annotation'+':N'
the_y = 'Reads'

#melting table into long format
plot_df_longformat = pd.melt(plot_rel_df, id_vars=['Treatment'], var_nam
e=the_leg, value_name=the_y)

#Generating Plot
figS3A = alt.Chart(plot_df_longformat).mark_bar().encode(
alt.Color(the_leg_t,
legend=alt.Legend(title=the_leg), scale=alt.Scale(domain=['A', 'B', 'C',
'D'], range=['#c7c7c7', '#EFCC00', '#1f77b4', '#9467bd']),),
alt.X('Treatment:N', axis=alt.Axis(title='Treatment'),),y=the_y, )

figS3A
```

Out[5]:

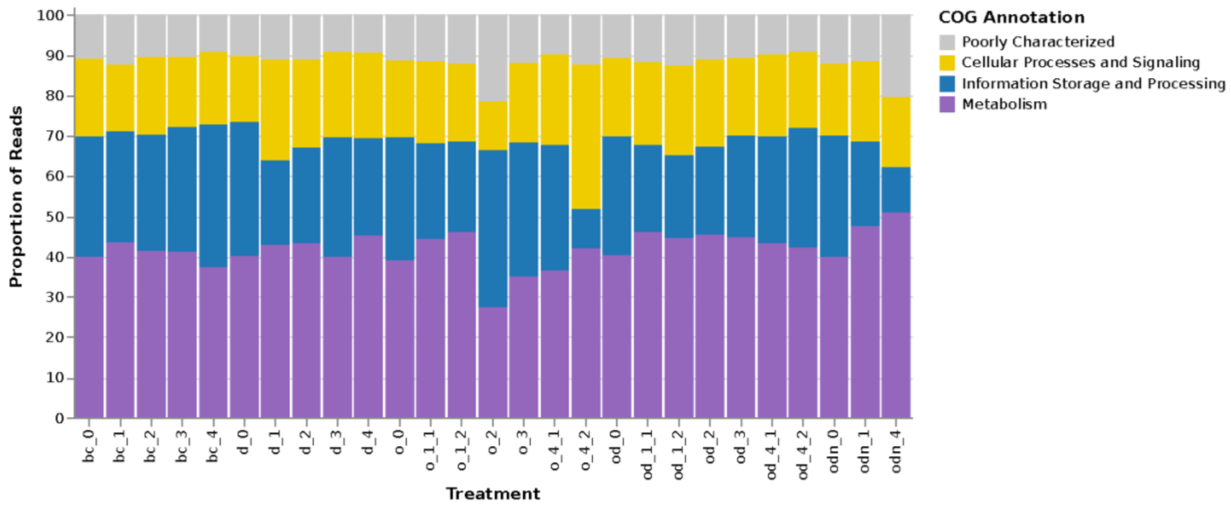

In [6]:

```
#Subset columns for figure KO_df=sk_df.loc[:,['sample_id','ko_cell_proc',
'ko_env_info_proc', 'ko_g ene_info_proc', 'ko_human_diseases',
'ko_metabolism', 'ko_organismal_sy s']]

plot_df = KO_df plot_df.columns=['Treatment','B','C','F','A','E','D']

#Normalizing table by column sums.
plot_df_2 = plot_df.drop('Treatment',axis=1)
vec = plot_df_2.sum(axis=1)
plot_df_2 = plot_df_2.div(plot_df_2.sum(axis=1),axis=0).mul(100)
plot_rel_df = plot_df['Treatment'].to_frame().join(plot_df_2)
the_leg = 'KO Annotation'
the_leg_t = 'KO Annotation'+':N'
the_y = 'Reads'
#melting table into long format
plot_df_longformat = pd.melt(plot_rel_df, id_vars=['Treatment'], var_nam
e=the_leg, value_name=the_y)

#Generating Plot
figS3B = alt.Chart(plot_df_longformat).mark_bar().encode(
alt.Color(the_leg_t, legend=alt.Legend(title=the_leg), scale=alt.Scale(
domain=['A', 'B', 'C', 'D', 'E', 'F'], range=['#000000', '#EFCC00',
'#c7c7c7', '#1f77b4', '#9467bd', '#EE7F2D']
), ), alt.X('Treatment:N', axis=alt.Axis(title='Treatment'),
), y=the_y, )


figS3B
```

Out[6]:

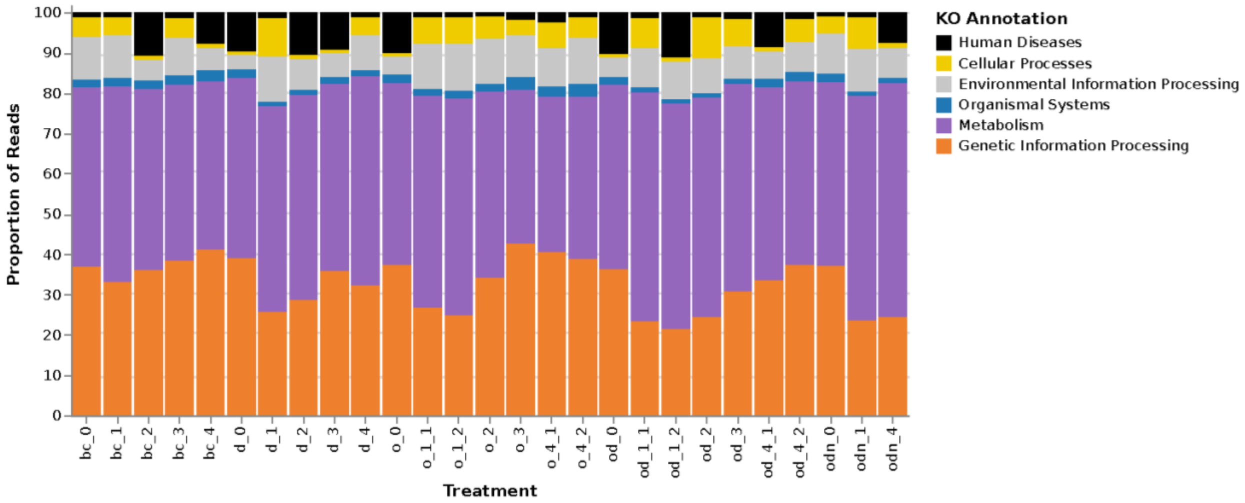

In [7]:

```
#Subset columns for QC figure
NOG_df=sk_df.loc[:,['sample_id','nog_cell_process_signaling','nog_info_s
torage_process', 'nog_metabolism', 'nog_poorly']]
plot_df = NOG_df
plot_df.columns=['Treatment','C','D','B','A']
#Normalizing table by column sums.
plot_df_2 = plot_df.drop('Treatment',axis=1)
vec = plot_df_2.sum(axis=1)
plot_df_2 = plot_df_2.div(plot_df_2.sum(axis=1),axis=0).mul(100)
plot_rel_df = plot_df['Treatment'].to_frame().join(plot_df_2)
the_leg = 'NOG Annotation'
the_leg_t = 'NOG Annotation'+':N'
the_y = 'Reads'
#melting table into long format
plot_df_longformat = pd.melt(plot_rel_df, id_vars=['Treatment'], var_nam
e=the_leg, value_name=the_y)

#Generating Plot
figS3C = alt.Chart(plot_df_longformat).mark_bar().encode(
alt.Color(the_leg_t, legend=alt.Legend(title=the_leg),
scale=alt.Scale(domain=['A', 'B', 'C', 'D'], range=['#c7c7c7','#000000',
'#EFCC00', '#1f77b4']),), alt.X('Treatment:N',
axis=alt.Axis(title='Treatment'),),y=the_y)

figS3C
```

Out[7]:

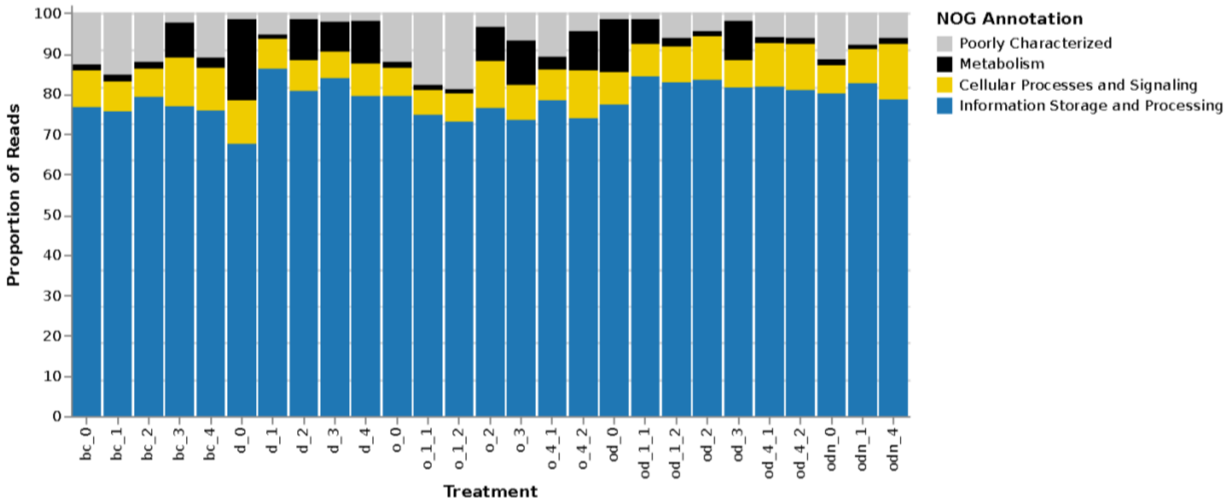

Figure 4

```
In [8]:
Location = r'subsystems_absolute.csv'
df = pd.read_csv(Location)
sorted_subsytems = df.iloc[:,2:29].sum(axis=0).sort_values(ascending=Fal
se).keys() #Here we sort from the biggest subsystem to the smallest
df_longformat = pd.melt(df, id_vars=['Time', 'Treatment'],var_name='Subs
ystem', value_name='Abundance')

numcols=5

all_categories = sorted_subsytems
rows=alt.vconcat(data=df_longformat)
numrows=int(np.ceil(len(all_categories) / numcols))
pointer=0
for _ in range(numrows):
      row=all_categories[pointer:pointer+numcols]
      cols=alt.hconcat()
      for a_chart in row:
      line = alt.Chart().mark_circle().encode(x='Time', y = 'Abundance',
color = alt.Color('Treatment', scale=alt.Scale(domain=['BC', 'D', 'O',
'OD', 'ODN'], range=['gray', 'red', 'blue', 'black', 'orange'])),
      size = 'Abundance').transform_filter(datum.Subsystem == a_ch
art).properties(title=a_chart, height=200, width=200)
      cols |= line
      rows &= cols
      pointer += numcols

figS4=rows
figS4
```

Out[8]:

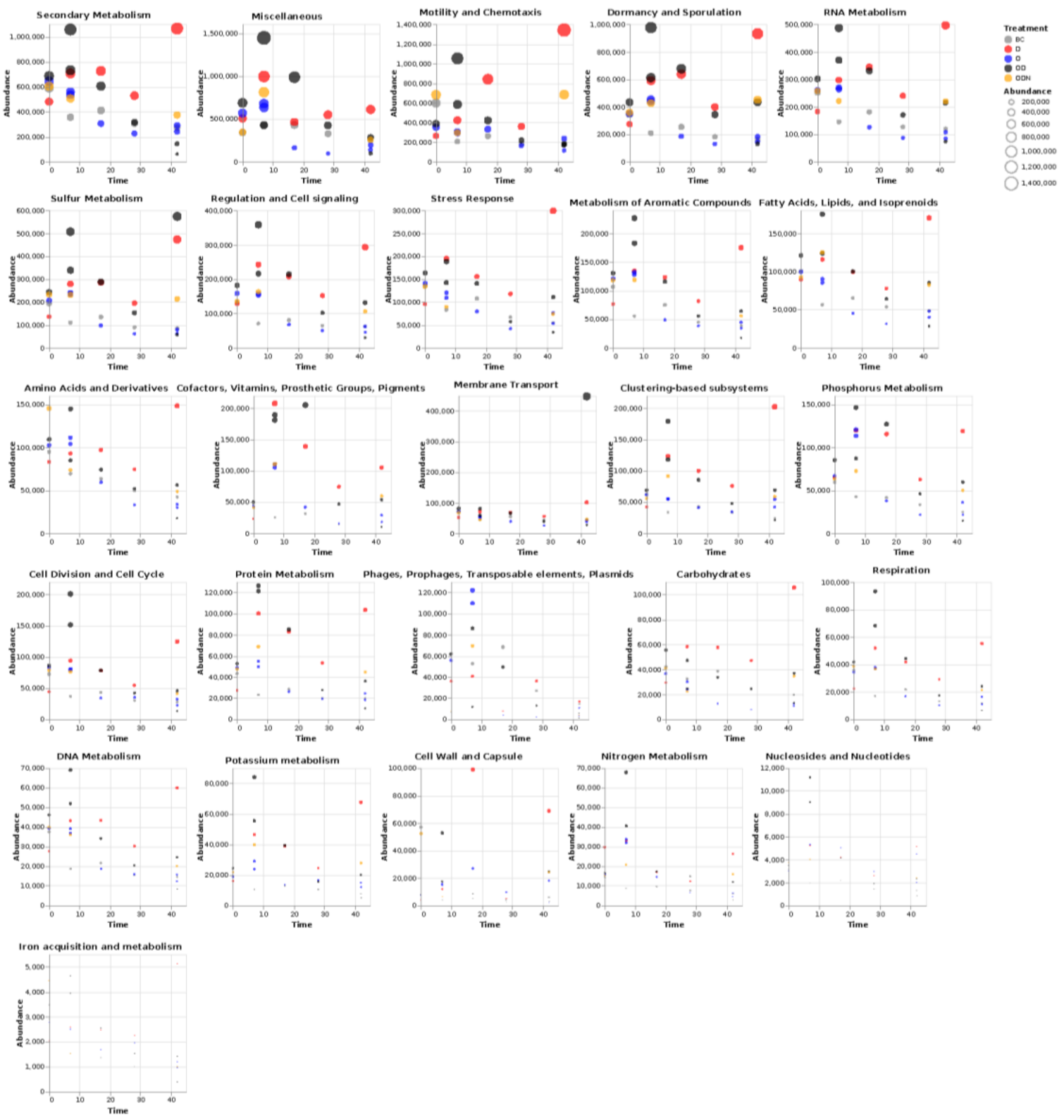

Supplementary Figure 4

For the following figure we are going to use normalized values by the highest value on each subsystem *S*.

$$\bar{x} = \frac{x}{\max(x)_S}$$

In [9]:

```
Location = r'subsystems_relative.csv'
df_rel = pd.read_csv(Location)

#Melting data
df_rel_longformat = pd.melt(df_rel, id_vars=['Time', 'Treatment'], var_n
ame='Subsystem', value_name='Relative Abundance')

df_longformat = df_rel_longformat
numcols=5
all_categories = sorted_subsytems
rows=alt.vconcat(data=df_longformat)
numrows=int(np.ceil(len(all_categories) / numcols))
pointer=0

for _ in range(numrows):
      row=all_categories[pointer:pointer+numcols]
      cols=alt.hconcat()
      for a_chart in row:
      line = alt.Chart().mark_circle().encode(x='Time', y='Relative
Abundance',
color = alt.Color('Treatment', scale=alt.Scale(domain=['BC', 'D', 'O',
'OD', 'ODN'], range=['gray', 'red', 'blue', 'black', 'orange'])), size =
'Relative Abundance').transform_filter(datum.Subsystem ==
a_chart).properties(title=a_chart, height=200, width=200)
      cols |= line
      rows &= cols
      pointer += numcols

figS5=rows
figS5
```

Out[9]:

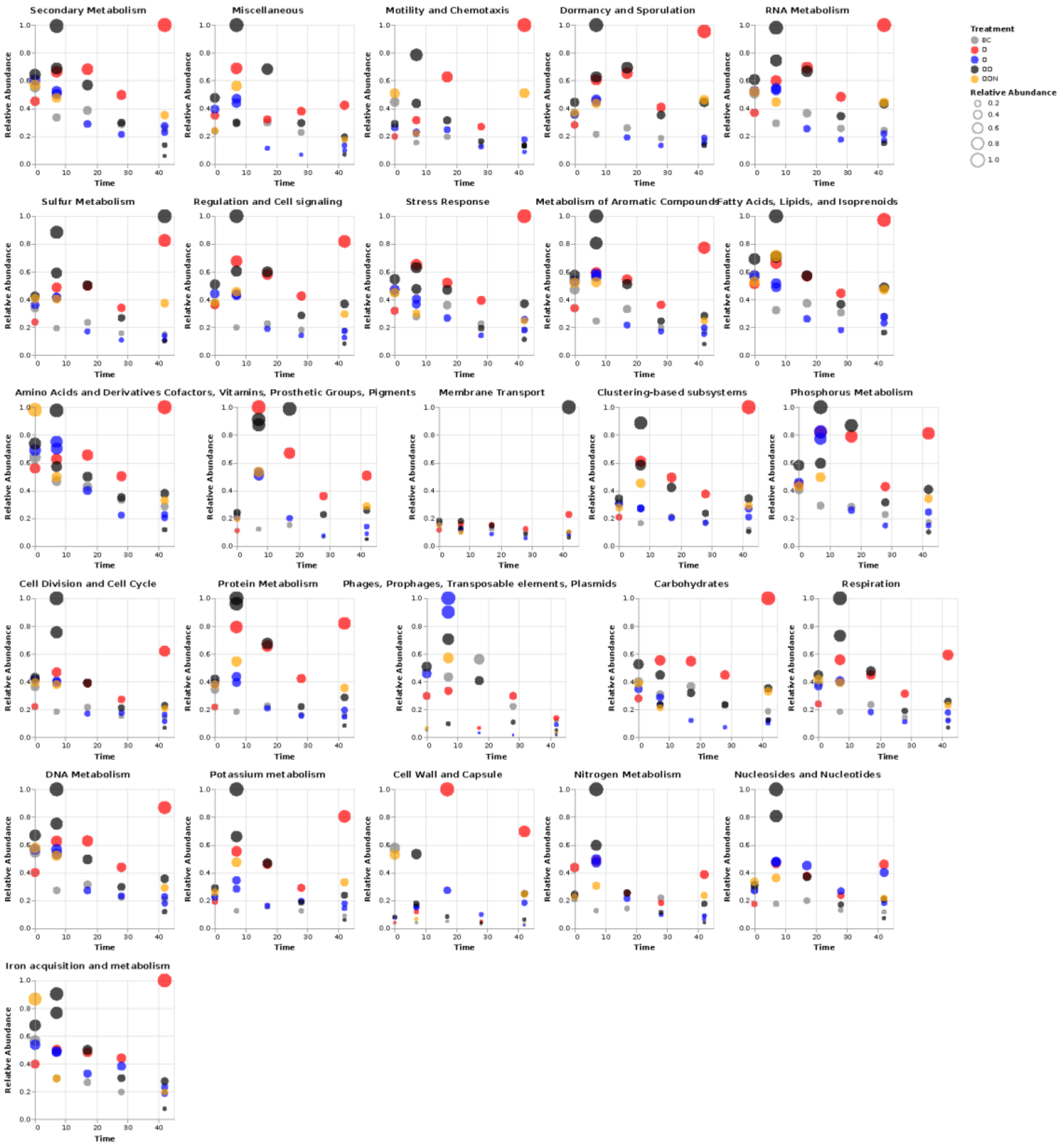

Secondary Metabolism
Miscellaneous
Motility and Chemotaxis
Dormancy and Sporulation
RNA Metabolism
Sulfur Metabolism
Regulation and Cell signaling
Stress Response
Metabolism of Aromatic Compounds
Fatty Acids, Lipids, and Isoprenoids
Amino Acids and Derivatives
Cofactors, Vitamins, Prosthetic Groups, Pigments
Membrane Transport
Clustering-based subsystems
Phosphorus Metabolism
Cell Division and Cell Cycle
Protein Metabolism
Phages, Prophages, Transposable elements, Plasmids
Carbohydrates
Respiration
DNA Metabolism
Potassium metabolism
Cell Wall and Capsule
Nitrogen Metabolism
Nucleosides and Nucleotides
Iron acquisition and metabolism
Relative Abundance
Time
Treatment
BC
D
O
OD
ODN
Relative Abundance
0.2
0.4
0.6
0.8
1.0

Figure 1

```
In [10]:
folders=['bc0', 'bc1', 'bc2', 'bc3', 'bc4', 'd0', 'd1', 'd2', 'd3', 'd4'
, 'o0', 'o1A', 'o1B', 'o2', 'o3', 'o4A', 'o4B', 'od0', 'od1A', 'od1B',
'od2', 'od3', 'od4A', 'od4B', 'odn0', 'odn1', 'odn4']
i =1
frames = []
for folder in folders:
      x_location = './' + (folder) + '/genus.csv'
      tax_df = pd.read_csv(x_location, header=None, sep='\t')
      tax_T = tax_df.T
      n = tax_T.shape[0]
      sample_vec = [i] * n
      d = {'species' : tax_T[0].values.tolist(), 'sample' : sample_vec ,
'counts' : tax_T[1].values.tolist() }
      tax_T_2 = pd.DataFrame(d)
      frames.append(tax_T_2)
      i+=1
mgrast_raw_counts = pd.concat(frames)
```

```
In [11]:
#reorder columns
mgrast_raw_counts = mgrast_raw_counts[['species','sample','counts']]
#printing to tab separated file
mgrast_raw_counts.to_csv("mgrast_raw_counts.txt", sep='\t', header=None,
index=None)
```

The following PERL script will use the annotation tags from the SEED system to find the complete Taxonomy tree from our curated taxonomic database.

From this we have the output file mgrast_raw_counts.txt_taxonomyXcounts.txt

The script is available at https://github.com/biotemon/GZ_et_al/tree/master/scripts

```
In [12]:
 #Download Perl script
 !rm mgrastrawcounts_to_goldDBtagscounts.pl
 !rm SK_metaT_v2.db.zip
 !rm SK_metaT_v2.db
 !wget https://github.com/biotemon/GZ_et_al/raw/master/scripts/mgrastrawc
ounts_to_goldDBtagscounts.pl
 !wget https://osf.io/s7u4t/download -O SK_metaT_v2.db.zip
 !unzip SK_metaT_v2.db.zip
 !cat mgrastrawcounts_to_goldDBtagscounts.pl | sed -e 's/SETDABASEHERE/
 \.\/SK\_metaT\_v2\.db/' > mgrastrawcounts_to_goldDBtagscounts_v2.pl
 !rm mgrastrawcounts_to_goldDBtagscounts.pl
 !mv mgrastrawcounts_to_goldDBtagscounts_v2.pl
mgrastrawcounts_to_goldDBt agscounts.pl
 #Run the code
 !perl mgrastrawcounts_to_goldDBtagscounts.pl mgrast_raw_counts.txt
```

--2020-09-03 12:28:08-- https://github.com/biotemon/GZ_et_al/raw/maste
r/scripts/mgrastrawcounts_to_goldDBtagscounts.pl
Resolving github.com (github.com)... 140.82.112.4
Connecting to github.com (github.com)|140.82.112.4|:443... connected. HTTP request sent, awaiting response... 302 Found
Location: https://raw.githubusercontent.com/biotemon/GZ_et_al/master/sc
ripts/mgrastrawcounts_to_goldDBtagscounts.pl [following]
--2020-09-03 12:28:08-- https://raw.githubusercontent.com/biotemon/GZ_
et_al/master/scripts/mgrastrawcounts_to_goldDBtagscounts.pl
Resolving raw.githubusercontent.com (raw.githubusercontent.com)... 199. 232.32.133
Connecting to raw.githubusercontent.com (raw.githubusercontent.com)|19 9.232.32.133|:443... connected.
HTTP request sent, awaiting response... 200 OK Length: 17972 (18K) [text/plain]
Saving to: 'mgrastrawcounts_to_goldDBtagscounts.pl'

mgrastrawcounts_to_ 100%[===================>] 17.55K --.-KB/s

in 0.01s

2020-09-03 12:28:08 (1.15 MB/s) - 'mgrastrawcounts_to_goldDBtagscounts. pl' saved [17972/17972]

--2020-09-03 12:28:08-- https://osf.io/s7u4t/download Resolving osf.io (osf.io)... 35.190.84.173
Connecting to osf.io (osf.io)|35.190.84.173|:443... connected. HTTP request sent, awaiting response... 302 FOUND
Location: https://files.osf.io/v1/resources/fu9bw/providers/dropbox/SK_
metaT_v_may_28_2019.db.zip?action=download&direct&version [following]
--2020-09-03 12:28:10-- https://files.osf.io/v1/resources/fu9bw/provid
ers/dropbox/SK_metaT_v_may_28_2019.db.zip?action=download&direct&versio n
Resolving files.osf.io (files.osf.io)... 35.186.214.196
Connecting to files.osf.io (files.osf.io)|35.186.214.196|:443... connec ted.
HTTP request sent, awaiting response... 200 OK Length: 90160523 (86M) [application/octet-stream]
Saving to: 'SK_metaT_v2.db.zip'

SK_metaT_v2.db.zip 100%[===================>] 85.98M 11.0MB/s

in 7.9s

2020-09-03 12:28:20 (10.8 MB/s) - 'SK_metaT_v2.db.zip' saved [90160523/

90160523]

Archive: SK_metaT_v2.db.zip
replace SK_metaT_v2.db? [y]es, [n]o, [A]ll, [N]one, [r]ename: ^C Determining taxonomic query terms
99%
[===
=
=======*]Updating taxonomy database and getting tax_ids
100%
[===
=
========]Processing taxonomyXcounts table
99%
[===
=
=======*]Processing add_manually_terms file

Here we generate a plot using a visualization threshold defined as the minimum percentage that a certain clade represents from the amount of reads in a sample. If the clade recruits an amount of reads less than the mentioned clade, those reads are merged with the corresponding parent in the taxonomic hierarchy.

In [13]:

```
#Download R script
!rm plot_CommunityStructure.R
!wget https://raw.githubusercontent.com/biotemon/K2015/master/scripts/pl
ot_CommunityStructure_vSK.R
!mv plot_CommunityStructure_vSK.R plot_CommunityStructure.R
```

--2020-09-05 18:41:41-- https://raw.githubusercontent.com/biotemon/K20
15/master/scripts/plot_CommunityStructure_vSK.R
Resolving raw.githubusercontent.com (raw.githubusercontent.com)... 199. 232.32.133
Connecting to raw.githubusercontent.com (raw.githubusercontent.com)|19 9.232.32.133|:443...
connected.
HTTP request sent, awaiting response... 200 OK Length: 17117 (17K) [text/plain]
Saving to: ‘plot_CommunityStructure_vSK.R’

plot_CommunityStruc 100%[===================>] 16.72K --.-KB/s

in 0.01s

2020-09-05 18:41:41 (1.26 MB/s) - 'plot_CommunityStructure_vSK.R' saved [17117/17117]

In [14]:

```
#Definition of some important variables
cwd = os.getcwd()
viz_threshold = '4'
#Concatenating names of samples
samples_string = '", "'.join(folders)
# Read in the file
with open('plot_CommunityStructure.R', 'r') as file : filedata = file.re
ad()
#Setting Desired Order
filedata = filedata.replace('SETDESIREDBARORDERHERE', samples_string)
#Setting order of libraries matching the desired order
filedata =
filedata.replace('SETSAMPLENUMBERSHERE','1","2","3","4","5","6","7","8","9
","10","11","12","13","14","15","16","17","18","19","20","21","22","23","2
4","25","26","27')
# Setting visualization threshold
filedata = filedata.replace('SETTHRESHOLDHERE', viz_threshold)
# Setting working directory
filedata = filedata.replace('SETWORKINGDIRHERE', cwd)
# Setting taxcounts file
filedata = filedata.replace('SETTAXCOUNTSFILEHERE',
'mgrast_raw_counts.txt_taxonomyXcounts.txt')

# Setting sample names
filedata = filedata.replace('SETSAMPLENAMESHERE', samples_string)
colortochange = "\n".join([  'simple_color_vec[2] <- "#d1d1d1"',
            'simple_color_vec[2] <- "#512888"',
            'simple_color_vec[3] <- "#D8BFD8"',
            'simple_color_vec[4] <- "#cc12c0"',
            'simple_color_vec[5] <- "#9E0142"',
            'simple_color_vec[6] <- "#ff9e9e"',
            'simple_color_vec[7] <- "#DC143C"',
            'simple_color_vec[8] <- "#ff6f00"',
            'simple_color_vec[9] <- "#FFEF00"',
            'simple_color_vec[10] <- "#FFD700"',
            'simple_color_vec[11] <- "#D2691E"',
            'simple_color_vec[12] <- "#008000"',
            'simple_color_vec[13] <- "#8db500"',
            'simple_color_vec[14] <- "#ACE1AF"',
```

```
               'simple_color_vec[15] <- "#e6ffe6"',
               'simple_color_vec[16] <- "#4B6F44"',
               'simple_color_vec[17] <- "#72996c"',
               'simple_color_vec[18] <- "#A8E224"',
               'simple_color_vec[19] <- "#008080"',
               'simple_color_vec[20] <- "#a3cece"',
               'simple_color_vec[21] <- "#118200"',
               'simple_color_vec[22] <- "#CAD32E"',
               'simple_color_vec[23] <- "#DFFF00"',
               'simple_color_vec[24] <- "#f8fcde"',
               'simple_color_vec[25] <- "#6CB4EE"',
               'simple_color_vec[26] <- "#0000FF"',
               'simple_color_vec[27] <- "#0087BD"',
               'simple_color_vec[28] <- "#e1e3e8"',
               'simple_color_vec[29] <- "#7a7a7a"',
               ])
filedata = filedata.replace('#SETCOLORSHERE', colortochange)
# Write the file out again
with open('plot_CommunityStructure.R', 'w') as file: file.write(filedata)
```

```
In [15]: #Run the R script
!Rscript plot_CommunityStructure.R
```

Attaching package: ‘dplyr’

The following objects are masked from ‘package:plyr’:

arrange, count, desc, failwith, id, mutate, rename, summarise, summarize

The following objects are masked from ‘package:stats’: filter, lag

The following objects are masked from ‘package:base’: intersect, setdiff, setequal, union

Warning message:
In data.table::melt(simple_absolute_matrix_3, id.vars = "sample_names")
:
The melt generic in data.table has been passed a data.frame and will attempt to redirect to the relevant reshape2 method; please note that r eshape2 is deprecated, and this redirection is now deprecated as well. To continue using melt methods from reshape2 while both libraries are a ttached, e.g. melt.list, you can prepend the namespace like reshape2::m elt(simple_absolute_matrix_3). In the

next version, this warning will b ecome an error.
Warning message:
In data.table::melt(simple_relative_matrix_3, id.vars = "sample_names")
:
The melt generic in data.table has been passed a data.frame and will attempt to redirect to the relevant reshape2 method; please note that r eshape2 is deprecated, and this redirection is now deprecated as well. To continue using melt methods from reshape2 while both libraries are a ttached, e.g. melt.list, you can prepend the namespace like reshape2::m elt(simple_relative_matrix_3). In the next version, this warning will b ecome an error.

null device
1
null device
1
null device
1
null device
1

In [16]:

```
!mv simple_relative_melt.csv fig1_simple_relative_melt.csv
!mv simple_absolute_melt.csv fig1_simple_absolute_melt.csv
```

In [51]:

```
viz_threshold = '4'
svg_relative_file = "taxonomy_rel_cutoff_" + viz_threshold + ".svg"
display(SVG(svg_relative_file))
```

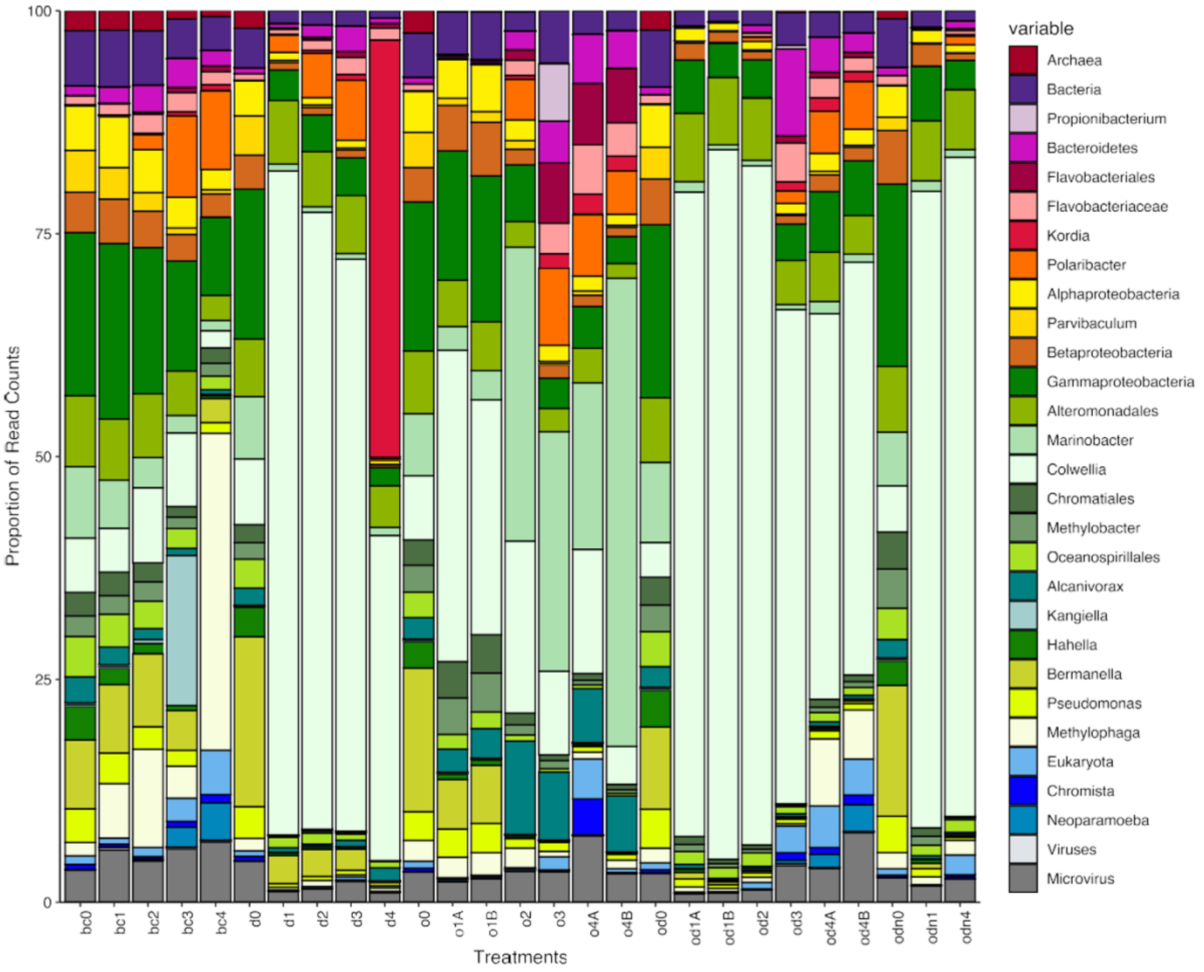

Proportion of Read Counts
100
75
50
25
0
bc0
bc1
bc2
bc3
bc4
d0
d1
d2
d3
d4
o0
o1A
o1B
o2
o3
o4A
o4B
od0
od1A
od1B
od2
od3
od4A
od4B
odn0
odn1
odn4
Treatments
variable
Archaea
Bacteria
Propionibacterium
Bacteroidetes
Flavobacteriales
Flavobacteriaceae
Kordia
Polaribacter
Alphaproteobacteria
Parvibaculum
Betaproteobacteria
Gammaproteobacteria
Alteromonadales
Marinobacter
Colwellia
Chromatiales
Methylobacter
Oceanospirillales
Alcanivorax
Kangiella
Hahella
Bermanella
Pseudomonas
Methylophaga
Eukaryota
Chromista
Neoparamoeba
Viruses
Microvirus

Supplementary Figure 3

```
In [17]:
folders=['bc0', 'bc1', 'bc2', 'bc3', 'bc4', 'd0', 'd1', 'd2', 'd3', 'd4'
, 'o0','o1A', 'o1B','o2', 'o3', 'o4A', 'o4B', 'od0', 'od1A', 'od1B', 'od
2', 'od3','od4A', 'od4B', 'odn0', 'odn1', 'odn4']
i =1
j =0
k =0
rep =0
frames = []
for folder in folders:
      x_location = './'+ (folder) + '/rarefaction.csv'
      rar_df = pd.read_csv(x_location, sep='\t')
      n = rar_df.shape[0]
      if (folder == 'bc0') or (folder == 'd0') or (folder == 'o0') or (fol
der == 'od0') or (folder == 'odn0'):
            l = '00'
            rep = 1
      if (folder == 'bc1') or (folder == 'd1') or (folder == 'o1A') or
(folder == 'od1A') or (folder == 'odn1'):
            l = '07'
            rep = 1
      if (folder == 'o1B') or (folder == 'od1B'):
            l = '07'
            rep = 2
      if (folder == 'bc2') or (folder == 'd2') or (folder == 'o2') or
(folder == 'od2'):
            l = '17'
            rep = 1
      if (folder == 'bc3') or (folder == 'd3') or (folder == 'o3') or
(folder == 'od3'):
            l = '28'
            rep = 1
      if (folder == 'bc4') or (folder == 'd4') or (folder == 'o4A') or
(folder == 'od4A') or (folder == 'odn4'):
            l = '42'
            rep = 1
      if (folder == 'o4B') or (folder == 'od4B'):
            l = '42'
            rep = 2
      if (folder == 'bc0') or (folder == 'bc1') or (folder == 'bc2') or
(folder == 'bc3') or (folder == 'bc4'):
            m = "Biotic Control"
      if (folder == 'd0') or (folder == 'd1') or (folder == 'd2') or
(folder == 'd3') or (folder == 'd4'):
            m = "Dispersants"
      if  (folder  ==  'o0')  or  (folder  ==  'o1A')  or  (folder  ==  'o1B')  or
(folder == 'o2') or (folder == 'o3') or (folder == 'o4A') or (folder == 'o4
B'):
            m = "Oil"
      if (folder == 'od0') or (folder == 'od1A') or (folder == 'od1B') or
(folder == 'od2') or (folder == 'od3') or (folder == 'od4A') or (folder
== 'od4B'):
```

```
            m = "Oil+Dispersants"
      if (folder == 'odn0') or (folder == 'odn1') or (folder == 'odn4'): m
= "OD+Nutrients"
            d = {'Species Counts' : rar_df['species
      count'].values.tolist(), 'Ti me days' : l , 'Treatment' : m,'Reads'
      : rar_df['number of reads'].values.tolist(), ‘Replicate’ : rep}
      rar_df_2 = pd.DataFrame(d)
      frames.append(rar_df_2)
```

In [18]:

```
rarefaction_df = pd.concat(frames)
rarefaction_df.Reads = rarefaction_df.Reads.div(1000000)
rarefaction_df.rename(columns={'Reads': 'Reads (millions)'}, inplace=True)
rarefaction_df_smaller = rarefaction_df.iloc[::10, :]
temp = rarefaction_df_smaller[rarefaction_df_smaller['Reads
(millions)']>13]
```

In [19]:

```
alt.data_transformers.enable('csv')
alt.renderers.enable('jupyterlab')
df_longformat = rarefaction_df_smaller
numcols=2 # specify the number of columns you want
all_categories=df_longformat['Treatment'].unique()

rows=alt.vconcat(data=df_longformat)
numrows=int(np.ceil(len(all_categories) / numcols))
pointer=0

for _ in range(numrows):
     row=all_categories[pointer:pointer+numcols]
     cols=alt.hconcat()
     for a_chart in row:
           line = alt.Chart().mark_circle().encode( x='Reads (millions):Q',
     y='Species Counts:Q', color='Time
     days:Q').transform_filter(datum.Treatment == a_c
     hart).properties(title=a_chart, height=200, width=200)
           cols |= line
      rows &= cols
      pointer += numcols
 figS4=rows
 figS4
```

Out[7]:

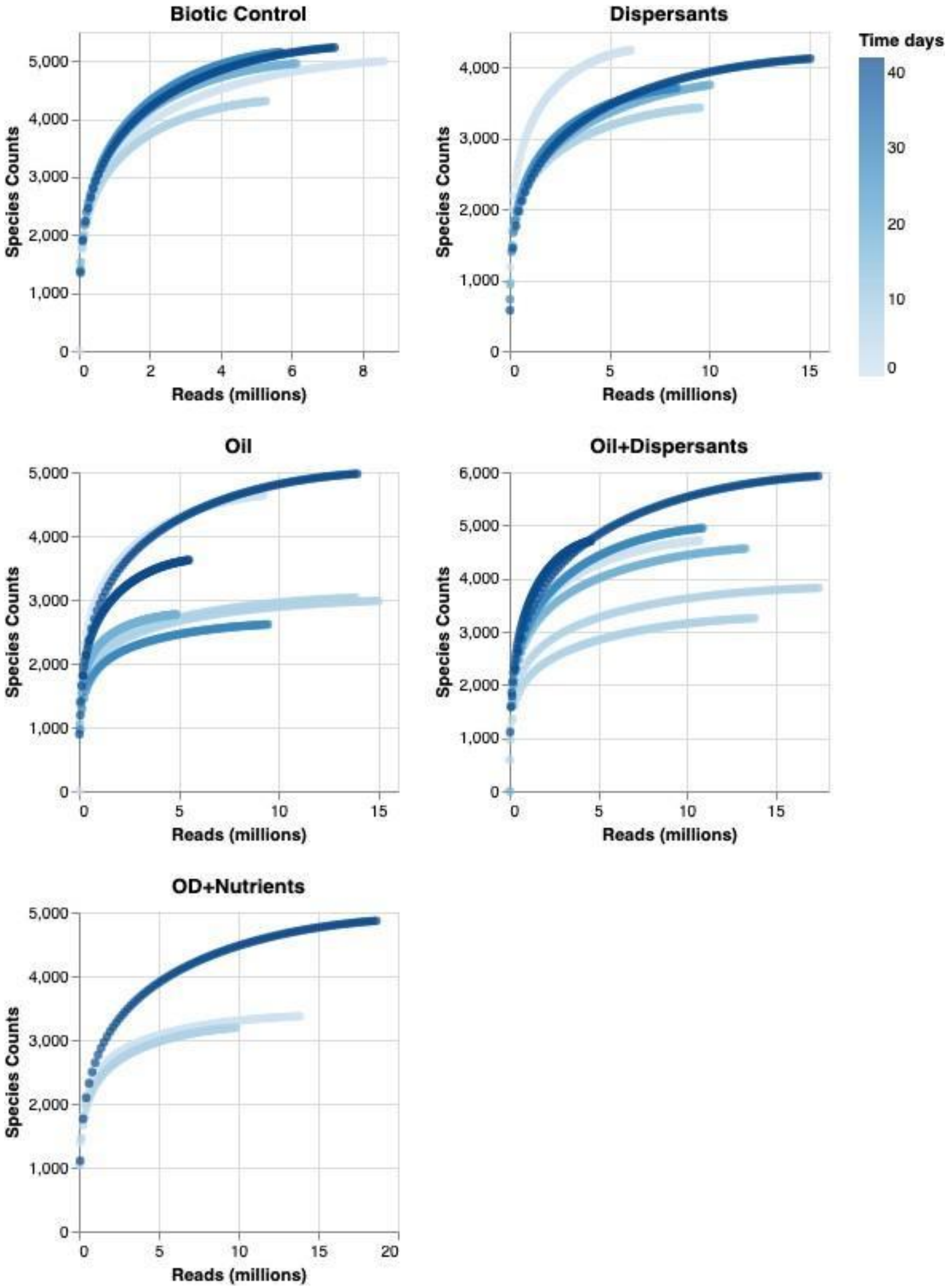

Biotic Control
Dispersants
Oil
Oil+Dispersants
OD+Nutrients
Species Counts
Reads (millions)
Time days
0
10
20
30
40

Alpha diversity

We are generating the plot with changes in alpha diversity. Let's remember that the alpha diversity estimate is a single number that summarizes the distribution of species-level annotations in a dataset. The Shannon diversity index is an abundance-weighted average of the logarithm of the relative abundances of annotated species. We compute the species richness as the antilog of the Shannon diversity:

$$\alpha = 10^{\sum_i p_i \log p_i}$$

where p_i are the proportions of annotations in each of the species categories.

In [20]:

```
#Subset columns for QC figure
ad_df=sk_df.loc[:,['sample_id','alpha_diversity']]
vec_1 = pd.DataFrame({'Time days':[0,7,17,28,42,0,7,17,28,42,0,7,7,17,28
,42,42,0,7,7,17,28,42,42,0,7,42]})
vec_2 = pd.DataFrame({'Treatment' : ['bc', 'bc', 'bc', 'bc', 'bc', 'd',
'd', 'd', 'd', 'd', 'o', 'o', 'o', 'o', 'o', 'o',
'o','od','od','od','od','od','od','od', 'odn', 'odn', 'odn']})
ad_df1 = ad_df.join(vec_1).join(vec_2)

#Linear regression of all data points together
x=ad_df1['Time days']
x=sm.add_constant(x)
y=ad_df1.alpha_diversity
regr = sm.OLS(y, x)
res = regr.fit()

# Get fitted values from model to plot
st, data, ss2 = summary_table(res, alpha=0.05)
fitted_values_df = pd.DataFrame({'fitted_values':data[:,2]})

# Get the confidence intervals of the model

ci_values_df = pd.DataFrame(data[:,4:6])
ad_df2 = x.join(y).join(fitted_values_df).join(ci_values_df)
ad_df2.columns= ['const', 'Time days', 'alpha_diversity',
'fitted_values','ci_down', 'ci_up']
ad_df3 = ad_df2.loc[:,['Time days','ci_down', 'ci_up']]
ad_df3.drop_duplicates(inplace=True)
ad_df3.sort_values(by='Time days', inplace=True)


part_1 = alt.Chart(ad_df1).mark_point().encode(x='Time days',
y='alpha_diversity',
         color = alt.Color('Treatment', scale=alt.Scale(domain=['bc', 'd',
'o', 'od', 'odn'], range=['gray', 'red', 'blue', 'black', 'darkorange'])),
).properties(height=200, width=200)
```

```
part_2 = alt.Chart(ad_df2).mark_line(strokeDash=[10,4], color='gray', st
rokeWidth = 5, strokeOpacity = 0.3).encode(x='Time days',
y='fitted_values',
        ).properties(height=200, width=200)


part_3 = alt.Chart(ad_df3).mark_area(opacity=0.1).encode(x='Time days',
         y=alt.Y('ci_down',axis=alt.Axis(title='alpha_diversity')),
y2='ci_up'
         ).properties(height=200, width=200)
plot_xx = part_1 + part_2 + part_3
#Creating a loop of linear regressions for each treatment category # Def
ine the degree of the polynomial fit

treatment_list = ['bc', 'd', 'o', 'od', 'odn']
colors = ['gray', 'red', 'blue', 'black', 'darkorange']
i=0
for treat in treatment_list:
      slice_df = ad_df1[(ad_df1['Treatment']==treat)]
      x=slice_df['Time days']
      x=sm.add_constant(x)
      y=slice_df.alpha_diversity
      regr = sm.OLS(y, x)
      res = regr.fit()
      # Get fitted values from model to plot
      st, data, ss2 = summary_table(res, alpha=0.05)
      fitted_values_df = pd.DataFrame({'fitted_values':data[:,2]})
      x = x.reset_index(drop=True)
      melted_df = pd.concat([fitted_values_df,x],axis=1)
      plot_x = alt.Chart(melted_df).mark_line(color=colors[i]).encode(x='T
ime days', y='fitted_values').properties(height=200, width=200)
      plot_xx = plot_xx + plot_x
      i = i+1

fig8 = plot_xx
fig8
```

Out[20]:

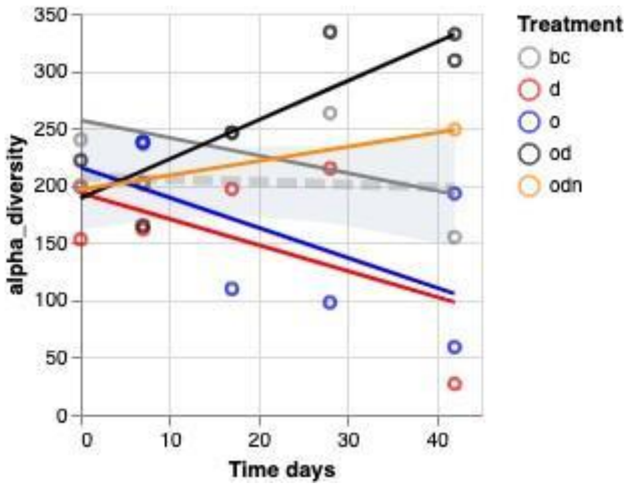

Supplementary Figure 7

We will define some functions. We will use them frequently in this section.

```
In [21]:
def get_important_features(transformed_features, components_, columns):
      """
      This function will return the most "important" features so we can
      determine which have the most effect on multi-dimensional scaling
      """
      num_columns = len(columns)
      # Scale the principal components by the max value in
      # the transformed set belonging to that component
      xvector = components_[0] * max(transformed_features[:,0])
      yvector = components_[1] * max(transformed_features[:,1])

      # Sort each column by it's length. These are your *original*
      # columns, not the principal components.
      important_features = {columns[i] : math.sqrt(xvector[i]**2 + yvecto
r[i]**2) for i in range(num_columns)}
      important_features = sorted(zip(important_features.values(),
important_features.keys()), reverse=True)
      return(important_features)

In [22]:
def draw_vectors(transformed_features, components_, columns,
max_num_vectors):
    """
    This function will project your *original* features
    onto your principal component feature-space, so that you can visualize
    how "important" each one was in the
    multi-dimensional scaling
    """

    num_columns = len(columns)

    #Scale the principal components by the max value in # the transformed
    set belonging to that component
    n0vector = [0] * num_columns
    x1vector = components_[0] * max(transformed_features[:,0])
    y1vector = components_[1] * max(transformed_features[:,1])

    #Calculate euclidean distance
    disvector = np.sqrt(np.square(x1vector - n0vector) + np.square(y1vec
    tor - n0vector))
    s1 = pd.Series(columns)
    s2 = pd.Series(x1vector)
    s3 = pd.Series(y1vector)
    s4 = pd.Series(disvector)

    arrows_df = pd.DataFrame([s1, s2, s3, s4])
    arrows_df = arrows_df.transpose()
    arrows_df.columns = ['label','x','y', 'D']
```

```
    arrows_df = arrows_df.sort_values(by=['D'], ascending=False)
    arrows_df = arrows_df.head(max_num_vectors)

    n0vector = [0] * max_num_vectors

    s1 = pd.Series(arrows_df['label']).reset_index(drop=True)
    s2 = pd.Series(n0vector)
    s3 = pd.Series(n0vector)
    s4 = pd.Series(arrows_df['D']).reset_index(drop=True)

    arrows_2_df = pd.DataFrame([s1, s2, s3, s4])
    arrows_2_df = arrows_2_df.transpose()
    arrows_2_df.columns = ['label','x','y', 'D']
    frames = [arrows_df, arrows_2_df]
    arrows_3_df = pd.concat(frames)
    return arrows_3_df
```

In [23]:

```
def pca_subsystems(the_dataframe, max_num_vectors):
      y = the_dataframe.index.values
      x = the_dataframe.values
      xx = StandardScaler().fit_transform(x)
      the_pca = PCA(n_components=2) the_pca.fit(the_dataframe)
      the_T = the_pca.transform(the_dataframe)
      the_explainedvector = the_pca.explained_variance_ratio_
      the_components = pd.DataFrame(the_pca.components_, columns = the_dat
      aframe.columns, index=[1, 2])
      the_important_features = get_important_features(the_T, the_pca.compo
      nents_,the_dataframe.columns.values)
      the_ax = draw_vectors(the_T, the_pca.components_, the_dataframe.colu
      mns.values, max_num_vectors)
      the_T_df = pd.DataFrame(the_T)
      the_T_df.columns = ['x', 'y']
      d = {'Treatment': ['BC', 'BC', 'BC', 'BC', 'BC', 'D', 'D', 'D', 'D',
      'D', 'ODN', 'ODN', 'ODN', 'OD', 'OD', 'OD', 'OD', 'OD', 'OD', 'OD',
      'O', 'O', 'O', 'O', 'O', 'O', 'O'], 'Time': ['10', '17', '27', '38',
      '52', '10', '17', '27', '38', '52', '10', '17', '52', '10', '17',
      '17', '27', '38', '52', '52', '10', '17', '17', '27', '38', '52',
      '52']}
      temp_df = pd.DataFrame(data=d)
      the_T_df = the_T_df.join(temp_df)
      return (the_explainedvector, the_important_features, the_ax,
      the_T_df)
```

In [24]:

```
#Loading data
Location = './SK_subsystems_level_1_WITH_REPS.tsv'

#Processing
input_df = pd.read_csv(Location, sep= '\t', error_bad_lines=False)
input_df['samples'] = input_df.index
```

```
input_df_1 = input_df.loc[:,['samples', 'dataset', 'abundance']].pivot(i
ndex='samples', columns='dataset')['abundance']
my_expl, my_important, my_ax, my_Tdf = pca_subsystems(input_df_1, 10)
```

In [25]:

```
vectorpart1 = alt.Chart(my_ax).mark_line().encode(x='x', y='y',
color=alt.Color('label', legend=None))
vectorpart2 = alt.Chart(my_ax).mark_line().encode(x='x', y='y')
vectortext_1 = vectorpart2.mark_text( align='left',
baseline='middle', dx=7).encode(text='label')

scale = alt.Scale(domain=['Amino Acids and Derivatives', 'BC', 'Carbohyd
rates', 'Clustering-based subsystems', 'Cofactors, Vitamins, Prosthetic
Groups, Pigments', 'D', 'Membrane Transport', 'Miscellaneous', 'Motility
and Chemotaxis', 'O', 'OD', 'ODN', 'Protein Metabolism', 'RNA Metabolis
m', 'Respiration'],
range=['black', 'gray', 'black', 'black', 'black', 'red', 'black',
'black', 'black', 'blue', 'black', 'darkorange', 'black', 'black',
'black'])
dotspart = alt.Chart(my_Tdf).mark_point().encode(x='x', y='y',
color=alt.Color('Treatment', legend=alt.Legend(title='Treatment'), s
cale=scale),
size='Time:Q')

figS6A = dotspart + vectorpart1 + vectortext_1
figS6A
```

Out[25]:

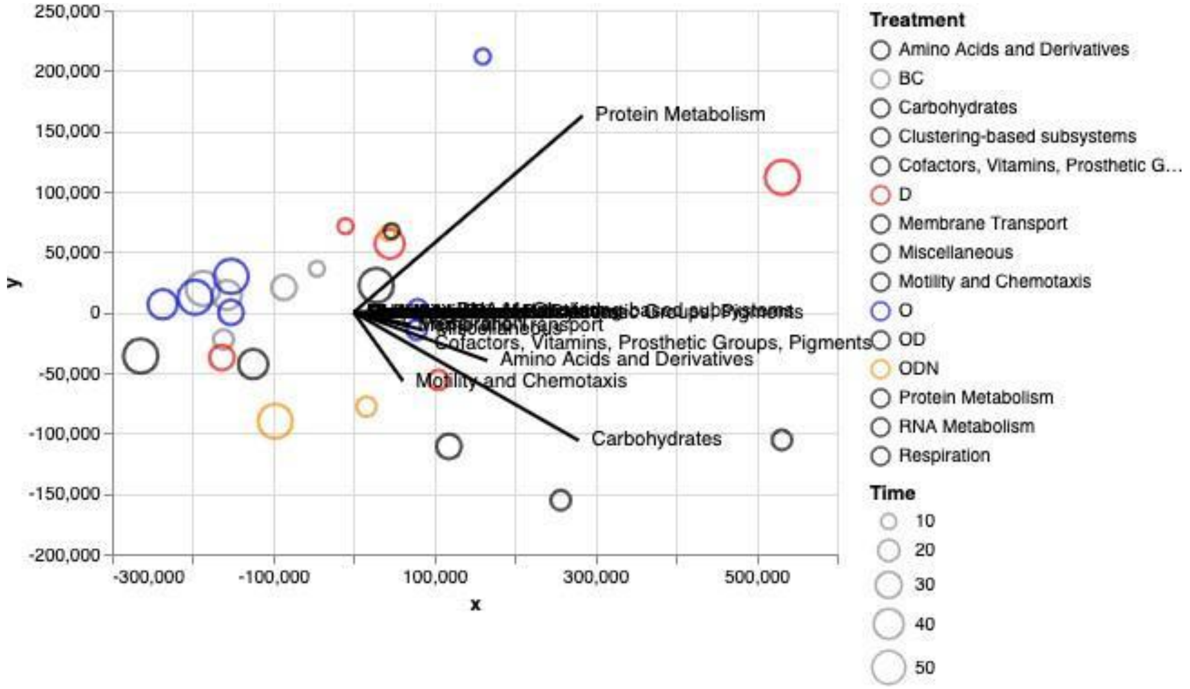

In [26]:

```
Location = r'./SK_subsystems_level_2_WITH_REPS.tsv'
input_df = pd.read_csv(Location, sep= '\t', error_bad_lines=False).reset
```

```
_index()
input_df_1 = input_df.loc[:,['level_0', 'dataset',
'abundance']].pivot(index='level_0', columns='dataset')['abundance']
input_df_1 = input_df_1.loc[:, input_df_1.columns.notnull()]
input_df_1 = input_df_1.fillna(0)

my_expl, my_important, my_ax, my_Tdf = pca_subsystems(input_df_1, 10)
vectorpart1 = alt.Chart(my_ax).mark_line().encode(x='x',y='y',
color=alt.Color('label', legend=None))
vectorpart2 = alt.Chart(my_ax).mark_line().encode(x='x',y='y')
vectortext_1 = vectorpart2.mark_text(align='left',baseline='middle', dx=7
).encode(text='label')

scale = alt.Scale(domain=['BC', 'Branched-chain amino acids', 'Central
carbohydrate metabolism', 'D', 'Fermentation', 'Flagellar motility in
Prokaryota', 'O', 'OD', 'ODN','Organic acids', 'Plant-Prokaryote DOE
project', 'Protein biosynthesis', 'Protein folding', 'RNA processing and
modif ication', 'Transcription'],
range=['gray', 'black', 'black', 'red', 'black', 'black', 'blue', 'black',
'darkorange', 'black', 'black', 'black', 'black', 'black', 'black'])

dotspart = alt.Chart(my_Tdf).mark_point().encode(x='x', y='y',
color=alt.Color('Treatment', legend=alt.Legend(title='Treatment'),
scale=scale),
size='Time:Q')

figS6B = dotspart + vectorpart1 + vectortext_1
figS6B
```

Out[26]:

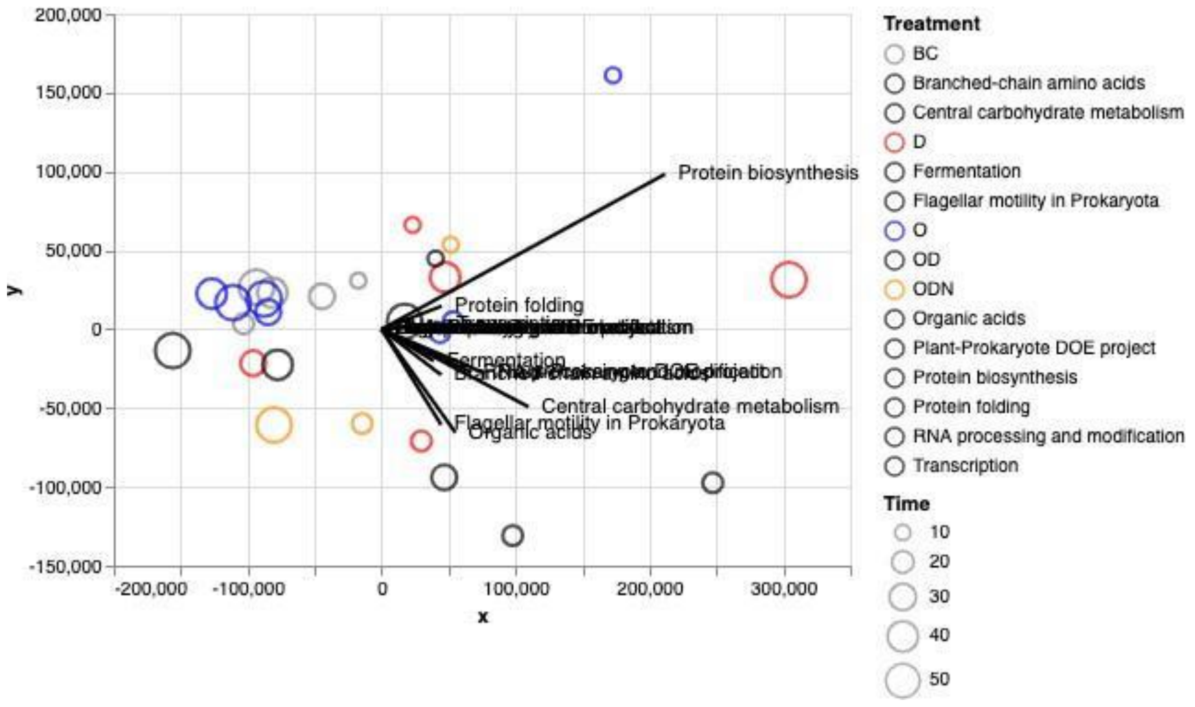

In [27]:

```
#Inspection of detailed table
Location = r'./SK_subsystems_level_3_WITH_REPS.tsv'

#Processing
input_df = pd.read_csv(Location, sep= '\t', error_bad_lines=False, encod
ing = "ISO-8859-1").reset_index()
input_df_1 = input_df.loc[:, ['level3', 'BC_0', 'BC_1', 'BC_2', 'BC_3',
'BC_4','D_0', 'D_1', 'D_2', 'D_3', 'D_4', 'ODN_0', 'ODN_1', 'ODN_4', 'OD
_0', 'OD_1_1', 'OD_1_2', 'OD_2', 'OD_3', 'OD_4_1', 'OD_4_2', 'O_0', 'O_1
_1', 'O_1_2', 'O_2', 'O_3', 'O_4_1', 'O_4_2']]
input_df_1 = input_df_1.set_index(['level3'])
input_df_1 = input_df_1.T
my_expl, my_important, my_ax, my_Tdf = pca_subsystems(input_df_1, 10)

vectorpart1 = alt.Chart(my_ax).mark_line().encode(x='x', y='y',
color=alt.Color('label', legend=None))
vectorpart2 = alt.Chart(my_ax).mark_line().encode(x='x', y='y')
vectortext_1 = vectorpart2.mark_text( align='left',
baseline='middle', dx=7).encode(text='label')

scale = alt.Scale(domain=['BC', 'D', 'Flagellar_motility', 'Flagellum',
'Glyoxylate_bypass', 'Methylcitrate_cycle', 'O', 'OD', 'ODN',
'Propionate-
CoA_to_Succinate_Module','Protein_Acetylation_and_Deacetylation_in_Bacteri
a', 'Protein_chaperones', 'RNA_polymerase_bacterial',
'Ribosome_LSU_bacterial', 'Ribosome_SSU_bacterial'],
range=['gray', 'red', 'black', 'black', 'black', 'black',
'blue', 'black', 'darkorange', 'black', 'black', 'black',
'black', 'black', 'black'])

dotspart = alt.Chart(my_Tdf).mark_point().encode(x='x',y='y',
color=alt.Color('Treatment', legend=alt.Legend(title='Treatment'), s
cale=scale), size='Time:Q')

figS6C = dotspart + vectorpart1 + vectortext_1
figS6C
```

Out[27]:

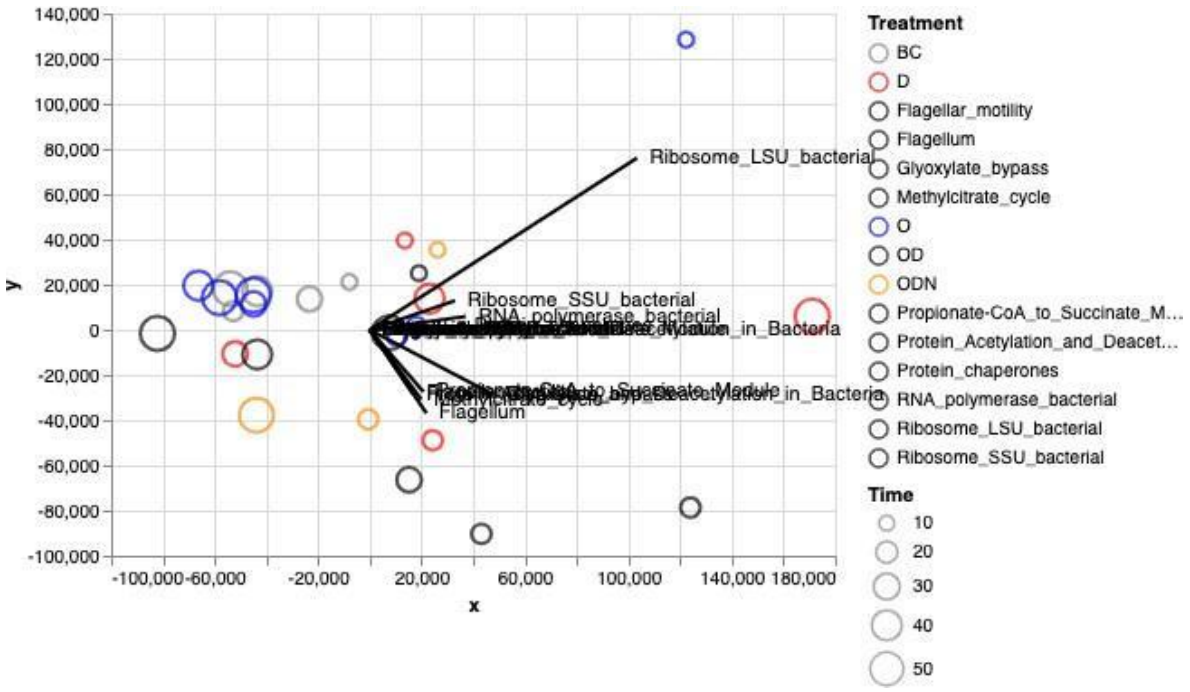

In [28]:

```
Location = r'./SK_subsystems_level_4_WITH_REPS.tsv'
input_df = pd.read_csv(Location, sep= '\t', error_bad_lines=False, encod
ing ="ISO-8859-1").reset_index()
input_df_1 = input_df.loc[:, ['function', 'BC_0', 'BC_1', 'BC_2', 'BC_3'
, 'BC_4', 'D_0', 'D_1', 'D_2', 'D_3', 'D_4', 'ODN_0', 'ODN_1', 'ODN_4',
'OD_0', 'OD_1_1', 'OD_1_2', 'OD_2', 'OD_3', 'OD_4_1', 'OD_4_2', 'O_0',
'O_1_1', 'O_1_2', 'O_2', 'O_3', 'O_4_1', 'O_4_2']]
input_df_1 = input_df_1.set_index(['function'])
input_df_1 = input_df_1.T
my_expl, my_important, my_ax, my_Tdf = pca_subsystems(input_df_1, 10)
vectorpart1 = alt.Chart(my_ax).mark_line().encode(x='x', y='y',
color=alt.Color('label', legend=None))

vectorpart2 = alt.Chart(my_ax).mark_line().encode(x='x',y='y')

vectortext_1 = vectorpart2.mark_text(align='left',
baseline='middle', dx=7).encode(text='label')

scale = alt.Scale(domain=['Acetyl-coenzyme A synthetase (EC 6.2.1.1)',
'BC', 'Chaperone protein DnaK', 'Cof protein
_ HD superfamily hydrolase', 'DNA-directed RNA polymerase beta subunit (EC
2.7.7.6)', 'D', 'Heat shock protein 60 family chaperone GroEL','Isocitrate
lyase (EC 4.1.3.1)', 'LSU ribosomal protein L1p (L10Ae)', 'O', 'OD',
'ODN', 'Pyruvate formate-lyase (EC 2.3.1.54)', 'TonB-dependent receptor'],
range=['black', 'gray', 'black', 'black', 'red', 'black', 'black',
'black', 'black', 'blue', 'black', 'darkorange', 'black', 'black'])

dotspart = alt.Chart(my_Tdf).mark_point().encode(x='x',y='y',
color=alt.Color('Treatment', legend=alt.Legend(title='Treatment'),
```

```
scale=scale), size='Time:Q')

figS6D = dotspart + vectorpart1 + vectortext_1

figS6D
```

Out[28]:

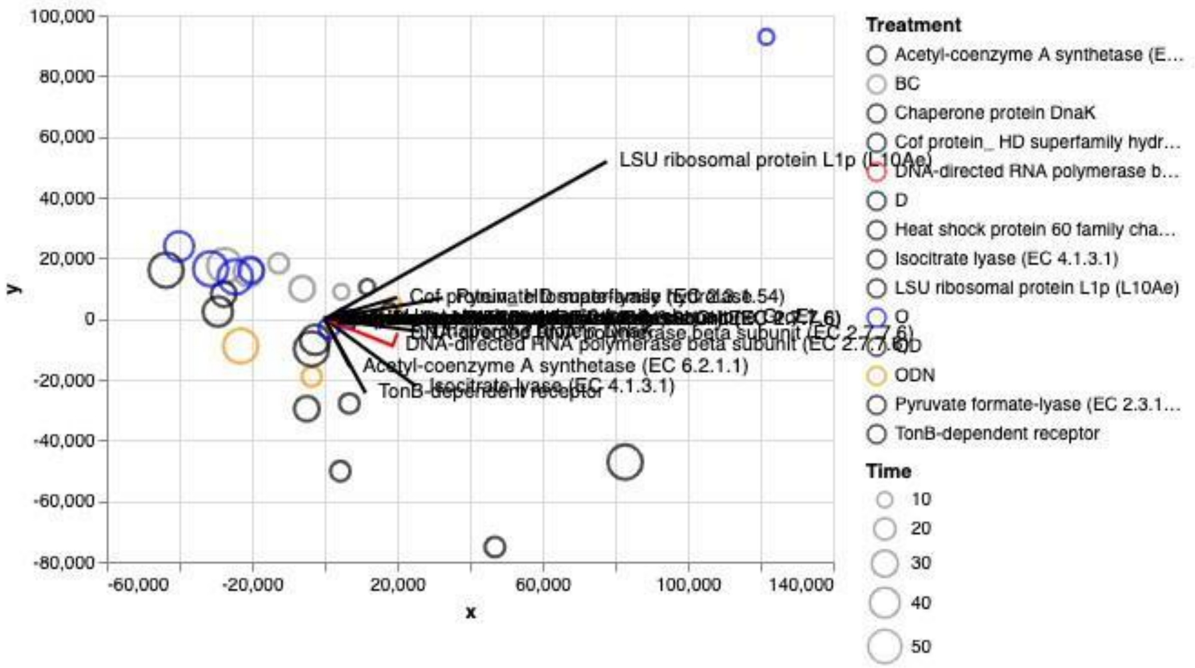

In [29]:

```
#Inspection of detailed table
Location = r'./SK_subsystems_level_2_WITH_REPS.tsv'
input_df = pd.read_csv(Location, sep= '\t', error_bad_lines=False).reset
_index()
input_df = input_df.loc[(input_df['level_1'] == 'Motility and Chemotaxi
s') | (input_df['level_1'] == 'Carbohydrates') | (input_df['level_1'] ==
'Membrane Transport') | (input_df['level_1'] == 'Respiration') ]
input_df_1 = input_df.loc[:,['level_0', 'dataset', 'abundance']].pivot(i
ndex='level_0', columns='dataset')['abundance']
input_df_1 = input_df_1.loc[:, input_df_1.columns.notnull()]

my_expl, my_important, my_ax, my_Tdf = pca_subsystems(input_df_1, 10)

vectorpart1 = alt.Chart(my_ax).mark_line().encode(x='x', y='y',
color=alt.Color('label', legend=None))

vectorpart2 = alt.Chart(my_ax).mark_line().encode(x='x',y='y')

vectortext_1 = vectorpart2.mark_text( align='left',
baseline='middle', dx=7).encode(text='label')

scale = alt.Scale(domain=['ATP synthases', 'BC', 'CO2 fixation', 'Central
carbohydrate metabolism', 'D', 'Electron accepting reactions', 'Electron
donating reactions', 'Fermentation', 'Flagellar motility in Prokaryota',
```

```
'O', 'OD', 'ODN', 'One-carbon Metabolism', 'Organic acids', 'Protein
translocation across cytoplasmic membrane'], range=['black',
'gray', 'black', 'black', 'red', 'black', 'black', 'black', 'black',
'blue', 'black', 'darkorange', 'black', 'black', 'black'])
dotspart = alt.Chart(my_Tdf).mark_point().encode(x='x', y='y',
color=alt.Color('Treatment', legend=alt.Legend(title='Treatment'),sc
ale=scale), size='Time:Q')

figS6E = dotspart + vectorpart1 + vectortext_1
figS6E
```

Out[29]:

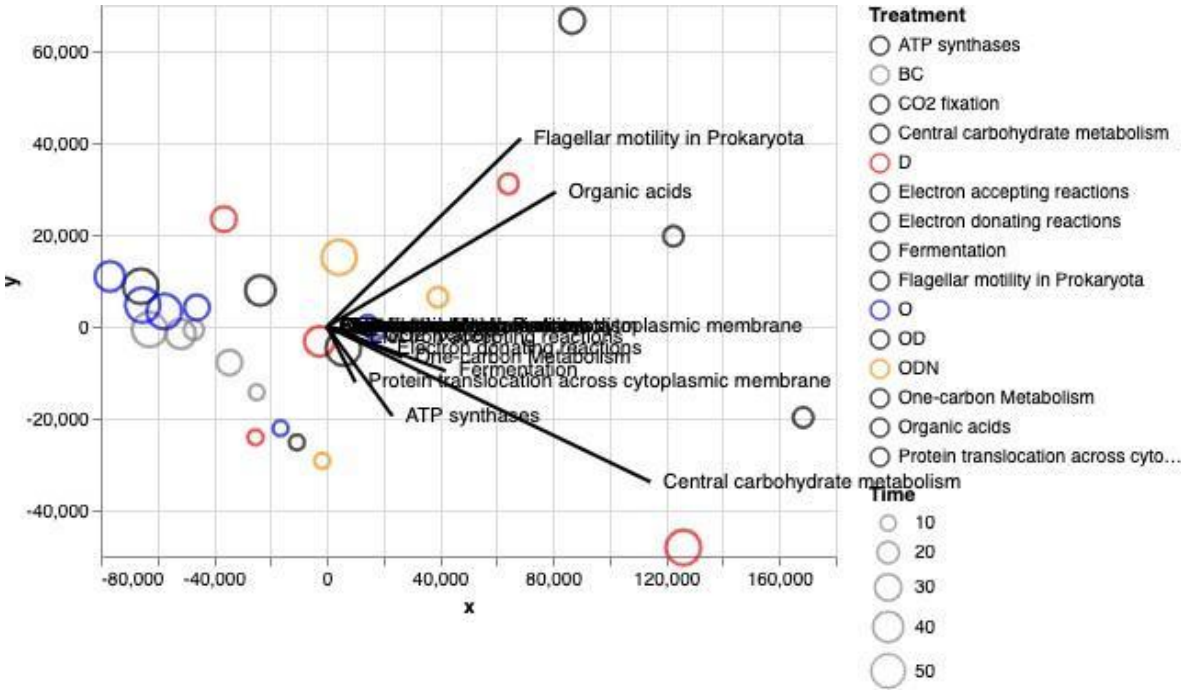

In [30]:

```
#Inspection of detailed table
Location = r'./SK_subsystems_level_3_WITH_REPS.tsv'
input_df = pd.read_csv(Location, sep= '\t', error_bad_lines=False, encod
ing ="ISO-8859-1").reset_index()
input_df = input_df.loc[(input_df['level1'] == 'Motility and Chemotaxis'
) | (input_df['level1'] == 'Carbohydrates') | (input_df['level1'] == 'Me
mbrane Transport') | (input_df['level1'] == 'Respiration') ]

input_df_1 = input_df.loc[:, ['level3', 'BC_0', 'BC_1', 'BC_2', 'BC_3',
'BC_4','D_0', 'D_1', 'D_2', 'D_3', 'D_4', 'ODN_0', 'ODN_1', 'ODN_4', 'OD
_0','OD_1_1', 'OD_1_2', 'OD_2', 'OD_3', 'OD_4_1', 'OD_4_2', 'O_0', 'O_1_
1','O_1_2', 'O_2', 'O_3', 'O_4_1', 'O_4_2']]
input_df_1 = input_df_1.set_index(['level3'])
input_df_1 = input_df_1.T


my_expl, my_important, my_ax, my_Tdf = pca_subsystems(input_df_1, 10)
```

```
vectorpart1 = alt.Chart(my_ax).mark_line().encode(x='x',y='y',
color=alt.Color('label', legend=None) )
vectorpart2 = alt.Chart(my_ax).mark_line().encode(x='x',y='y')
vectortext_1 = vectorpart2.mark_text( align='left',
baseline='middle', dx=7).encode(text='label')

scale = alt.Scale(domain=['Acetyl-CoA_fermentation_to_Butyrate',
'BC', 'D', 'F0F1-type_ATP_synthase', 'Flagellar_motility', 'Flagellum',
'Glyoxylate_bypass', 'Lactate_utilization', 'Methylcitrate_cycle', 'O',
'OD', 'ODN', 'Propionate-CoA_to_Succinate_Module', 'Serine-
glyoxylate_cycle', 'Ton_and_Tol_transport_systems'],
range=['black','gray','red','black','black','black','black','black','black
','blue','black', 'darkorange', 'black','black','black'])

dotspart = alt.Chart(my_Tdf).mark_point().encode(x='x',y='y',
color=alt.Color('Treatment', legend=alt.Legend(title='Treatment'),scale=
scale),size='Time:Q')

figS6F = dotspart + vectorpart1 + vectortext_1
figS6F
```

Out[30]:

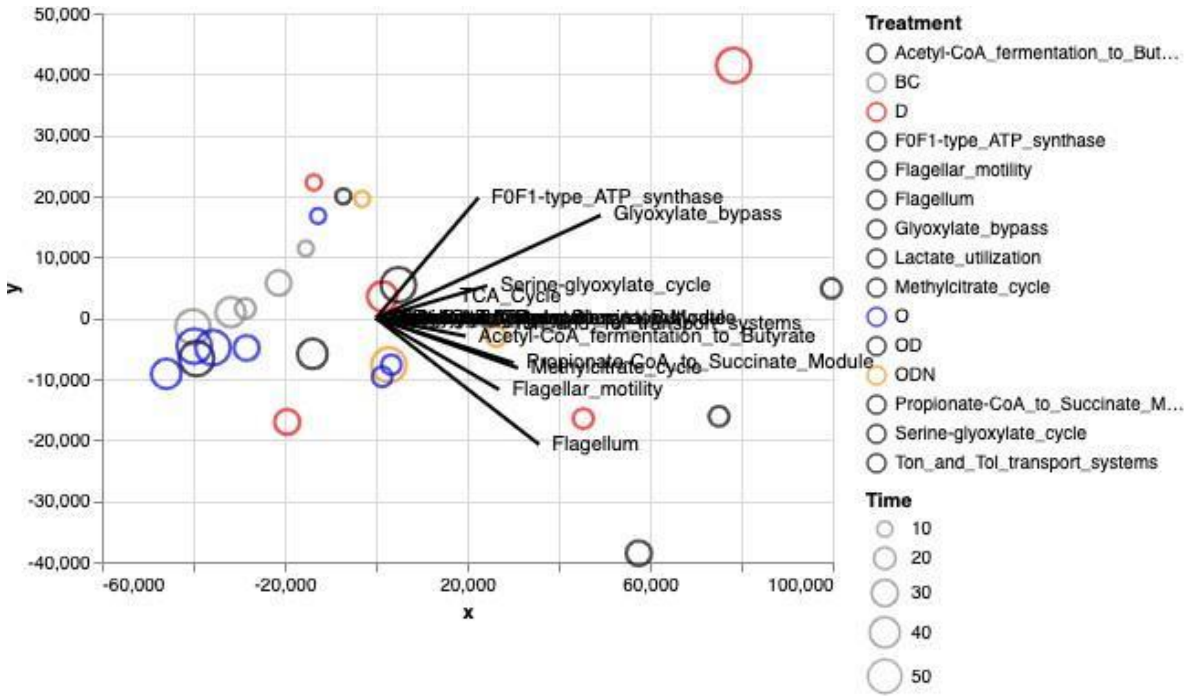

Figure 3B

In [31]:

```
#Inspection of detailed table
Location = r'./SK_subsystems_level_4_WITH_REPS.tsv'
input_df = pd.read_csv(Location, sep= '\t', error_bad_lines=False, encod
ing ="ISO-8859-1").reset_index()
input_df = input_df.loc[(input_df['level1'] == 'Motility and Chemotaxis'
) |(input_df['level1'] == 'Carbohydrates') | (input_df['level1'] == 'Mem
brane Transport') | (input_df['level1'] == 'Respiration') ]
```

```
input_df_1 = input_df.loc[:, ['function', 'BC_0', 'BC_1', 'BC_2', 'BC_3'
, 'BC_4', 'D_0', 'D_1', 'D_2', 'D_3', 'D_4', 'ODN_0', 'ODN_1', 'ODN_4',
'OD_0', 'OD_1_1', 'OD_1_2', 'OD_2', 'OD_3', 'OD_4_1', 'OD_4_2', 'O_0',
'O_1_1', 'O_1_2', 'O_2', 'O_3', 'O_4_1', 'O_4_2']]
input_df_1 = input_df_1.set_index(['function'])
input_df_1 = input_df_1.T
my_expl, my_important, my_ax, my_Tdf = pca_subsystems(input_df_1, 10)
vectorpart1 = alt.Chart(my_ax).mark_line().encode(x='x',y='y',
color=alt.Color('label', legend=None))

vectorpart2 = alt.Chart(my_ax).mark_line().encode(x='x', y='y')
vectortext_1 = vectorpart2.mark_text( align='left',
baseline='middle', dx=7).encode(text='label')

scale = alt.Scale(domain=['2-methylcitrate dehydratase FeS dependent (EC
4.2.1.79)','Acetoacetyl-CoA reductase (EC 1.1.1.36)', 'Acetolactate
synthase large subunit (EC 2.2.1.6)','Acetyl-coenzyme A synthetase (EC
6.2.1.1)', 'Aconitate hydratase 2 (EC 4.2.1.3)', 'BC', 'D', 'Isocitrate
lyase (EC 4.1.3.1)', 'Malate synthase G (EC 2.3.3.9)', 'O','OD',
'ODN','Propionate--CoA ligase (EC 6.2.1.17)', 'Pyruvate formate-lyase (EC
2.3.1.54)', 'TonB-dependent receptor'],
range=['black','black','black','black','black','gray','red','black','black
','blue','black', 'darkorange', 'black','black','black','black'])

dotspart = alt.Chart(my_Tdf).mark_point().encode(x='x', y='y',
color=alt.Color('Treatment',
legend=alt.Legend(title='Treatment'),scale=scale),
shape='Time:N')

fig3B = dotspart + vectorpart1 + vectortext_1
fig3B
```

Out[31]:

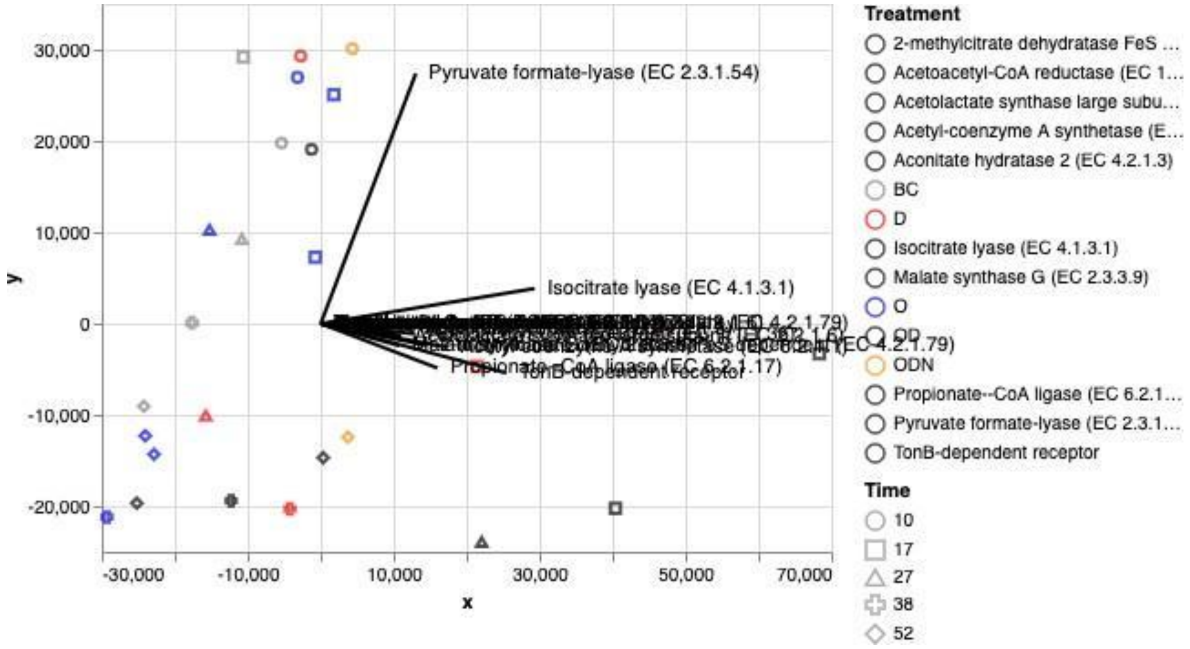

Figure 3A

This clustering analysis was performed in an R notebook (PeñaMontenegro_et_al_2020_R_b-diversity.ipynb). In this section we are going to plot the output file Fig3_pca_samples.csv generated in the R notebook.

In [32]:
```
Location = r'./Fig3_pca_samples.csv'
betasamples_df = pd.read_csv(Location, sep= ',', error_bad_lines=False,
encoding = "ISO-8859-1").reset_index()
betasamples_df.columns = ['col1', 'Tag', 'PC1', 'PC2']
betasamples_df = betasamples_df.drop(['col1'], axis=1)
betasamples_df['Treatment'] = ['Bacterial Control', 'Bacterial Control',
'Bacterial Control','Bacterial Control','Bacterial Control','Dispersant
s', 'Dispersants', 'Dispersants', 'Dispersants', 'Dispersants','WAF','WA
F','WAF','WAF','WAF','WAF','WAF','CEWAF','CEWAF','CEWAF','CEWAF','CEWAF'
,'CEWAF','CEWAF','CEWAF + nutrients', 'CEWAF + nutrients', 'CEWAF + nutr
ients']
betasamples_df['Time'] = [0, 7, 17, 28, 42, 0, 7, 17, 28, 42, 0, 7, 7, 17,
28, 42, 42, 0, 7, 7, 17, 28, 42, 42, 0, 7, 42]

#Let's upload the relative counts for taxonomy
Location = r'./Fig3_pca_species.csv'
betaspecies_df = pd.read_csv(Location, sep= ',', error_bad_lines=False,
encoding = "ISO-8859-1").reset_index()
betaspecies_df.columns = ['col1', 'Tag', 'PC1', 'PC2']
betaspecies_df = betaspecies_df.drop(['col1'], axis=1)

betaspecies_df.PC2 = betaspecies_df.PC2 * 5
```

In [33]:
```

p1 = alt.Chart(betasamples_df).mark_point().encode(x='PC1', y='PC2',
color=alt.Color('Treatment', legend=alt.Legend(title='Treatment'),
scale=alt.Scale(domain=['Bacterial Control', 'Dispersants', 'WAF',
'CEWAF','CEWAF + nutrients'], range=['gray', 'red', 'blue', 'black',
'darkorange'])), shape='Time:N')
p2 = alt.Chart(betaspecies_df).mark_text(align='left',
baseline='middle', dx=7).encode(x='PC1',y='PC2',text='Tag')
fig3A = p1 + p2
fig3A
```

Out[33]:

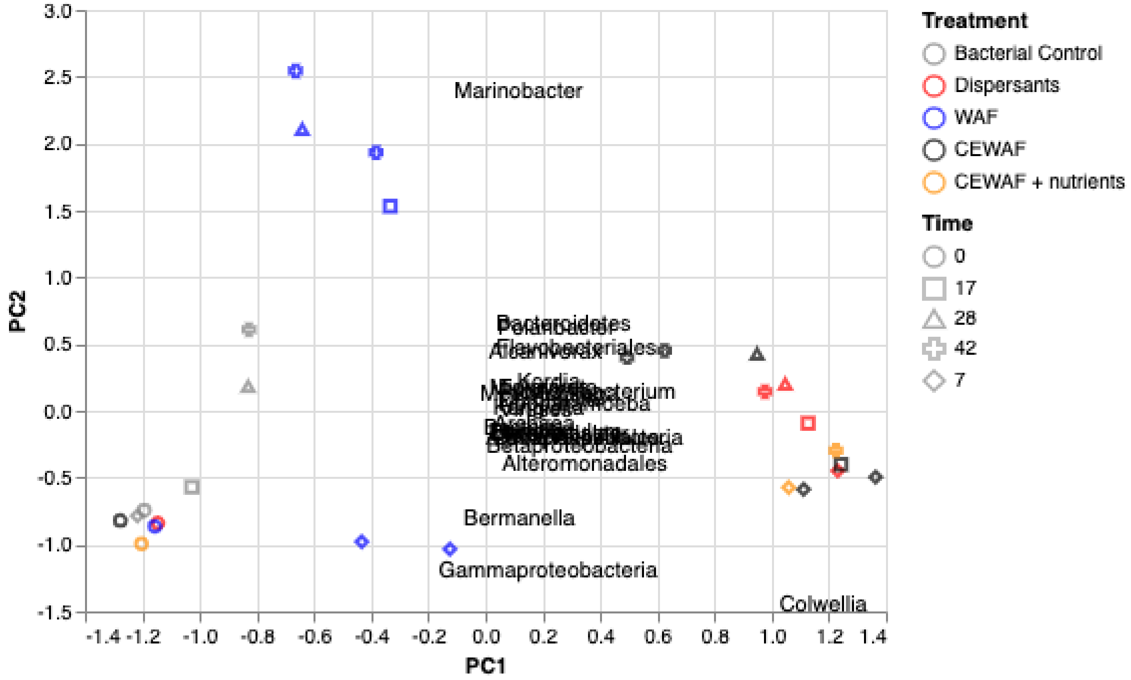

Figure 2

In [34]:

```
Location = r'./16S_VAMPS.tsv'
input_df = pd.read_csv(Location, sep= '\t', error_bad_lines=False
).reset_index()
single_terms = []

for i in input_df.iloc[:,1]:
      terms = i.split(";")
      single_terms.append(terms[-1])

se = pd.Series(single_terms)
input_df['tag'] = se.values
input_df1 = input_df[['tag', 'BC_0_1', 'BC_0_2', 'BC_0_3',
'BC_1_1', 'BC_1_2', 'BC_1_3','BC_2_1', 'BC_2_2', 'BC_2_3',
'BC_3_1', 'BC_3_2', 'BC_3_3', 'BC_4_1', 'BC_4_2', 'BC_4_3',
'D_0_1', 'D_0_2', 'D_0_3', 'D_1_1', 'D_1_2', 'D_1_3',
'D_2_1', 'D_2_2', 'D_2_3', 'D_3_1', 'D_3_2', 'D_3_3',
'D_4_1', 'D_4_2', 'D_4_3', 'O_0_1', 'O_0_2', 'O_0_3',
'O_1_1', 'O_1_2', 'O_1_3', 'O_2_1', 'O_2_2', 'O_2_3',
'O_3_1', 'O_3_2', 'O_3_3', 'O_4_1', 'O_4_2', 'O_4_3',
'OD_0_1', 'OD_0_2', 'OD_0_3', 'OD_1_1', 'OD_1_2', 'OD_1_3',
'OD_2_1', 'OD_2_2', 'OD_2_3', 'OD_3_1', 'OD_3_2', 'OD_3_3',
'OD_4_1', 'OD_4_2', 'OD_4_3', 'ODN_0_1', 'ODN_0_2', 'ODN_0_3', 'ODN_1_1',
'ODN_1_2', 'ODN_1_3', 'ODN_4_1', 'ODN_4_2', 'ODN_4_3']]

input_df1 = input_df1.set_index('tag')
input_df1 = input_df1.T
```

```
treatag = np.arange(1,70)
se = pd.Series(treatag)
input_df1['TreatTag'] = se.values
input_df1 = pd.melt(input_df1, id_vars=['TreatTag'], var_name='Taxonomy'
, value_name='Counts')
input_df1 = input_df1[["Taxonomy", "TreatTag", "Counts"]]
input_df1.to_csv(path_or_buf="16S_format4r.txt", sep="\t", header=False,
index=False)
```

In [35]:

```
#Download and run Perl script
!rm mgrastrawcounts_to_goldDBtagscounts.pl
!rm SK_metaT_v2.db.zip
!rm SK_metaT_v2.db
!wget https://github.com/biotemon/GZ_et_al/raw/master/scripts/mgrastrawc
ounts_to_goldDBtagscounts.pl
!wget https://osf.io/s7u4t/download -O SK_metaT_v2.db.zip
!unzip SK_metaT_v2.db.zip
!cat mgrastrawcounts_to_goldDBtagscounts.pl | sed -e 's/SETDABASEHERE/
\.\/SK\_metaT\_v2\.db/' > mgrastrawcounts_to_goldDBtagscounts_v2.pl
!rm mgrastrawcounts_to_goldDBtagscounts.pl
!mv mgrastrawcounts_to_goldDBtagscounts_v2.pl mgrastrawcounts_to_goldDBt
agscounts.pl
#Run the code
!perl mgrastrawcounts_to_goldDBtagscounts.pl 16S_format4r.txt
```

--2020-09-05 21:45:29-- https://github.com/biotemon/GZ_et_al/raw/maste
r/scripts/mgrastrawcounts_to_goldDBtagscounts.pl
Resolving github.com (github.com)... 140.82.114.4
Connecting to github.com (github.com)|140.82.114.4|:443... connected. HTTP request sent, awaiting
response... 302 Found
Location: https://raw.githubusercontent.com/biotemon/GZ_et_al/master/sc
ripts/mgrastrawcounts_to_goldDBtagscounts.pl [following]
--2020-09-05 21:45:29-- https://raw.githubusercontent.com/biotemon/GZ_
et_al/master/scripts/mgrastrawcounts_to_goldDBtagscounts.pl
Resolving raw.githubusercontent.com (raw.githubusercontent.com)... 199. 232.32.133
Connecting to raw.githubusercontent.com (raw.githubusercontent.com)|19 9.232.32.133|:443...
connected.
HTTP request sent, awaiting response... 200 OK Length: 17972 (18K) [text/plain]
Saving to: 'mgrastrawcounts_to_goldDBtagscounts.pl'

mgrastrawcounts_to_ 100%[===================>] 17.55K --.-KB/s

in 0.01s

2020-09-05 21:45:30 (1.46 MB/s) - ‘mgrastrawcounts_to_goldDBtagscounts. pl’ saved [17972/17972]

--2020-09-05 21:45:30-- https://osf.io/s7u4t/download Resolving osf.io (osf.io)... 35.190.84.173
Connecting to osf.io (osf.io)|35.190.84.173|:443... connected. HTTP request sent, awaiting response... 302 FOUND
Location: https://files.osf.io/v1/resources/fu9bw/providers/dropbox/SK_
metaT_v_may_28_2019.db.zip?action=download&direct&version [following]
--2020-09-05 21:45:31-- https://files.osf.io/v1/resources/fu9bw/provid
ers/dropbox/SK_metaT_v_may_28_2019.db.zip?action=download&direct&versio n
Resolving files.osf.io (files.osf.io)... 35.186.214.196
Connecting to files.osf.io (files.osf.io)|35.186.214.196|:443... connec ted.
HTTP request sent, awaiting response... 200 OK Length: 90160523 (86M) [application/octet-stream]
Saving to: ‘SK_metaT_v2.db.zip’

SK_metaT_v2.db.zip 100%[===================>] 85.98M 9.22MB/s

in 9.7s

2020-09-05 21:45:42 (8.87 MB/s) - ‘SK_metaT_v2.db.zip’ saved [90160523/
90160523]

Archive: SK_metaT_v2.db.zip inflating: SK_metaT_v2.db
Determining taxonomic query terms
99%
[==
=
=======*]Updating taxonomy database and getting tax_ids 19% [==============
]Use of uninitialized value in concatenation (.) or string at mgrastraw counts_to_goldDBtagscounts.pl line 122.
Use of uninitialized value $rkingdom in string ne at mgrastrawcounts_to
_goldDBtagscounts.pl line 175.
Use of uninitialized value $rkingdom in string ne at mgrastrawcounts_to
_goldDBtagscounts.pl line 250.Use of uninitialized value $rsuperkingdom in string ne at mgrastrawcoun ts_to_goldDBtagscounts.pl line 250.
100%
[==
=
========]Processing taxonomyXcounts table

99%
[==
=
=======*]Processing add_manually_terms file

In [36]:

```
#Download R script
!rm plot_CommunityStructure.R
!wget https://raw.githubusercontent.com/biotemon/K2015/master/scripts/pl
ot_CommunityStructure_vSK_v2.R
!mv plot_CommunityStructure_vSK_v2.R plot_CommunityStructure.R
```

--2020-09-05 21:58:04-- https://raw.githubusercontent.com/biotemon/K20
15/master/scripts/plot_CommunityStructure_vSK_v2.R
Resolving raw.githubusercontent.com (raw.githubusercontent.com)... 199. 232.32.133
Connecting to raw.githubusercontent.com (raw.githubusercontent.com)|19 9.232.32.133|:443...
connected.
HTTP request sent, awaiting response... 200 OK Length: 17206 (17K) [text/plain]
Saving to: ‘plot_CommunityStructure_vSK_v2.R’

plot_CommunityStruc 100%[===================>] 16.80K --.-KB/s

in 0.01s

2020-09-05 21:58:04 (1.10 MB/s) - ‘plot_CommunityStructure_vSK_v2.R’ sa ved [17206/17206]

In [37]:

```
#Definition of some important variables
cwd = os.getcwd()

viz_threshold = '4'

#Concatenating names of samples
samples_string = '", "'.join(str(v) for v in treatag)

# Read in the file
with open('plot_CommunityStructure.R', 'r') as file:
      filedata = file.read()
#Setting Desired Order
filedata = filedata.replace('SETDESIREDBARORDERHERE', samples_string)
```

```
#Setting sample names of libraries matching the desired order
filedata = filedata.replace('SETSAMPLENUMBERSHERE', samples_string)

# Setting visualization threshold
filedata = filedata.replace('SETTHRESHOLDHERE', viz_threshold)

# Setting working directory
filedata = filedata.replace('SETWORKINGDIRHERE', cwd)

# Setting taxcounts file
filedata = filedata.replace('SETTAXCOUNTSFILEHERE', '16S_format4r.txt_ta
xonomyXcounts.txt' )

# Setting sample names
filedata = filedata.replace('SETSAMPLENAMESHERE', samples_string)

colortochange = "\n".join([
      'simple_color_vec[2] <- "#512888" #KSU purple',
      'simple_color_vec[3] <- "#D8BFD8" #Thistle',
      'simple_color_vec[4] <- "#cc12c0" #Fucsia',
      'simple_color_vec[5] <- "#9E0142" #Red',
      'simple_color_vec[6] <- "#ff9e9e" #Light red',
      'simple_color_vec[7] <- "#DC143C" #Crimson',
      'simple_color_vec[8] <- "#ff6f00" #Orange',
      'simple_color_vec[9] <- "#FFEF00" #Process Yellow',
      'simple_color_vec[10] <- "#FFD700" #Golden yellow',
      'simple_color_vec[11] <- "#D2691E" #Cocoa brown',
      'simple_color_vec[12] <-"#008000" #Green HTML',
      'simple_color_vec[13] <-"#8db500" #Verde mango biche',
      'simple_color_vec[14] <- "#ACE1AF" #Celadon',
      'simple_color_vec[15] <- "#e6ffe6" #Colwellia',
      'simple_color_vec[16] <- "#4B6F44" #Artichoke Pantone',
      'simple_color_vec[17] <-"#72996c" #Verde gris',
      'simple_color_vec[18] <-"#A8E224" #Green 7',
      'simple_color_vec[19] <- "#008080" #Teal',
      'simple_color_vec[20] <-"#a3cece" #Teal clarito',
      'simple_color_vec[21] <- "#118200" #Verde pasto',
      'simple_color_vec[22] <-"#CAD32E" #Green 11',
      'simple_color_vec[23] <-"#DFFF00" #Chartreuse',
      'simple_color_vec[24] <- "#DADD98" #Green earth'
      'simple_color_vec[25] <- "#00b2ff" #blue',
      'simple_color_vec[26] <- "#97c6bf" #Light grey blue',
      'simple_color_vec[27] <- "#000000" #black',
      'simple_color_vec[28] <- "#c7c5c4" #Grey'])
```

```
filedata = filedata.replace('#SETCOLORSHERE', colortochange)

# Write the file out again
with open('plot_CommunityStructure.R', 'w') as file:
      file.write(filedata)
```

In [38]:

```
#Run the R script
!Rscript plot_CommunityStructure.R
```

Attaching package: ‘dplyr’

The following objects are masked from ‘package:plyr’:

arrange, count, desc, failwith, id, mutate, rename, summarise, summarize

The following objects are masked from ‘package:stats’: filter, lag

The following objects are masked from ‘package:base’: intersect, setdiff, setequal, union

Warning message:
In rowsum.default(t(my_absolute), colnames(my_absolute)) : missing values for 'group'
Warning message:
In rowsum.default(t(my_relative), colnames(my_relative)) : missing values for 'group'
Warning message:
In data.table::melt(simple_absolute_matrix_3, id.vars = "sample_names")
:
The melt generic in data.table has been passed a data.frame and will attempt to redirect to the relevant reshape2 method; please note that r eshape2 is deprecated, and this redirection is now deprecated as well. To continue using melt methods from reshape2 while both libraries are a ttached, e.g. melt.list, you can prepend the namespace like reshape2::m elt(simple_absolute_matrix_3). In the next version, this warning will b ecome an error.
Warning message:
In data.table::melt(simple_relative_matrix_3, id.vars = "sample_names")
:
The melt generic in data.table has been passed a data.frame and will attempt to redirect to the relevant reshape2 method; please note that r eshape2 is deprecated, and this redirection is now deprecated as well. To continue using melt methods from reshape2 while both libraries are a ttached, e.g. melt.list, you can prepend the namespace like reshape2::m elt(simple_relative_matrix_3). In the

next version, this warning will b ecome an error.

null device
1
null device
1
null device
1
null device
1

Now that 16S Data have been summarized at a 4% thereshold of relative abundance, we are going to upload the matrix to generate the 16S vs Metagenome plot.

In [39]:

```
!mv simple_relative_melt.csv 16S_simple_relative_melt.csv
!mv simple_absolute_melt.csv 16S_simple_absolute_melt.csv

Location = r'./16S_simple_relative_melt.csv'
input_df = pd.read_csv(Location, sep= ',', error_bad_lines=False).reset_
index()
input_df = input_df.drop(['index', 'Unnamed: 0'], axis=1)
rrna_16S_df = input_df[input_df.variable != 'Unknown']
Location = r'./fig1_simple_relative_melt.csv'
metagen_df = pd.read_csv(Location, sep= ',', error_bad_lines=False).rese
t_index()

metagen_df['sample_names'] = metagen_df['sample_names'].replace(['bc0',
'bc1', 'bc2', 'bc3', 'bc4', 'd0', 'd1', 'd2', 'd3', 'd4', 'o0', 'o1A',
'o1B','o2', 'o3', 'o4A', 'o4B', 'od0', 'od1A','od1B', 'od2', 'od3',
'od4A', 'od4B', 'odn0', 'odn1', 'odn4'],['bc0', 'bc1', 'bc2', 'bc
3', 'bc4', 'd0', 'd1', 'd2', 'd3', 'd4','o0', 'o1', 'o1','o2', 'o3', 'o4',
'o4', 'od0', 'od1','od1', 'od 2', 'od3','od4', 'od4', 'odn0', 'odn1',
'odn4'])


vec1 = ['o1', 'o4', 'od1', 'od4']
vec2 = metagen_df.variable.unique()

for i in vec1:
      for j in vec2:
            temp = metagen_df[(metagen_df3.variable == j) &
      (metagen_df.samp le_names == i)]
            the_mean = temp.value.mean()
            metagen_df.at[temp.index[0],'value'] = the_mean
            metagen_df = metagen_df[metagen_df.index != temp.index[1]]
```

/usr/local/lib/python3.7/site-packages/ipykernel_launcher.py:3: UserWar ning: Boolean Series key will be reindexed to match DataFrame index.

This is separate from the ipykernel package so we can avoid doing imp orts until

```
In [40]:
tax_se = metagen_df['variable']
tax_se = tax_se.append(rrna_16S_df['variable'])
uniq_tax = tax_se.unique()

#rrna_16S_df
tags = ['bc0', 'bc0', 'bc0', 'bc1', 'bc1', 'bc1', 'bc2', 'bc2', 'bc2',
'bc3', 'bc3', 'bc3', 'bc4', 'bc4', 'bc4', 'd0', 'd0', 'd0',
'd1', 'd1', 'd1', 'd2', 'd2', 'd2', 'd3', 'd3', 'd3', 'd4', 'd4', 'd4',
'o0', 'o0', 'o0', 'o1', 'o1', 'o1', 'o2', 'o2', 'o2', 'o3', 'o3', 'o3',
'o4', 'o4', 'o4', 'od0', 'od0', 'od0', 'od1', 'od1', 'od1', 'od2', 'od2',
'od2', 'od3', 'od3', 'od3', 'od4', 'od4', 'od4', 'odn0', 'odn0',
'odn0','odn1', 'odn1', 'odn1', 'odn4', 'odn4', 'odn4']
tags1 = tags * 30
tags1 = pd.Series(tags1)
rrna_16S_df['tags'] = tags1.values

tag_col = []
tax_col = []
met_col = []
rna_col = []
i = 0
for t in uniq_tax:
      for u in tags:
            if i > 2:
                  i = 0
            tag_col.append(u)
            tax_col.append(t)
            x = metagen_df[(metagen_df.variable == t) &
      (metagen_df.sample_n ames == u)]['value']
            if x.empty:
                  x = '0'
            else:
                  x = x.to_string(header=None, index=None)
            met_col.append(x)
            temp = pd.Series(rrna_16S_df[(rrna_16S_df.variable == t) &
      (rrna_16S_df.tags == u)]['value'])
            temp = temp.reset_index(drop=True)
            if temp.empty:
                  y = 0
            else:
                  y = temp[i]
            rna_col.append(y)
            i = i + 1

In [41]:

d = {'Tag' : pd.Series(tag_col), 'Taxonomy' : pd.Series(tax_col),
'16S_Abundance' : pd.Series(rna_col), 'MG_Abundance' : pd.Series(met_col)}
MG_vs_16s_df = pd.DataFrame(d)
```

```
mg = MG_vs_16s_df['MG_Abundance'].astype(np.float)
rr = MG_vs_16s_df['16S_Abundance'].astype(np.float)
mg = mg + 0.000001
rr = rr + 0.000001
mg_rr_lr = np.log2(mg / rr)
se = pd.Series(mg_rr_lr)
MG_vs_16s_df['MG_16S_Abundance_Ratio']=se.values
```

In [42]:

```
tags2 = ['Control', 'Control', 'Control', 'Control', 'Control', 'Control',
'Control', 'Control', 'Control', 'Control', 'Control', 'Control',
'Control', 'Control', 'Control', 'Dispersant', 'Dispersant', 'Dispersant',
'Dispersant', 'Dispersant', 'Dispersant', 'Dispersant', 'Dispersant',
'Dispersant', 'Dispersant', 'Dispersant', 'Dispersant', 'Dispersant',
'Dispersant', 'Dispersant', 'WAF', 'WAF', 'WAF', 'WAF', 'WAF', 'WAF',
'WAF', 'WAF', 'WAF', 'WAF', 'WAF', 'WAF', 'WAF', 'WAF', 'WAF', 'CEWAF',
'CEWAF', 'CEWAF', 'CEWAF', 'CEWAF', 'CEWAF', 'CEWAF', 'CEWAF', 'CEWAF',
'CEWAF', 'CEWAF', 'CEWAF', 'CEWAF', 'CEWAF', 'CEWAF', 'CEWAF+N',
'CEWAF+N', 'CEWAF+N', 'CEWAF+N', 'CEWAF+N', 'CEWAF+N', 'CEWAF+N',
'CEWAF+N', 'CEWAF+N' ]
tags2 = tags2 * 47

#insert the new column
MG_vs_16s_df.insert(1, "Treatment", tags2, True)
```

In [43]:

```
#For the control
control_subset_df = MG_vs_16s_df[MG_vs_16s_df['Treatment'] == 'Control']
means = control_subset_df.groupby('Taxonomy', as_index=False)['MG_16S_Ab
undance_Ratio'].mean()
means.columns = ['Taxonomy', 'Mean_LR']
control_subset_df = control_subset_df.join(means.set_index('Taxonomy'),
on='Taxonomy')
#For oil
oil_subset_df = MG_vs_16s_df[MG_vs_16s_df['Treatment'] == 'WAF']
means = oil_subset_df.groupby('Taxonomy', as_index=False)['MG_16S_Abunda
nce_Ratio'].mean()
means.columns = ['Taxonomy', 'Mean_LR']
oil_subset_df = oil_subset_df.join(means.set_index('Taxonomy'), on='Taxo
nomy')
#For disp
disp_subset_df = MG_vs_16s_df[MG_vs_16s_df['Treatment'] == 'Dispersant']
means = disp_subset_df.groupby('Taxonomy', as_index=False)['MG_16S_Abund
ance_Ratio'].mean()
means.columns = ['Taxonomy', 'Mean_LR']
disp_subset_df = disp_subset_df.join(means.set_index('Taxonomy'), on='Ta
xonomy')
#For oil disp
cewaf_subset_df = MG_vs_16s_df[MG_vs_16s_df['Treatment'] == 'CEWAF'] means
= cewaf_subset_df.groupby('Taxonomy', as_index=False)['MG_16S_Abun
```

```
dance_Ratio'].mean()
means.columns = ['Taxonomy', 'Mean_LR']
cewaf_subset_df = cewaf_subset_df.join(means.set_index('Taxonomy'), on=
'Taxonomy')
#For oil disp nutr
cewafn_subset_df = MG_vs_16s_df[MG_vs_16s_df['Treatment'] == 'CEWAF+N']
means = cewaf_subset_df.groupby('Taxonomy', as_index=False)['MG_16S_Abun
dance_Ratio'].mean()
means.columns = ['Taxonomy', 'Mean_LR']
cewafn_subset_df = cewafn_subset_df.join(means.set_index('Taxonomy'), on
='Taxonomy')
```

In [44]:

```
frames = [control_subset_df, oil_subset_df, disp_subset_df, cewaf_subset
_df, cewafn_subset_df]
all_ratios_df = pd.concat(frames)

#remove taxa not present in the 16S dataset
all_ratios_df = all_ratios_df[all_ratios_df.Taxonomy != "Microvirus"]
all_ratios_df = all_ratios_df[all_ratios_df.Taxonomy != "Viruses"]
all_ratios_df = all_ratios_df[all_ratios_df.Taxonomy != "Eukaryota"]
all_ratios_df = all_ratios_df[all_ratios_df.Taxonomy != "Neoparamoeba"]
all_ratios_df = all_ratios_df[all_ratios_df.Taxonomy != "Archaea"]

fig2 = alt.Chart(all_ratios_df).mark_point(filled=True).encode(
alt.X('mean(MG_16S_Abundance_Ratio)', scale=alt.Scale(zero=False)), alt.Y(
'Taxonomy',sort=alt.EncodingSortField(field='Mean_LR', op='mean',
order='de scending'),),
alt.Color('Treatment', scale=alt.Scale(domain=['Control', 'Dispersan t',
'WAF', 'CEWAF', 'CEWAF+N'], range=['gray', 'red', 'blue', 'black',
'orange'])),
alt.Size('stdev(MG_16S_Abundance_Ratio)')
).configure_mark(opacity=0.7)

fig2
```

Out[44]:

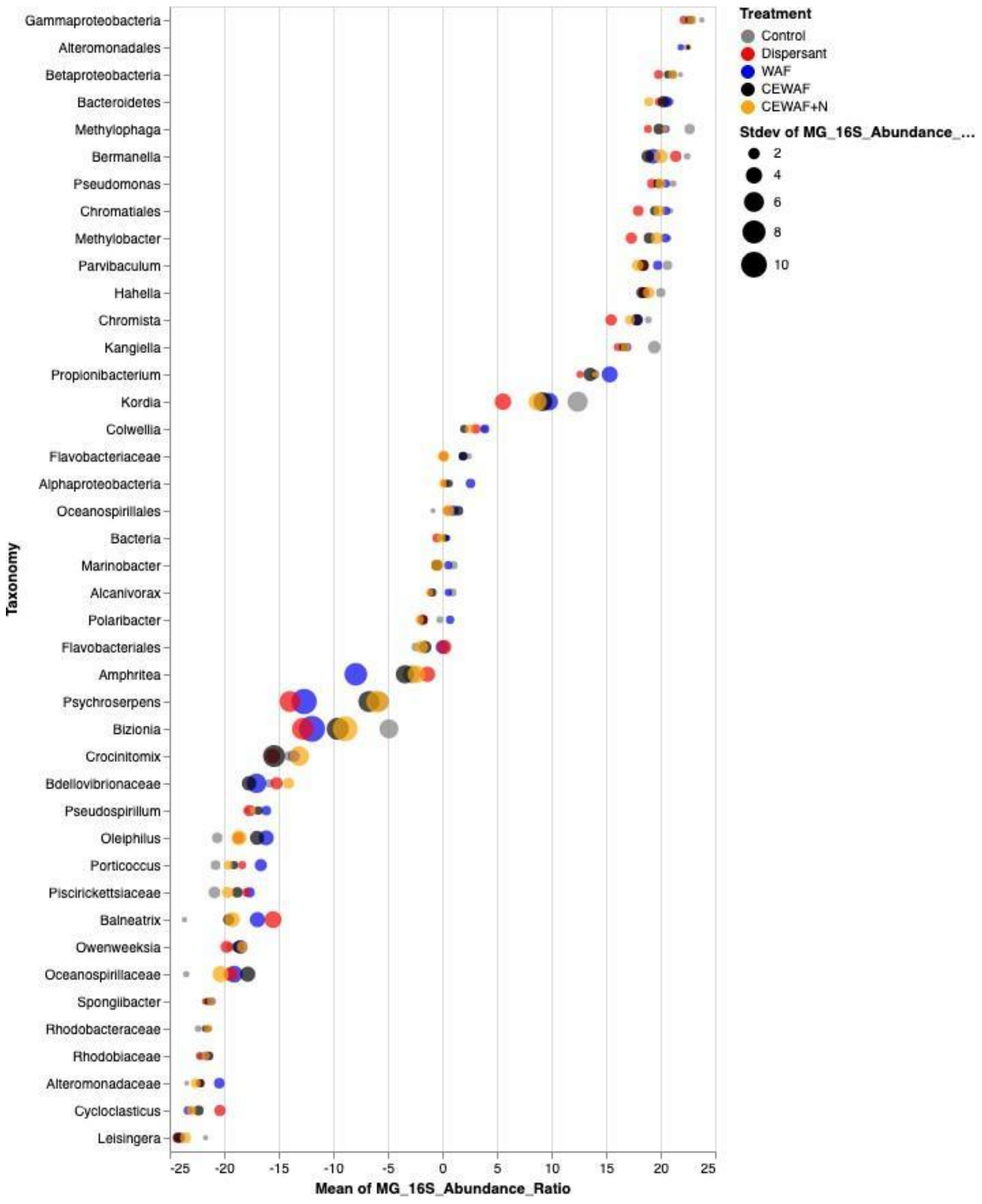

Treatment
Control
Dispersant
WAF
CEWAF
CEWAF+N
Stdev of MG_16S_Abundance_...
2
4
6
8
10
Taxonomy
Gammaproteobacteria
Alteromonadales
Betaproteobacteria
Bacteroidetes
Methylophaga
Bermanella
Pseudomonas
Chromatiales
Methylobacter
Parvibaculum
Hahella
Chromista
Kangiella
Propionibacterium
Kordia
Colwellia
Flavobacteriaceae
Alphaproteobacteria
Oceanospirillales
Bacteria
Marinobacter
Alcanivorax
Polaribacter
Flavobacteriales
Amphritea
Psychroserpens
Bizionia
Crocinitomix
Bdellovibrionaceae
Pseudospirillum
Oleiphilus
Porticoccus
Piscirickettsiaceae
Balneatrix
Owenweeksia
Oceanospirillaceae
Spongiibacter
Rhodobacteraceae
Rhodobiaceae
Alteromonadaceae
Cycloclasticus
Leisingera
-25
-20
-15
-10
-5
0
5
10
15
20
25
Mean of MG_16S_Abundance_Ratio

Supplementary Figures 5 and 6

In [45]:

```
#Functions are defined here.

def unique_list(l): x = []
      for a in l:
            if a not in x:
                  x.append(a)
      return(x)


def prepare_df_for_fig10(the_dataframe):
      """
      This function will return a reformatted df for figure 10 format
      """
      n = the_dataframe['level_0'].size
      treatment = []
      timevec = []
      for i in np.arange(0,n):
            tag = the_dataframe['level_0'].iloc[i]
            tag_vec = tag.split("_")
            treatment.append(tag_vec[0])
            timevec.append(tag_vec[1])
      val_old = ['0', '1', '2', '3', '4']
      val_new = [0, 7, 17, 28, 42]
      d = dict(zip(val_old, val_new))
      timevec_new = [d.get(e, e) for e in timevec]
      se1 = pd.Series(timevec_new)
      se2 = pd.Series(treatment)

      the_dataframe['Time'] = se1.values
      the_dataframe['Treatment'] = se2.values

      the_dataframe1 = the_dataframe[['level_0', 'level_1', 'dataset', 'a
bundance']]
      the_dataframe2 = the_dataframe1.pivot_table(index=['level_1','datase
t'], columns='level_0')
      the_dataframe2 = the_dataframe2.reset_index()


      the_dataframe2 = the_dataframe2.set_index('dataset')
      level1_metabolism = the_dataframe2.pop("level_1")
      the_dataframe2 = the_dataframe2.xs('abundance', axis=1, drop_level=T
rue)

      #Average replicates
      the_dataframe2['4_OD_1'] = the_dataframe2[['4_OD_1_1', '4_OD_1_2']].
mean(axis=1)
      the_dataframe2 = the_dataframe2.drop(['4_OD_1_1', '4_OD_1_2'],
axis=1)
      the_dataframe2['4_OD_4'] = the_dataframe2[['4_OD_4_1', '4_OD_4_2']].
mean(axis=1)
```

```
    the_dataframe2 = the_dataframe2.drop(['4_OD_4_1', '4_OD_4_2'], axis=
    1)
    the_dataframe2['3_O_1'] = the_dataframe2[['3_O_1_1', '3_O_1_2']].mea
    n(axis=1)
    the_dataframe2 = the_dataframe2.drop(['3_O_1_1', '3_O_1_2'], axis=1)
    the_dataframe2['3_O_4'] = the_dataframe2[['3_O_4_1', '3_O_4_2']].mea
    n(axis=1)
    the_dataframe2 = the_dataframe2.drop(['3_O_4_1', '3_O_4_2'], axis=1)
    sorted_col_names = sorted(list(the_dataframe2.columns.values))
    the_dataframe2 = the_dataframe2[sorted_col_names]

    return(the_dataframe2)

def fix_names_in_df(the_dataframe):
    #Fixing two issues to make it compatible with altair in columns
    data set and level_0
    #Remove the first six characters and last word of the pathway
    name #Example:
    #Input: 04930 Type II diabetes mellitus
    [PATH:ko04930] #Desired output: Type II diabetes
    mellitus
    for index, row in the_dataframe.iterrows():
        old_name =
        the_dataframe.at[index,'dataset']
        short_name = old_name[6:]
        short_name = short_name.rsplit(' ', 1)[0]
        the_dataframe.at[index,'dataset'] = short_name
        sample_name = the_dataframe.at[index,'level_0']
        sample_name =
        re.sub("^BC","1_BC",sample_name.rstrip()) sample_name
        = re.sub("^D","2_D",sample_name.rstrip())
        sample_name =
        re.sub("^ODN","5_ODN",sample_name.rstrip())
        sample_name =
        re.sub("^OD","4_OD",sample_name.rstrip()) sample_name
        = re.sub("^O","3_O",sample_name.rstrip())
        the_dataframe.at[index,'level_0'] = sample_name
    return(the_dataframe)


#Let's remove those pathways very rich in zeros across treatment points
#pathways expressed in 80% or more of the samples the threshold is 20

def filter_zero_rows(the_dataframe, max_zeros_t):
    for index, row in the_dataframe.iterrows(): count = 0
        for i in row:
            if(i == 0):
                count = count + 1
        perc_of_zeros = (count * 100)/the_dataframe.shape[1]
        if(perc_of_zeros > max_zeros_t):
            the_dataframe = the_dataframe.drop(index)
    return(the_dataframe)
```

```
def f_lognormal(x, a, b, c):
    return a * (1/np.add(x,0.000000000001)) * (np.exp(-(np.log(np.add(x,
    0.000000000001))-c)**2/b))

def get_lognormal_r_and_x0(X,data):
    fit_log = curve_fit(f_lognormal,X,data,maxfev=100000000)
    if(fit_log):
        aa = fit_log[0][0]
        bb = fit_log[0][1]
        cc = fit_log[0][2]
        fit = lambda t : aa * (1/np.add(t,0.000000000001)) * (np.exp(-
        (np.lo g(np.add(t,0.000000000001))-cc)**2/bb))
        the_r = r2_score(data,fit(X))
    return(the_r,cc)

def get_parabolic_r_x0_and_a(x1,y):
    z = np.polyfit(x1, y, 2)
    p = np.poly1d(z)
    y_pred = p(x1)
    the_a = z[0] #a
    the_x0 = -1 * z[1] / (2 * z[0]) #the_x0
    the_r = r2_score(y, y_pred)
    return(the_r, the_x0, the_a)

def get_linear_r_x0_and_m(x1,y):
    z = np.polyfit(x1, y, 1)
    p = np.poly1d(z)
    y_pred = p(x1)
    the_m = z[0] #m
    the_x0 = z[1] #the_x0
    the_r = r2_score(y, y_pred)
    return(the_r, the_x0, the_m)

def enrich_score_and_remove_low_mean(the_dataframe,min_r,min_m):

    #Adding model scores columns
    the_dataframe = the_dataframe.fillna(0)
    c1='Model_BC'
    c2='Model_D'
    c3='Model_O'
    c4='Model_OD'
    c5='Model_ODN'
    the_dataframe[c1] = float(0)
    the_dataframe[c2] = float(0)
    the_dataframe[c3] = float(0)
    the_dataframe[c4] = float(0)
    the_dataframe[c5] = float(0)

    for index, row in the_dataframe.iterrows():
    x1 = [0, 7, 17, 28, 42]
    x3 = [0, 7, 42]

    #bc
    y = row[0:5,]
```

```
xx = x1
the_col = c1

r_lin, x0_lin, the_m = get_linear_r_x0_and_m(xx,y)
if(the_m >= min_m and r_lin > min_r):
      the_dataframe.at[index,the_col] = r_lin
if(the_m <= -min_m and r_lin > min_r):
      the_dataframe.at[index,the_col] = r_lin + 2
if(the_m > -min_m and the_m < min_m and r_lin > min_r):
      the_dataframe.at[index,the_col] = r_lin + 4
try:
      r_log, xpeak = get_lognormal_r_and_x0(xx,y)
      if(xpeak>0 and xpeak<=21 and r_log>min_r and r_log>r_lin):
            the_dataframe.at[index,the_col] = r_log + 6
      r_logR, xpeakR = get_lognormal_r_and_x0(xx,y.iloc[::-1])
      if(xpeakR>0 and xpeakR<=21 and r_logR>min_r and
r_logR>r_logand r_logR>r_lin):
            the_dataframe.at[index,the_col] = r_logR + 8
except(RuntimeError):
      pass

r_par, xminp, a_par = get_parabolic_r_x0_and_a(xx,y)
if(xminp>3 and xminp<39 and a_par>0 and r_par>min_r and r_par>r_ log
and r_par>r_logR and r_par>r_lin):
      the_dataframe.at[index,the_col] = r_par + 10

#d
y = row[5:10,]
xx = x1
the_col = c2
r_lin, x0_lin, the_m = get_linear_r_x0_and_m(xx,y)
if(the_m >= min_m and r_lin > min_r):
      the_dataframe.at[index,the_col] = r_lin
if(the_m <= -min_m and r_lin > min_r):
      the_dataframe.at[index,the_col] = r_lin + 2
if(the_m > -min_m and the_m < min_m and r_lin > min_r):
      the_dataframe.at[index,the_col] = r_lin + 4

try:
      r_log, xpeak = get_lognormal_r_and_x0(xx,y)
      if(xpeak>0 and xpeak<=21 and r_log>min_r and r_log>r_lin):
            the_dataframe.at[index,the_col] = r_log + 6
      r_logR, xpeakR = get_lognormal_r_and_x0(xx,y.iloc[::-1])
      if(xpeakR>0 and xpeakR<=21 and r_logR>min_r and
      r_logR>r_logand r_logR>r_lin):
            the_dataframe.at[index,the_col] = r_logR + 8
except:
      pass

r_par, xminp, a_par = get_parabolic_r_x0_and_a(xx,y)
if(xminp>3 and xminp<39 and a_par>0 and r_par>min_r and r_par>r_ log
and r_par>r_logR and r_par>r_lin):
      the_dataframe.at[index,the_col] = r_par + 10
```

```
#o
y = row[10:15,]
xx = x1
the_col = c3
r_lin, x0_lin, the_m = get_linear_r_x0_and_m(xx,y)
if(the_m >= min_m and r_lin > min_r):
      the_dataframe.at[index,the_col] = r_lin
if(the_m <= -min_m and r_lin > min_r):
      the_dataframe.at[index,the_col] = r_lin + 2
if(the_m > -min_m and the_m < min_m and r_lin > min_r):
      the_dataframe.at[index,the_col] = r_lin + 4

try:
r_log, xpeak = get_lognormal_r_and_x0(xx,y)
if(xpeak>0 and xpeak<=21 and r_log>min_r and r_log>r_lin):
      the_dataframe.at[index,the_col] = r_log + 6
r_logR, xpeakR = get_lognormal_r_and_x0(xx,y.iloc[::-1])
if(xpeakR>0 and xpeakR<=21 and r_logR>min_r and r_logR>r_log and
r_logR>r_lin):
      the_dataframe.at[index,the_col] = r_logR + 8

except:
      pass

r_par, xminp, a_par = get_parabolic_r_x0_and_a(xx,y)
if(xminp>3 and xminp<39 and a_par>0 and r_par>min_r and r_par>r_ log
and r_par>r_logR and r_par>r_lin):
      the_dataframe.at[index,the_col] = r_par + 10

#od
y = row[15:20,]
xx = x1
the_col = c4
r_lin, x0_lin, the_m = get_linear_r_x0_and_m(xx,y)
if(the_m >= min_m and r_lin > min_r):
      the_dataframe.at[index,the_col] = r_lin
if(the_m <= -min_m and r_lin > min_r):
      the_dataframe.at[index,the_col] = r_lin + 2
if(the_m > -min_m and the_m < min_m and r_lin > min_r):
      the_dataframe.at[index,the_col] = r_lin + 4

try:

r_log, xpeak = get_lognormal_r_and_x0(xx,y)
if(xpeak>0 and xpeak<=21 and r_log>min_r and r_log>r_lin):
      the_dataframe.at[index,the_col] = r_log + 6
r_logR, xpeakR = get_lognormal_r_and_x0(xx,y.iloc[::-1])
if(xpeakR>0 and xpeakR<=21 and r_logR>min_r and r_logR>r_log and
r_logR>r_lin):
      the_dataframe.at[index,the_col] = r_logR + 8
except:
      pass
r_par, xminp, a_par = get_parabolic_r_x0_and_a(xx,y)
if(xminp>3 and xminp<39 and a_par>0 and r_par>min_r and r_par>r_ log
```

```
    and r_par>r_logR and r_par>r_lin):
          the_dataframe.at[index,the_col] = r_par + 10

    #odn
    y = row[20:23,]
    xx = x3
    the_col = c5
    r_lin, x0_lin, the_m = get_linear_r_x0_and_m(xx,y)
    if(the_m >= min_m and r_lin > min_r):
          the_dataframe.at[index,the_col] = r_lin
    if(the_m <= -min_m and r_lin > min_r):
          the_dataframe.at[index,the_col] = r_lin + 2
    if(the_m > -min_m and the_m < min_m and r_lin > min_r):
          the_dataframe.at[index,the_col] = r_lin + 4

    try:
    r_log, xpeak = get_lognormal_r_and_x0(xx,y)
    if(xpeak>0 and xpeak<=21 and r_log>min_r and r_log>r_lin):
          the_dataframe.at[index,the_col] = r_log + 6
    r_logR, xpeakR = get_lognormal_r_and_x0(xx,y.iloc[::-1])
    if(xpeakR>0 and xpeakR<=21 and r_logR>min_r and r_logR>r_log and
    r_logR>r_lin):
          the_dataframe.at[index,the_col] = r_logR + 8
    except:
          pass

    r_par, xminp, a_par = get_parabolic_r_x0_and_a(xx,y)
    if(xminp>3 and xminp<39 and a_par>0 and r_par>min_r and r_par>r_ log
    and r_par>r_logR and r_par>r_lin):
          the_dataframe.at[index,the_col] = r_par + 10

    return(the_dataframe)


def normalize_cluster_and_reformat(the_dataframe, first_dataframe):
    #Slice dataframe by the columns of samples
    heat_data = the_dataframe.iloc[:,0:23]
    #Slice dataframe by the columns of tags
    enrich_data = the_dataframe.iloc[:,23:28]
    x = heat_data.values #returns a numpy array
    max_row = np.amax(x, axis=1) #max by row
    x_scaled = x / max_row[:,None]
    x_norm = pd.DataFrame(x_scaled)
    the_colnames = heat_data.columns.values.tolist()
    the_indexes = list(heat_data.index)
    x_norm.columns=the_colnames
    x_norm.index=the_indexes
    dict_temp1 = first_dataframe[first_dataframe['dataset'].isin(enrich_
    data.index.values.tolist())][['dataset',
    'level_2']].sort_values(by=['le vel_2']).drop_duplicates()
    names_in_order = dict_temp1['dataset'].tolist()
    level2_vec_temp = []
    module_vec_temp = []
    for i in unique_list(names_in_order):
```

```
        a = dict_temp1[dict_temp1['dataset'] == i]['level_2'].iloc[0]
        level2_vec_temp.append(a)
        module_vec_temp.append(i)
d = {'dataset': module_vec_temp, 'level_2': level2_vec_temp}
dict_temp2 = pd.DataFrame(data=d)
names_in_order = dict_temp2['dataset'].tolist()
#Rename dataframe based on the name ordered after clustering
j=1
for i in names_in_order:
        xxx = format(j, "03d")
        new_index_name = str(xxx) + "_" + i
        j=j+1
        x_norm = x_norm.rename(index={i: new_index_name})
#change to longformat for altair
x_norm = x_norm.reset_index()
x_norm_longformat = pd.melt(x_norm, id_vars=['index'], var_name='Tre
atment', value_name='Reads')
#Data for enrichment score reformat
enrich_data_1 = enrich_data.loc[names_in_order,:]
#Rename index dataframe based on the name ordered after clustering
j=1
for i in names_in_order:
        xxx = format(j, "03d")
        new_index_name = str(xxx) + "_" + i
        j=j+1
        enrich_data_1 = enrich_data_1.rename(index={i:
        new_index_name})
#del enrich_data_1.columns.name
enrich_data_1 = enrich_data_1.reset_index()
enrich_data_1_longformat = pd.melt(enrich_data_1, id_vars=['dataset'
], var_name='Model', value_name='R2')
enrich_data_1_longformat = enrich_data_1_longformat.rename(columns={
"dataset": "index", "Model": "Model", "R2" : "R2"})

lev_2_tag = enrich_data_1


lev_2_tag = lev_2_tag.drop(lev_2_tag.iloc[:, 1:15], axis=1)
lev_2_tag = lev_2_tag.rename(columns={"dataset": "z_level2"})
j = 0

for i in names_in_order:
        the_level_2 =
        first_dataframe[first_dataframe['dataset']==i].lev
        el_2.iloc[0]
        lev_2_tag.iloc[j,0] = the_level_2
        j = j+1
lev_2_tag['value'] = 1
lev_2_tag.reset_index(level=0, inplace=True)

return(x_norm_longformat, names_in_order, enrich_data_1_longformat,
lev_2_tag)
```

In [46]:
```
#Here we collect some info to normalize against library size
Location = r'./sk_summary_data.csv'
sk_df = pd.read_csv(Location)
#Subset columns for QC figure
qc_df=sk_df.loc[:,['sample_id','passed_qc','failed_qc']]
#Library sizes for normalization purposes of other analysis
l_size = qc_df
l_size['Library Size']=l_size['passed_qc'] + l_size['failed_qc']
l_size = l_size.drop(['passed_qc','failed_qc'], axis=1)
the_lib_size = l_size['Library Size']

# Normalize by library size - a prologue
Location = r'./SK_KO_level_3_WITH_REPS.tsv'
input_df = pd.read_csv(Location, sep= '\t', error_bad_lines=False).reset
_index()
input_df2 = input_df
input_df2A = fix_names_in_df(input_df2)
input_df2A = input_df2A[input_df2A.level_1 != 'Human Diseases']
input_df2A = input_df2A[input_df2A.level_1 != 'Organismal Systems']
input_df2A = input_df2A[input_df2A.level_2 != 'Metabolism of terpenoids
and polyketides']
input_df2A = input_df2A[input_df2A.dataset != 'Plant hormone signal tran
sduction']
input_df2A = input_df2A[input_df2A.dataset != 'HIF-1 signaling pathway']
input_df2A = input_df2A[input_df2A.dataset != 'Spliceosome']
input_df2A = input_df2A[input_df2A.dataset != 'Ribosome biogenesis in eu
karyotes']
input_df2A = input_df2A[input_df2A.dataset != 'Peroxisome']
input_df2A = input_df2A[input_df2A.dataset != 'Phagosome']
input_df2A = input_df2A[input_df2A.dataset != 'Lysosome']
input_df2A = input_df2A[input_df2A.dataset != 'Flavonoid biosynthesis']
input_df2A = input_df2A[input_df2A.dataset != 'Stilbenoid, diarylheptano
id and gingerol biosynthesis']
input_df2A = input_df2A[input_df2A.dataset != 'Phenylpropanoid biosynthe
sis']
input_df2A = input_df2A[input_df2A.dataset != 'Tropane, piperidine and p
yridine alkaloid biosynthesis']
input_df2A = input_df2A[input_df2A.dataset != 'Tight junction']
input_df2A = input_df2A[input_df2A.dataset != 'Gap junction']
input_df2A = input_df2A[input_df2A.dataset != 'Cell cycle - Caulobacter'
]
input_df2A = input_df2A[input_df2A.dataset != 'Cell cycle - yeast']
input_df2A = input_df2A[input_df2A.dataset != 'Cell cycle']
input_df2A = input_df2A[input_df2A.dataset != 'p53 signaling pathway']
input_df2A = input_df2A[input_df2A.dataset != 'Protein processing in end
oplasmic reticulum']

#Normalize by library size
input_df3 = prepare_df_for_fig10(input_df2A)
input_df3_sl = input_df3
j=0

for column in input_df3:
```

```
      input_df3_sl[str(j)] = np.true_divide(input_df3_sl[column], the_lib_
      size[j]) * np.mean(the_lib_size)
      input_df3_sl = input_df3_sl.drop([column], axis=1)
      input_df3_sl[column] = input_df3_sl[str(j)]
      input_df3_sl = input_df3_sl.drop([str(j)], axis=1)
      j=j+1

#Rows with more than 80% of cells with zeros are not considered for the
analysis.
#To detect core pathways, reduce this threshold.
input_df3_sl = filter_zero_rows(input_df3_sl,20)


#Explanation of enrich_score_and_remove_low_mean
#Removal of very low abundance pathways
#1. dataframe
#2. low abundance threshold: We are not considering those rows with an
average of 0 reads per pathway
#3. Absolute distance to Enrichment_score = 0.88. We don't consider those
pathways in between -0.88 and 0.88
#input_df5 = enrich_score_and_remove_low_mean(input_df3_sl,0,0.88)


In [47]:
output = normalize_cluster_and_reformat(input_df5, input_df2)
heat_norm_longformat = output[0]
names_in_order = output[1] enrich_longformat = output[2]
level2_longformat = output[3]

enrich_longformat2 = enrich_longformat
# Add maximum value of the transcript to include the transcript signal i
ntensity in context
bc_max = input_df5.iloc[:,0:5].max(axis=1)
d_max = input_df5.iloc[:,5:10].max(axis=1)
o_max = input_df5.iloc[:,10:15].max(axis=1)
od_max = input_df5.iloc[:,15:20].max(axis=1)
odn_max = input_df5.iloc[:,20:23].max(axis=1)

bc_untag = pd.Series(['Model_BC'])
bc_tags = bc_untag.repeat(bc_max.shape[0])
d_untag = pd.Series(['Model_D'])
d_tags = d_untag.repeat(d_max.shape[0])
o_untag = pd.Series(['Model_O'])
o_tags = o_untag.repeat(o_max.shape[0])
od_untag = pd.Series(['Model_OD'])
od_tags = od_untag.repeat(od_max.shape[0])
odn_untag = pd.Series(['Model_ODN'])
odn_tags = odn_untag.repeat(odn_max.shape[0])

#type(bc_max)
mx_vals = pd.concat([bc_max, d_max, o_max, od_max, odn_max])

mx_frame = { 'max_values': mx_vals} mx_df = pd.DataFrame(mx_frame)
mx_df.reset_index(level=0, inplace=True)
```

```
mx_tags = pd.concat([bc_tags, d_tags, o_tags, od_tags, odn_tags])
mx_tags = mx_tags.reset_index()
mx_tags = mx_tags.drop(['index'], axis=1)
mx_df['Model'] = mx_tags

enrich_longformat2["MaxTS"] = ""
for index, row in enrich_longformat2.iterrows():
      text = enrich_longformat2.at[index,'index']
      k = text.split('_')
      k1 = mx_df[mx_df.dataset == k[1]]
      k2 = k1[k1.Model == enrich_longformat.at[index,'Model']]
      k3 = np.log(k2.max_values)
      k4 = k3.to_string(header=None, index=None)
      enrich_longformat2.at[index,'MaxTS'] = k4

enrich_tags_plot = alt.Chart(enrich_longformat2).mark_circle().encode(
x='Model',
y=alt.Y('index'), size='MaxTS:Q',
color = alt.Color('R2', scale=alt.Scale(domain=[0,1,1.01,1.99,2,3,3.
01,3.99,4,5,5.01,5.99,6,7,7.01,7.99,8,9,9.01,9.99,10,11],
range=['white','red','white','white','white','blue','white','white','white
','aqua','white','white','white','orange','white','white','white','green',
'white','white','white','black']),legend=alt. Legend(title='R2')))


level2_plot = alt.Chart(level2_longformat).mark_rect().encode(
x='value:N',
y=alt.Y('index:N', axis=None),
color = alt.Color('z_level2', scale=alt.Scale(domain=['Cell communic
ation', 'Cell growth and death', 'Cell motility', 'Transport and catabol
ism', 'Membrane transport', 'Signal transduction',
'Signaling molecules and interaction', 'Folding, sorting and degradation',
'Replication and repair', 'Transcription', 'Translation',
'Amino acid metabolism', 'Biosynthesis of other secondary metabolites',
'Carbohydrate
metabolism', 'Energy metabolism', 'Glycan biosynthesis and metabolism',
'Lipid metabolism', 'Metabolism of cofactors and vitamins', 'Metabolism of
other amino acids', 'Metabolism of terpenoids and polyketides',
'Nucleotide metabolism', 'Xenobiotics biodegradation and metabolism'],
range=['black', 'silver', 'gray', 'purple', 'fuchsia', 'green', 'lime',
'olive', 'yellow', 'blue', 'teal', 'aqua', 'orange', 'aquamarine',
'coral', 'cornflowerblue', 'darkgoldenrod', 'darkmagenta',
'darkolivegreen', 'deeppink', 'deepskyblue', 'pink']),legend=alt.Legend(
title='Pathway Family')))
figS7 = enrich_tags_plot | level2_plot
figS7
```

Out[47]:

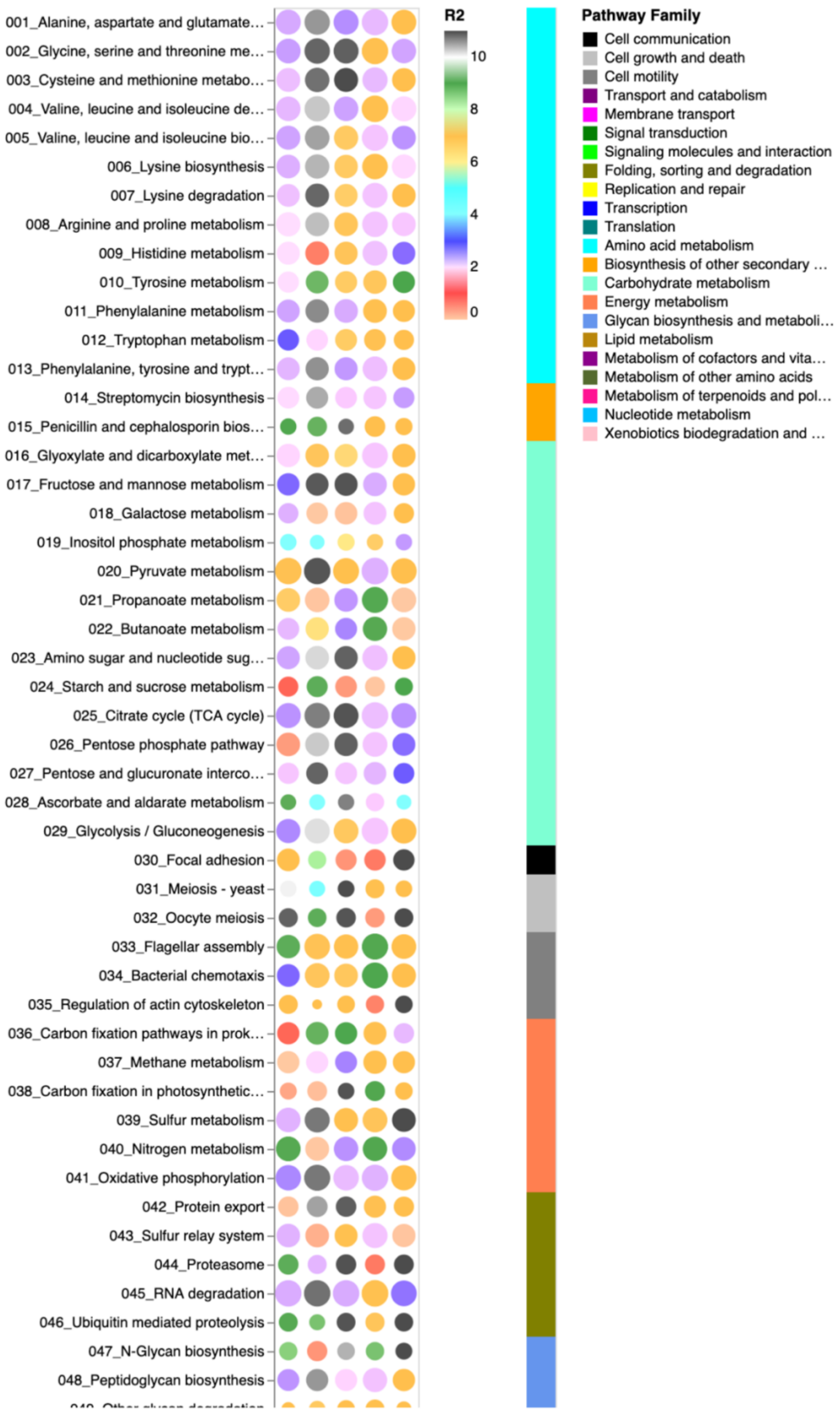
001_Alanine, aspartate and glutamate…
002_Glycine, serine and threonine me…
003_Cysteine and methionine metabo…
004_Valine, leucine and isoleucine de…
005_Valine, leucine and isoleucine bio…
006_Lysine biosynthesis
007_Lysine degradation
008_Arginine and proline metabolism
009_Histidine metabolism
010_Tyrosine metabolism
011_Phenylalanine metabolism
012_Tryptophan metabolism
013_Phenylalanine, tyrosine and trypt…
014_Streptomycin biosynthesis
015_Penicillin and cephalosporin bios…
016_Glyoxylate and dicarboxylate met…
017_Fructose and mannose metabolism
018_Galactose metabolism
019_Inositol phosphate metabolism
020_Pyruvate metabolism
021_Propanoate metabolism
022_Butanoate metabolism
023_Amino sugar and nucleotide sug…
024_Starch and sucrose metabolism
025_Citrate cycle (TCA cycle)
026_Pentose phosphate pathway
027_Pentose and glucuronate interco…
028_Ascorbate and aldarate metabolism
029_Glycolysis / Gluconeogenesis
030_Focal adhesion
031_Meiosis - yeast
032_Oocyte meiosis
033_Flagellar assembly
034_Bacterial chemotaxis
035_Regulation of actin cytoskeleton
036_Carbon fixation pathways in prok…
037_Methane metabolism
038_Carbon fixation in photosynthetic…
039_Sulfur metabolism
040_Nitrogen metabolism
041_Oxidative phosphorylation
042_Protein export
043_Sulfur relay system
044_Proteasome
045_RNA degradation
046_Ubiquitin mediated proteolysis
047_N-Glycan biosynthesis
048_Peptidoglycan biosynthesis
R2
10
8
6
4
2
0
Pathway Family
Cell communication
Cell growth and death
Cell motility
Transport and catabolism
Membrane transport
Signal transduction
Signaling molecules and interaction
Folding, sorting and degradation
Replication and repair
Transcription
Translation
Amino acid metabolism
Biosynthesis of other secondary …
Carbohydrate metabolism
Energy metabolism
Glycan biosynthesis and metaboli…
Lipid metabolism
Metabolism of cofactors and vita…
Metabolism of other amino acids
Metabolism of terpenoids and pol…
Nucleotide metabolism
Xenobiotics biodegradation and …

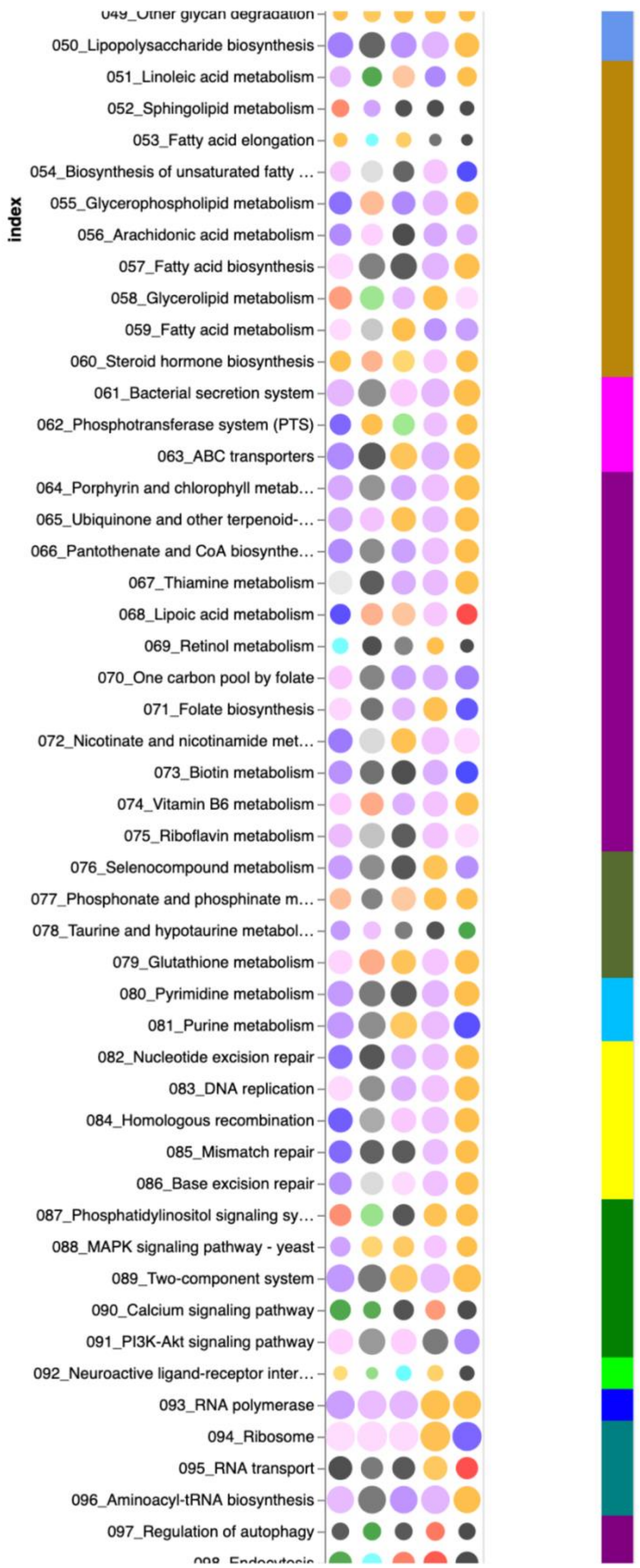
index
049_Other glycan degradation
050_Lipopolysaccharide biosynthesis
051_Linoleic acid metabolism
052_Sphingolipid metabolism
053_Fatty acid elongation
054_Biosynthesis of unsaturated fatty …
055_Glycerophospholipid metabolism
056_Arachidonic acid metabolism
057_Fatty acid biosynthesis
058_Glycerolipid metabolism
059_Fatty acid metabolism
060_Steroid hormone biosynthesis
061_Bacterial secretion system
062_Phosphotransferase system (PTS)
063_ABC transporters
064_Porphyrin and chlorophyll metab…
065_Ubiquinone and other terpenoid-…
066_Pantothenate and CoA biosynthe…
067_Thiamine metabolism
068_Lipoic acid metabolism
069_Retinol metabolism
070_One carbon pool by folate
071_Folate biosynthesis
072_Nicotinate and nicotinamide met…
073_Biotin metabolism
074_Vitamin B6 metabolism
075_Riboflavin metabolism
076_Selenocompound metabolism
077_Phosphonate and phosphinate m…
078_Taurine and hypotaurine metabol…
079_Glutathione metabolism
080_Pyrimidine metabolism
081_Purine metabolism
082_Nucleotide excision repair
083_DNA replication
084_Homologous recombination
085_Mismatch repair
086_Base excision repair
087_Phosphatidylinositol signaling sy…
088_MAPK signaling pathway - yeast
089_Two-component system
090_Calcium signaling pathway
091_PI3K-Akt signaling pathway
092_Neuroactive ligand-receptor inter…
093_RNA polymerase
094_Ribosome
095_RNA transport
096_Aminoacyl-tRNA biosynthesis
097_Regulation of autophagy

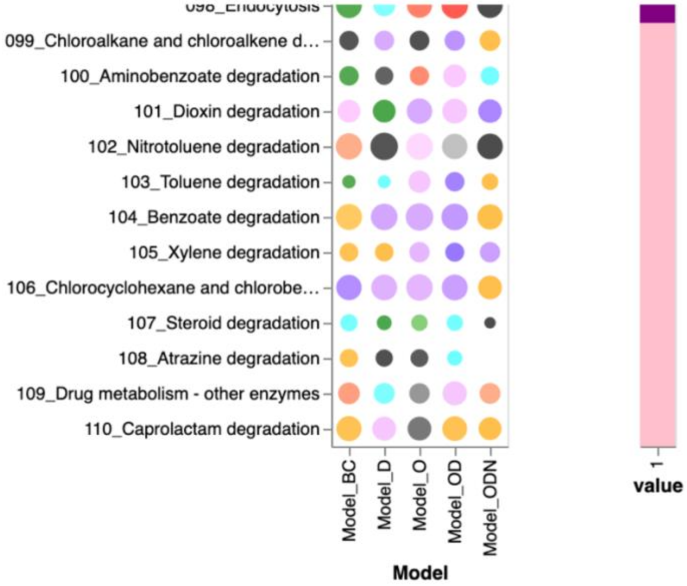


```
enrich_longformat2.to_csv("enrich_longformat2.txt", sep='\t', index=None)
#File enrich_longformat2.txt was used to generate Supplementary Figure 5
```
