# Supplementary material for: Metatranscriptomic response of deep ocean microbial populations to infusions of oil and/or synthetic chemical dispersant
Source: Appl Environ Microbiol. 2024 Jul 23;90(8):e01083-24. doi: 10.1128/aem.01083-24 (PMC11337851; doi:10.1128/aem.01083-24)
Supplement: SuppCode_2 — Supplemental code part 2. [file aem.01083-24-s0002.pdf]

Supporting code used for:

Metatranscriptomic response of deep ocean microbial populations to infusions of oil and/or synthetic chemical dispersant

–in submission to Applied and Environmental Microbiology

Click here for code for:

- Ordination analysis. Referenced in Figure 3A.
- MNTD MANOVA analysis. Referenced in the paper.

These results were also referenced in the working paper:

Working paper:
Peña-Montenegro et al. Colwellia and Marinobacter metapangenomes reveal species-specific responses to oil and dispersant exposure in deepsea microbial communities
https://doi.org/10.1101/2020.09.28.317438

```
In [1]:
#Following method at http://kembellab.ca/r-workshop/biodivR/SK_Biodivers
ity_R.html
library(picante)
library(reshape2)
```

Loading required package: ape
Loading required package: vegan
Loading required package: permute
Loading required package: lattice
This is vegan 2.5-5
Loading required package: nlme

```
setwd("/Users/tito-admin/Tito/JOYELABACKUP/SK_BACKUP/p22_Jupyter/Data/b-
diversity/")

comm_longfmt <- read.csv("/Users/tito-admin/Tito/JOYELABACKUP/SK_BACKUP/
p22_Jupyter/Data/Fig1_data_simple_absolute_melt.csv", header = TRUE, ro
w.names = 1)

#Transforming into compact(wide) format
comm = dcast(comm_longfmt, sample_names~variable)
comm$sample_names <- c('BC_0', 'BC_1', 'BC_2', 'BC_3', 'BC_4', 'D_0', 'D
_1', 'D_2', 'D_3', 'D_4', 'WAF_0', 'WAF_1A', 'WAF_1B', 'WAF_2', 'WAF_3',
'WAF_4A', 'WAF_4B', 'CEWAF_0', 'CEWAF_1A', 'CEWAF_1B', 'CEWAF_2', 'CEWAF
_3' ,'CEWAF_4A' ,'CEWAF_4B' , 'CEWAF+N_0' ,'CEWAF+N_1' , 'CEWAF+N_4')

#setting row names
comm2 <- comm[,-1]
rownames(comm2) <- comm[,1]
comm_v0 = comm
comm = comm2
```

In [4]:

```
comm['A'] <- comm$Bacteroidetes + comm$Flavobacteriaceae
comm$Bacteroidetes <- NULL
comm$Flavobacteriaceae <- NULL
colnames(comm)[colnames(comm)=="A"] <- "Bacteroidetes"
comm['A'] <- comm$Pseudomonas + comm$Gammaproteobacteria
comm$Pseudomonas <- NULL
comm$Gammaproteobacteria <- NULL
colnames(comm)[colnames(comm)=="A"] <- "Gammaproteobacteria"

head(rownames(comm))
```

'BC_0' 'BC_1' 'BC_2' 'BC_3' 'BC_4' 'D_0'

```
head(colnames(comm))
```

'Alcanivorax' 'Alphaproteobacteria' 'Alteromonadales' 'Archaea' 'Bacteria' 'Bermanella' . . .

```
comm[1:5,1:5]
```

A data.frame: 5 × 5

	Alcanivorax	Alphaproteobacteria	Alteromonadales	Archaea	Bacteria
	<int>	<int>	<int>	<int>	<int>
**BC_0**	7798	13340	21282	5913	16557
**BC_1**	3172	9134	11015	3471	10222
**BC_2**	2645	10407	15469	4818	13194
**BC_3**	1333	5680	8240	1636	7248
**BC_4**	849	3919	4792	1179	6460

In [8]:

```
#check total abundance in each sample
head(apply(comm, 1, sum))
```

**BC_0**	267276
**BC_1**	160747
**BC_2**	216421
**BC_3**	164783
**BC_4**	170340
**D_0**	195117

In [9]:

```
#Turn percent cover to relative abundance by diving each value by sample total abundance
comm <-decostand(comm, method="total")
#check total abundance in each sample
head(apply(comm, 1, sum))
```

**BC_0**	1
**BC_1**	1
**BC_2**	1
**BC_3**	1
**BC_4**	1
**D_0**	1

In [10]:

```
#look at the transformed data
comm[1:5,1:5]
```

A data.frame: 5 × 5

	Alcanivorax	Alphaproteobacteria	Alteromonadales	Archaea	Bacteria
	<dbl>	<dbl>	<dbl>	<dbl>	<dbl>
**BC_0**	0.029175833	0.04991095	0.07962556	0.022123198	0.06194720
**BC_1**	0.019732872	0.05682221	0.06852383	0.021592938	0.06359061
**BC_2**	0.012221550	0.04808683	0.07147643	0.022262165	0.06096451
**BC_3**	0.008089427	0.03446958	0.05000516	0.009928209	0.04398512
**BC_4**	0.004984149	0.02300693	0.02813197	0.006921451	0.03792415

Metadata

In [11]:

```
#replace filename with file.choose() to open interactive window
metadata <- read.csv('metadata_picante.csv', header=TRUE, row.names = 1)

#take a peek at the data
head(metadata)
```

A data.frame: 6 × 5

	Treatment	Dispersant	Oil	Nutrients	Time
	<fct>	<int>	<int>	<int>	<int>
**BC_0**	BC	0	0	0	0
**BC_1**	BC	0	0	0	7
**BC_2**	BC	0	0	0	17
**BC_3**	BC	0	0	0	28
**BC_4**	BC	0	0	0	42
**D_0**	D	1	0	0	0

Reference Tree

In [12]:

```
phy <- read.tree('v3_1000_iterations.newick')
```

In [13]:

```
phy
```

Phylogenetic tree with 27 tips and 26 internal nodes.

Tip labels:
Eukaryota, Chromista, Neoparamoeba, Archaea, Bacteroidetes, Fla vobacteriales, ...

Rooted; includes branch lengths.

In [14]:

```
plot(phy, cex=0.5)
```

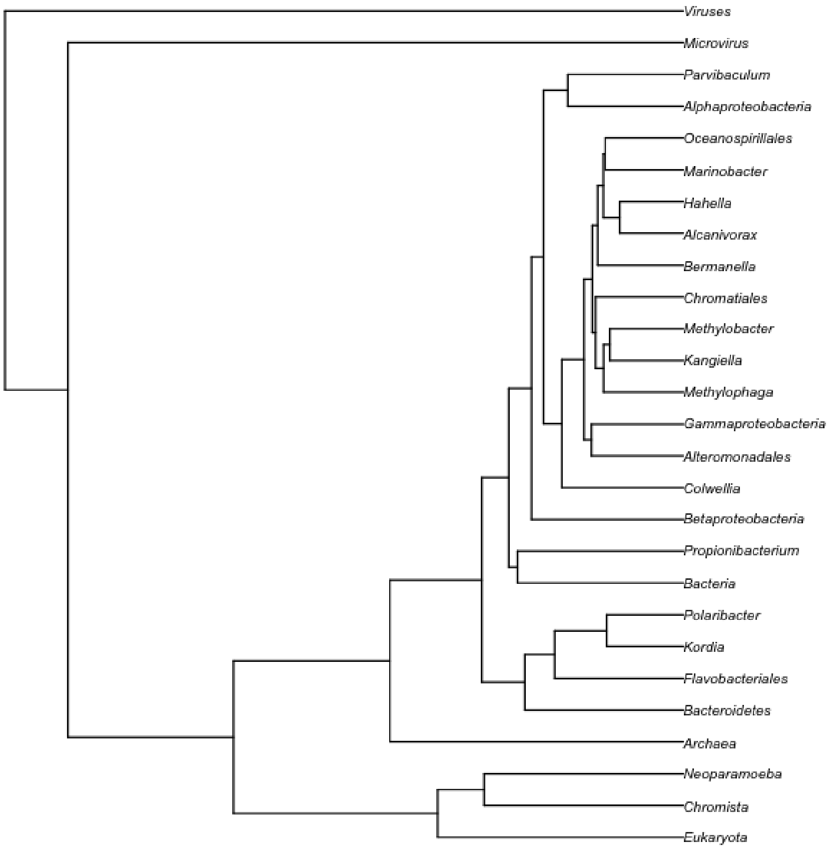

Cleaning and matching data sets

In [15]:

```
#check for mismatches/missing species
combined <- match.phylo.comm(phy, comm)
#the resulting object is a list with $phy and $data elements.
#Replace our original data with the sorted/matched data
phy <- combined$phy
comm <- combined$comm
```

In [16]:

```
#we should check whether our community data and metadata are in the same
order
all.equal(rownames(comm), rownames(metadata))
```

TRUE

Multivariate community analysis
How does the composition of microbial communities vary across different samples? How are Treatments and Time related to the microbial community composition?
We can use multivariate ordination methods to explore community structure in more detail. These methods are available in the vegan package, which also includes excellent documentation and tutorials for these methods. The book “Numerical Ecology in R” by Borcard et al. gives a great overview of multivariate analysis methods.

Hierarchical clustering
We can cluster together plots based on their overall community composition. We will calculate Bray-Curtis dissimilarity among all the samples, an abundance-weighted measure of how similar two communities are in terms of their species composition. We will then cluster together communities that are similar using an agglomerative hierarchical clustering algorithm.

In [19]:

```
# calculate Bray-Curtis distance among samples
comm.bc.dist <- vegdist(comm, method = "bray")
# cluster communities using average-linkage algorithm
comm.bc.clust <- hclust(comm.bc.dist, method = "average")
# plot cluster diagram
plot(comm.bc.clust, ylab = "Bray-Curtis dissimilarity")

svg(filename="FigS4G_Bray-Curtis_dissimilarity.svg")
plot(comm.bc.clust, ylab = "Bray-Curtis dissimilarity")
dev.off()
```

pdf: 2

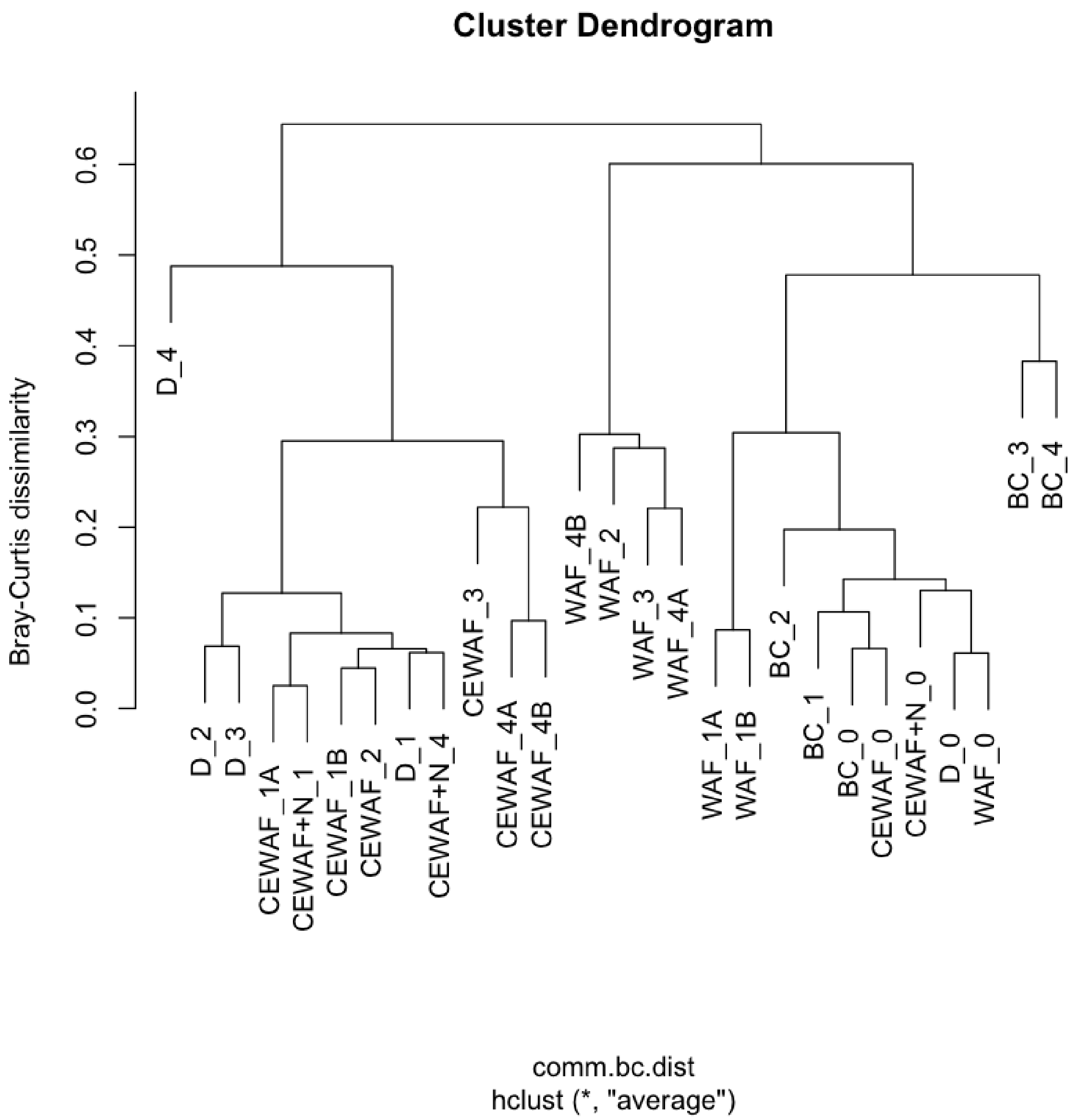

Ordination

There are numerous ordination methods available in R. For now, let's use non-metric multidimensional scaling to visualize the multivariate structure of these communities.

In [20]:

```
# The metaMDS function automatically transforms data and checks solution
# robustness
comm.bc.mds <- metaMDS(comm, dist = "bray")
```

```
Run 0 stress 0.08985495
Run 1 stress 0.09501596
Run 2 stress 0.1087268
```

```
Run 3 stress 0.08985495
... New best solution
... Procrustes: rmse 6.551262e-06 max resid 2.303139e-05
... Similar to previous best
Run 4 stress 0.1077623
Run 5 stress 0.1459412
Run 6 stress 0.1465701
Run 7 stress 0.09501569
Run 8 stress 0.08985495
... Procrustes: rmse 1.784849e-05 max resid 6.483347e-05
... Similar to previous best
Run 9 stress 0.09501596
Run 10 stress 0.1515174
Run 11 stress 0.1087268
Run 12 stress 0.1077607
Run 13 stress 0.09501596
Run 14 stress 0.1456336
Run 15 stress 0.08985496
... Procrustes: rmse 2.057956e-05 max resid 7.436334e-05
... Similar to previous best
Run 16 stress 0.1456336
Run 17 stress 0.08985495
... Procrustes: rmse 9.906887e-06 max resid 3.497065e-05
... Similar to previous best
Run 18 stress 0.1529206
Run 19 stress 0.09501596
Run 20 stress 0.1087082
*** Solution reached
```

In [21]:

```
# Assess goodness of ordination fit (stress plot)
```

```
stressplot(comm.bc.mds)
```

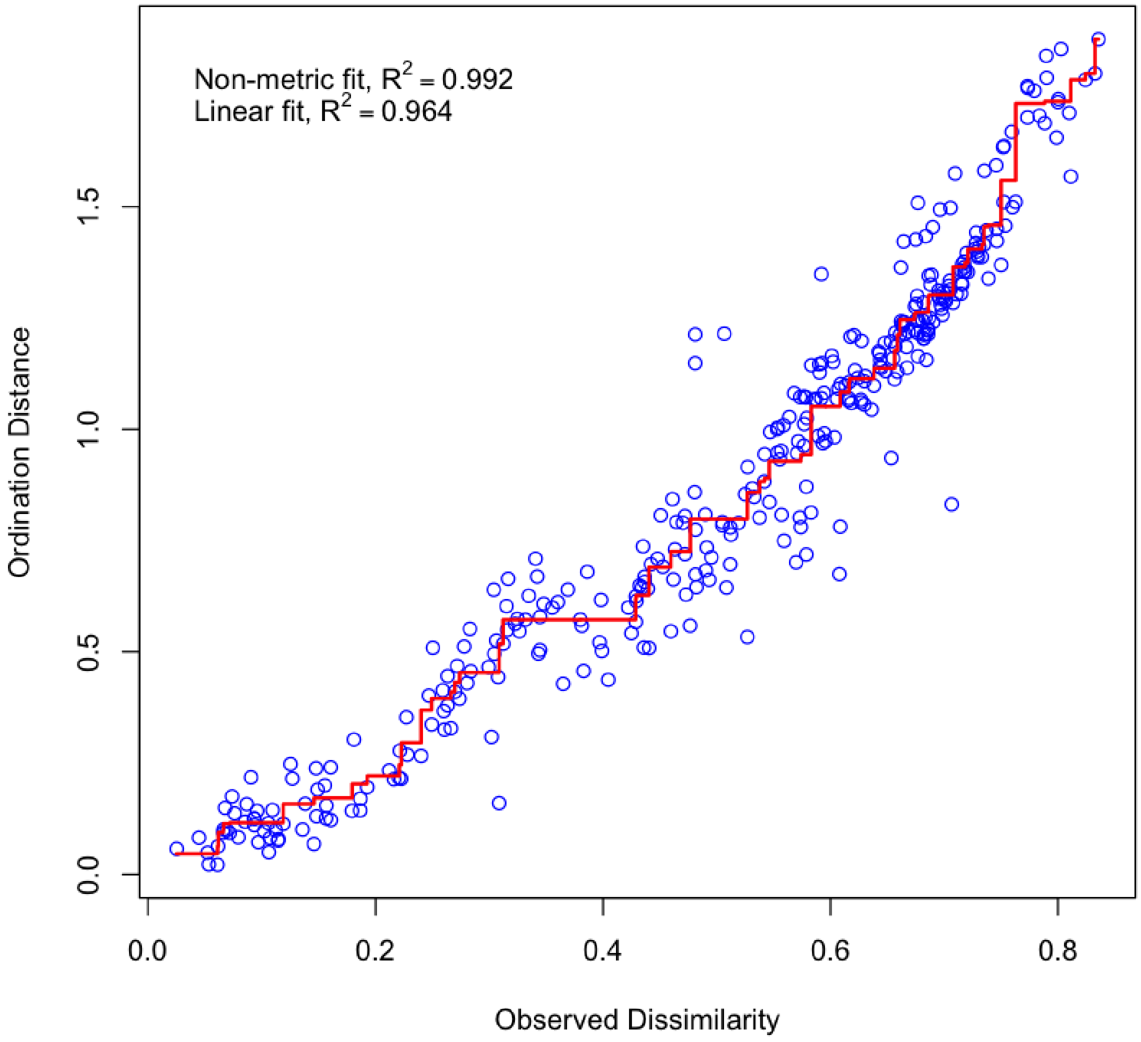

We can plot the ordination results in a variety of different ways.

In [22]:

```
# plot site scores as text
ordiplot(comm.bc.mds, display = "sites", type = "text")
```

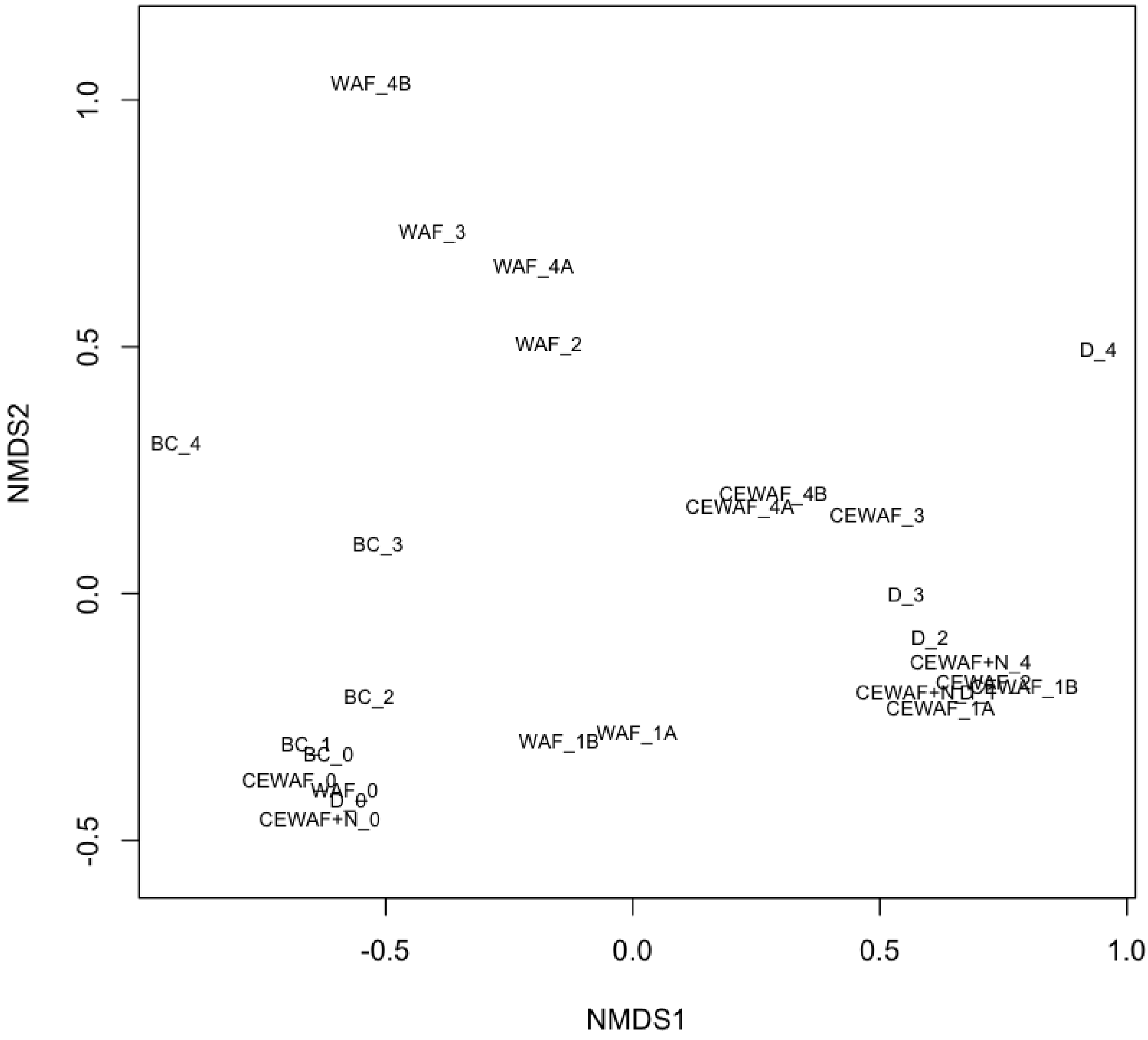

In

[23]:

```
p1 = comm.bc.mds$points p1 = as.data.frame(p1)
p1['Feature'] <- c('sample','sample','sample','sample','sample','sample'
,'sample','sample','sample','sample','sample','sample','sample','sample'
,'sample','sample','sample','sample','sample','sample','sample','sample'
,'sample','sample','sample','sample','sample')
p2 = comm.bc.mds$species
p2 = as.data.frame(p2)
p2['Feature'] <- c('species','species','species','species','species','sp
ecies','species','species','species','species','species','species','spec
ies','species','species','species','species','species','species','specie
s','species','species','species','species','species','species','species')
```

```
nmds_data <- rbind(p1,p2)
write.csv(nmds_data, file = "Fig3_nmds_data_for_plot.csv")

ordipointlabel(comm.bc.mds)
```

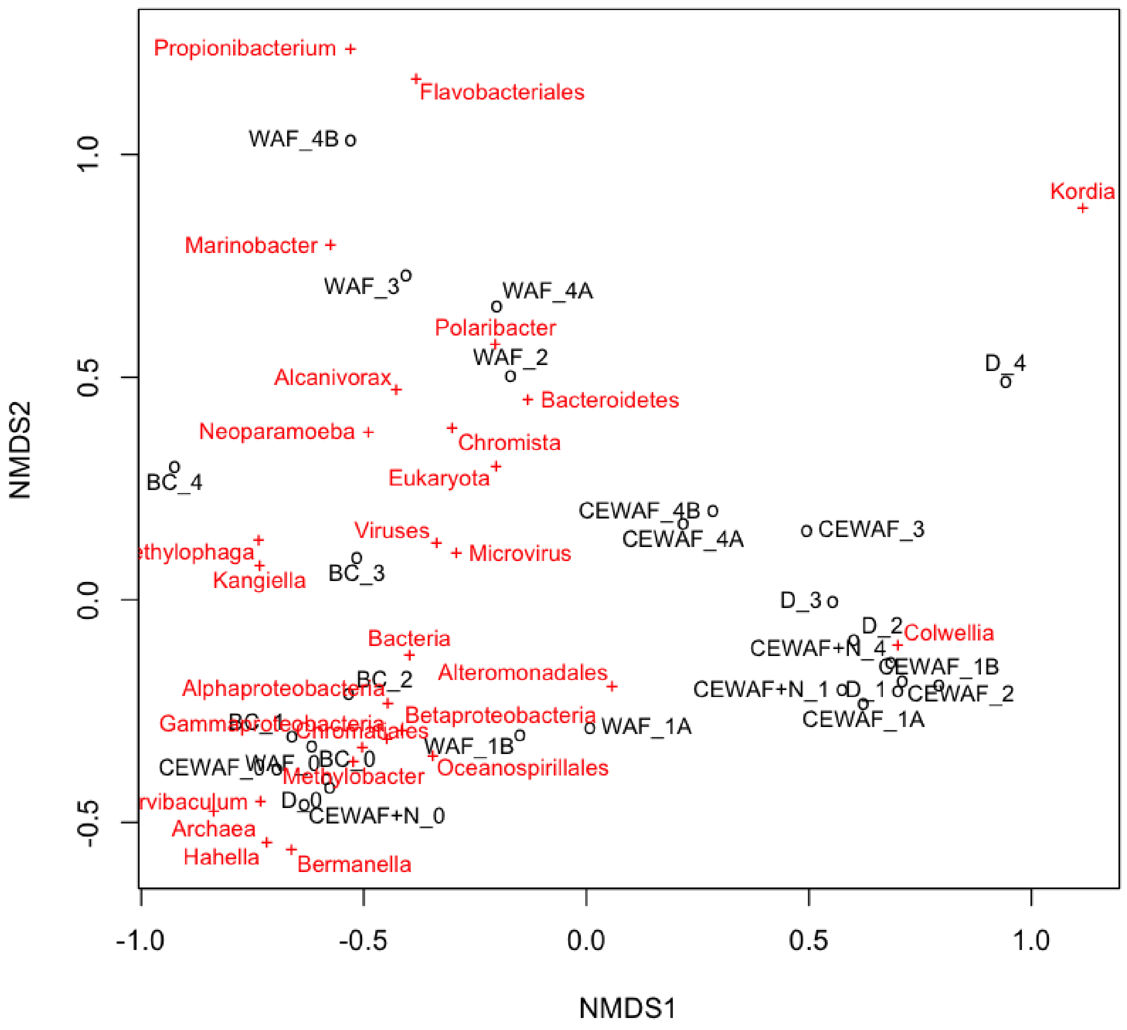

In [25]:

```
# plot Colwellia abundance. cex increases the size of bubbles.
ordisurf(comm.bc.mds, comm[, "Colwellia"], bubble = TRUE, main = "Colwellia abundance",
cex = 3)
```

Family: gaussian
Link function: identity

Formula:

y ~ s(x1, x2, k = 10, bs = "tp", fx = FALSE)

Estimated degrees of freedom:
8.91 total = 9.91

REML score: -55.32544

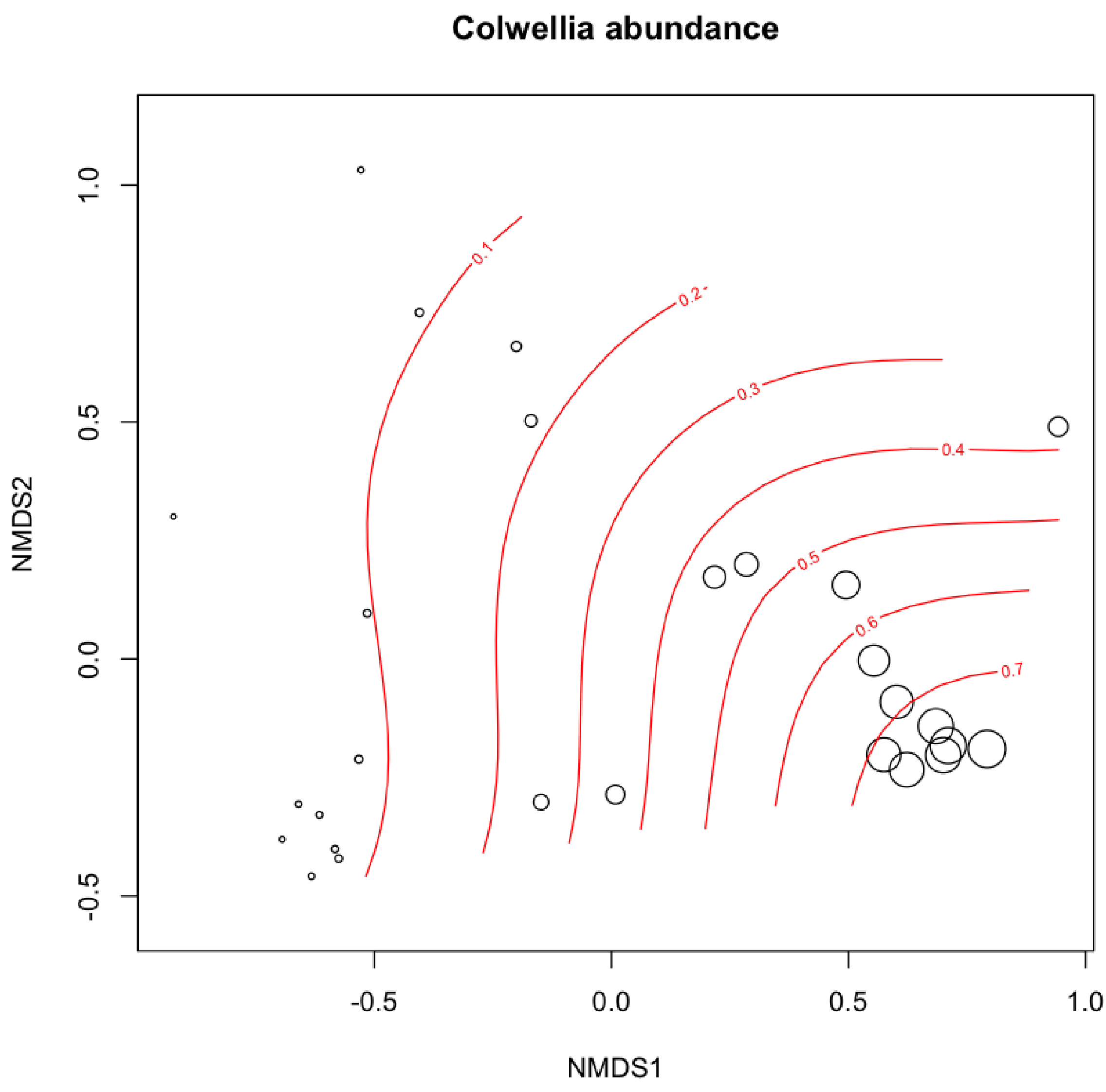

In [26]:

```
# plot Colwellia abundance. cex increases the size of bubbles.
ordisurf(comm.bc.mds, comm[, "Marinobacter"], bubble = TRUE, main = "Mar
inobacter abundance", cex = 3)
```

Family: gaussian

Link function: identity

Formula:
y ~ s(x1, x2, k = 10, bs = "tp", fx = FALSE)

Estimated degrees of freedom:
7.57 total = 8.57

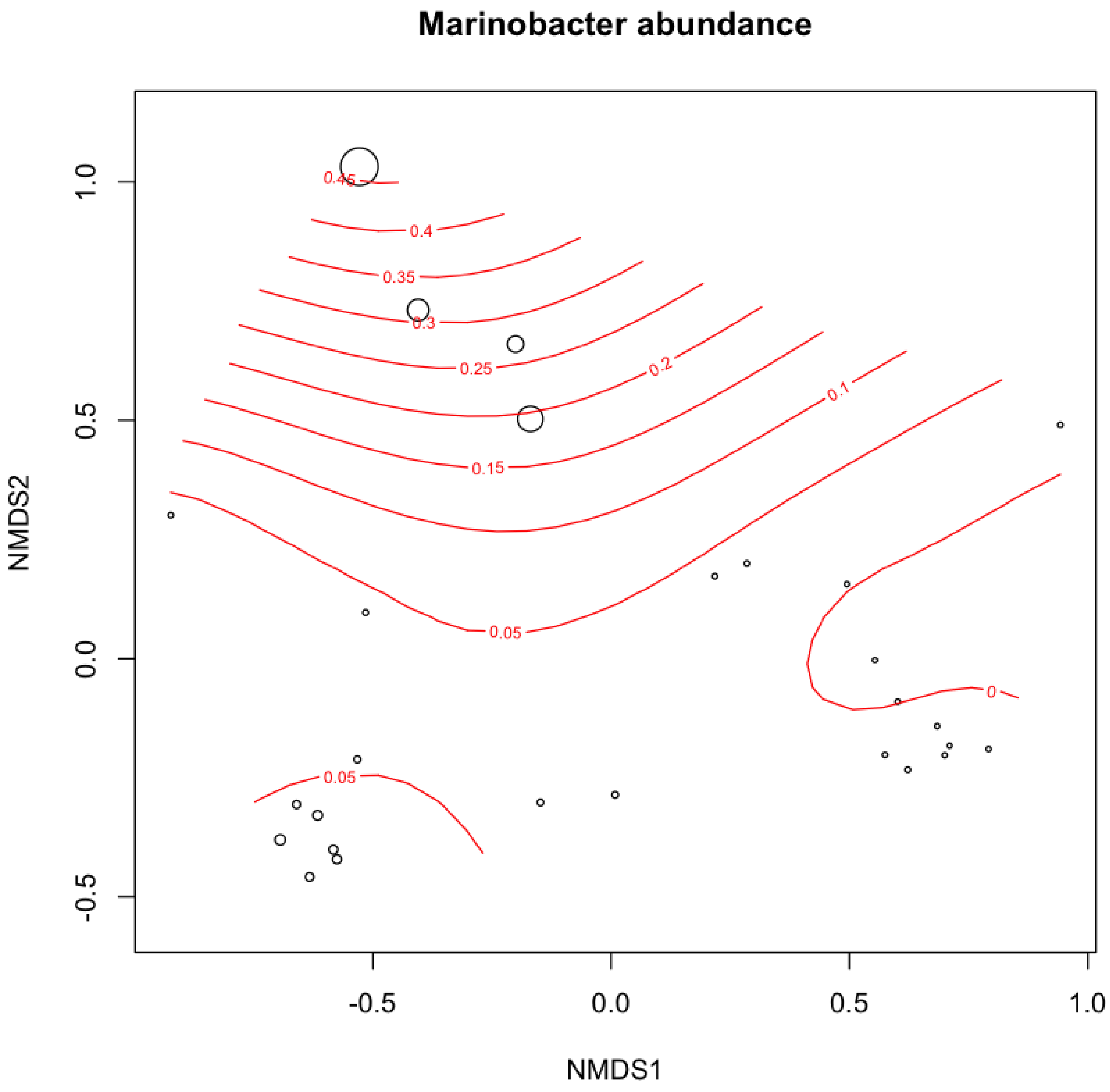

REML score: -32.06745

In [27]:

```
ordiplot(comm.bc.mds)
# calculate and plot environmental variable correlations with the axes
# use the subset of metadata that are environmental data
plot(envfit(comm.bc.mds, metadata[, 2:4]))
```

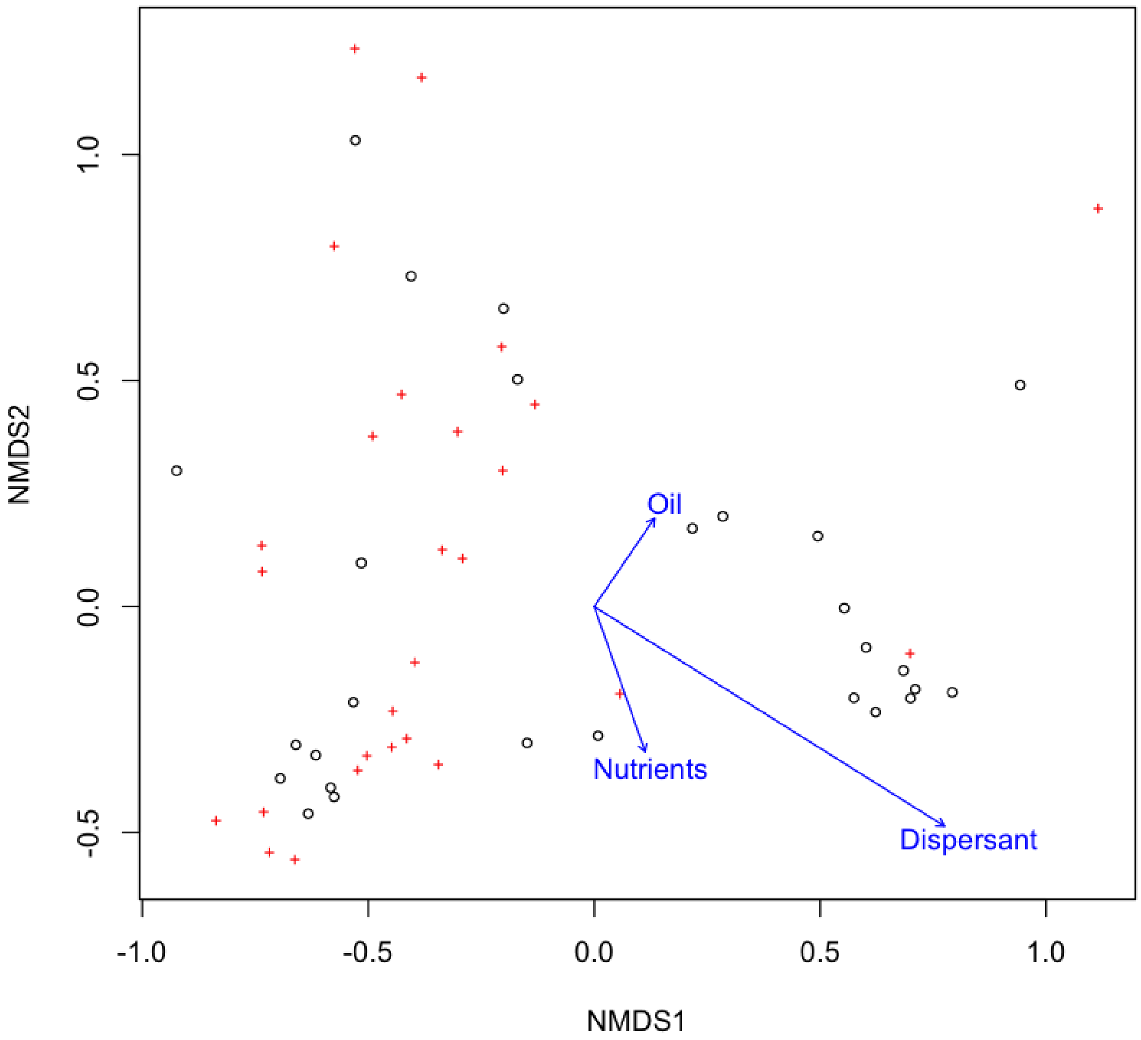

Ordination II

Doing the same thing but with PCA

In [64]:

```
# First step is to calculate a distance matrix.
# Here we use Bray-Curtis distance metric
comm.bc.vegdist <- vegdist(comm, method = "bray")
```

In [65]:

```
# PCoA is not included in vegan.
# We will use the ape package instead
library(ape)
PCOA <- pcoa(comm.bc.vegdist)
```

In [66]:

```
PCOA$values$Relative_eig[1:10]
```

0.553453314549708
0.237784428777649
0.0993224339555511
0.0580317706709635
0.0234286351306846
0.0202983330356126
0.0137114889067147
0.00917993330779548
0.00646329818539521
0.00551553490197085

In [67]:

```
Y = comm
x = PCOA
plot.axes = c(1, 2)
pr.coo <- x$vectors
n <- nrow(Y)
points.stand <- scale(pr.coo[, plot.axes])
S <- cov(Y, points.stand)
U <- S %*% diag((x$values$Eigenvalues[plot.axes]/(n - 1))^(-0.5))

#We need is points.stand and U to export to be plotted in altair
```

In [70]:

```
# Write CSV in R
write.csv(points.stand, file = "Fig3_pca_samples.csv")
write.csv(U, file = "Fig3_pca_species.csv")
```

MPD, MNTD, SES_{MPD}, and SES_{MNTD}

Another way of thinking about the phylogenetic relatedness of species in a community is to ask 'how closely related are the average pair of species or individuals in a community', and relate the patterns we observe to what we'd expect under various null models of evolution and community assembly. These types of questions are addressed by the measures of community phylogenetic structure such as MPD, MNTD, NRI and NTI described by Webb et al. and implemented in Phylocom.

The function mpd will calculate the mean pairwise distance between all species or individuals in each community. Similarly, the mntd function calculates the mean nearest taxon distance, the average distance separating each species or individual in the community from its closest heterospecific relative. The mpd and mntd functions differs slightly from the pd function in that they take a distance matrix as input rather than a phylogeny object. A phylo object can be converted to a interspecific phylogenetic distance matrix using the cophenetic function. Since the mpd and mntd functions can use any distance matrix as input, we can easily calculate trait diversity measures by substituting a trait distance matrix for the phylogenetic distance matrix. We'll return to this idea shortly.

If the community data represent abundance measures, the abundance data can be taken into account. Doing so changes the interpretation of these metrics from the average distance among two randomly chosen species from a community, to the average distance among two randomly chosen individuals in a community.

Measures of 'standardized effect size' of phylogenetic community structure can be calculated for MPD and MNTD by compared observed phylogenetic relatedness to the pattern expected under some null model of phylogeny or community randomization. Standardized effect sizes describe the difference between average phylogenetic distances in the observed communities versus null communities generated with some randomization method, standardized by the standard deviation of phylogenetic distances in the null data:

$$SES_{metric} = \frac{Metric_{observed} - mean(Metric_{null})}{sd(Metric_{null})}$$

Phylocom users will be familiar with the measures NRI and NTI; (SES*{MPD}) and (SES*{MNTD}) are equivalent to -1 times NRI and NTI, respectively. Several different null models can be used to generate the null communities. These include randomizations of the tip labels of the phylogeny, and various community randomizations that can hold community species richness and/or species occurrence frequency constant. These are described in more detail in the help files, as well as in the Phylocom manual. Let's calculate some of these measures of community phylogenetic structure for our example data set. We will ignore abundance information and use a simple null model of randomly drawing species while keeping sample species richness constant.

In [21]:

```
# convert phylogenety to a distance matrix
```

```
phy.dist <- cophenetic(phy)
```

In [21]:

```
# calculate ses.mpd
comm.sesmpd <- ses.mpd(comm, phy.dist, null.model = "taxa.labels", abund
ance.weighted = TRUE, runs = 999)
head(comm.sesmpd)
```

A data.frame: 6 × 8

	ntaxa	mpd.obs	mpd.rand.mean	mpd.rand.sd	mpd.obs.rank	mpd.obs.z	mpd.obs.p	ru
	<int>	<dbl>	<dbl>	<dbl>	<dbl>	<dbl>	<dbl>	<db
**BC_0**	27	0.2704630	0.4261616	0.07993540	7	-1.9478050	0.007	9
**BC_1**	27	0.3046536	0.4251318	0.08146594	32	-1.4788781	0.032	9
**BC_2**	27	0.2967192	0.4294765	0.08053378	25	-1.6484668	0.025	9
**BC_3**	27	0.3604121	0.4288612	0.08088532	222	-0.8462488	0.222	9
**BC_4**	27	0.3865453	0.3983323	0.10492300	563	-0.1123392	0.563	9
**D_0**	26	0.2701752	0.4175235	0.08934273	21	-1.6492471	0.021	9

In [29]:

```
# calculate ses.mntd
comm.sesmntd <- ses.mntd(comm, phy.dist, null.model = "taxa.labels", abu
ndance.weighted = TRUE, runs = 999)


head(comm.sesmntd)
```

A data.frame: 6 × 8

	ntaxa	mntd.obs	mntd.rand.mean	mntd.rand.sd	mntd.obs.rank	mntd.obs.z	mntd.obs.p
	<int>	<dbl>	<dbl>	<dbl>	<dbl>	<dbl>	<dbl>
**BC_0**	27	0.2036847	0.2638289	0.05665282	97	-1.0616278	0.097
**BC_1**	27	0.2262502	0.2639562	0.05809908	247	-0.6489951	0.247
**BC_2**	27	0.2168126	0.2642766	0.05407254	187	-0.8777822	0.187
**BC_3**	27	0.2260593	0.2640515	0.05504341	277	-0.6902227	0.277
**BC_4**	27	0.2339265	0.2642017	0.08968832	463	-0.3375604	0.463
**D_0**	26	0.2074234	0.2643301	0.06407793	165	-0.8880855	0.165

The output includes the following columns:

- ntaxa - Number of taxa in community
- mpd.obs - Observed mpd in community
- mpd.rand.mean - Mean mpd in null communities
- mpd.rand.sd - Standard deviation of mpd in null communities
- mpd.obs.rank - Rank of observed mpd vs. null communities
- mpd.obs.z - Standardized effect size of mpd vs. null communities (equivalent to -NRI)
- mpd.obs.p - P-value (quantile) of observed mpd vs. null communities (= mpd.obs.rank / runs + 1) runs - Number of randomizations

Positive SES values (mpd.obs.z > 0) and high quantiles (mpd.obs.p > 0.95) indicate phylogenetic evenness, while negative SES values and low quantiles (mpd.obs.p < 0.05) indicate phylogenetic clustering, relative to the null model. MPD is generally thought to be more sensitive to tree-wide patterns of phylogenetic clustering and eveness, while MNTD is more sensitive to patterns of evenness and clustering closer to the tips of the phylogeny.

In [30]:

```
# compare ses.mpd between habitats
plot(comm.sesmpd$mpd.obs.z ~ metadata$Treatment, xlab = "Treatment", ylab
= "SES(MPD)")
abline(h = 0, col = "gray")
```

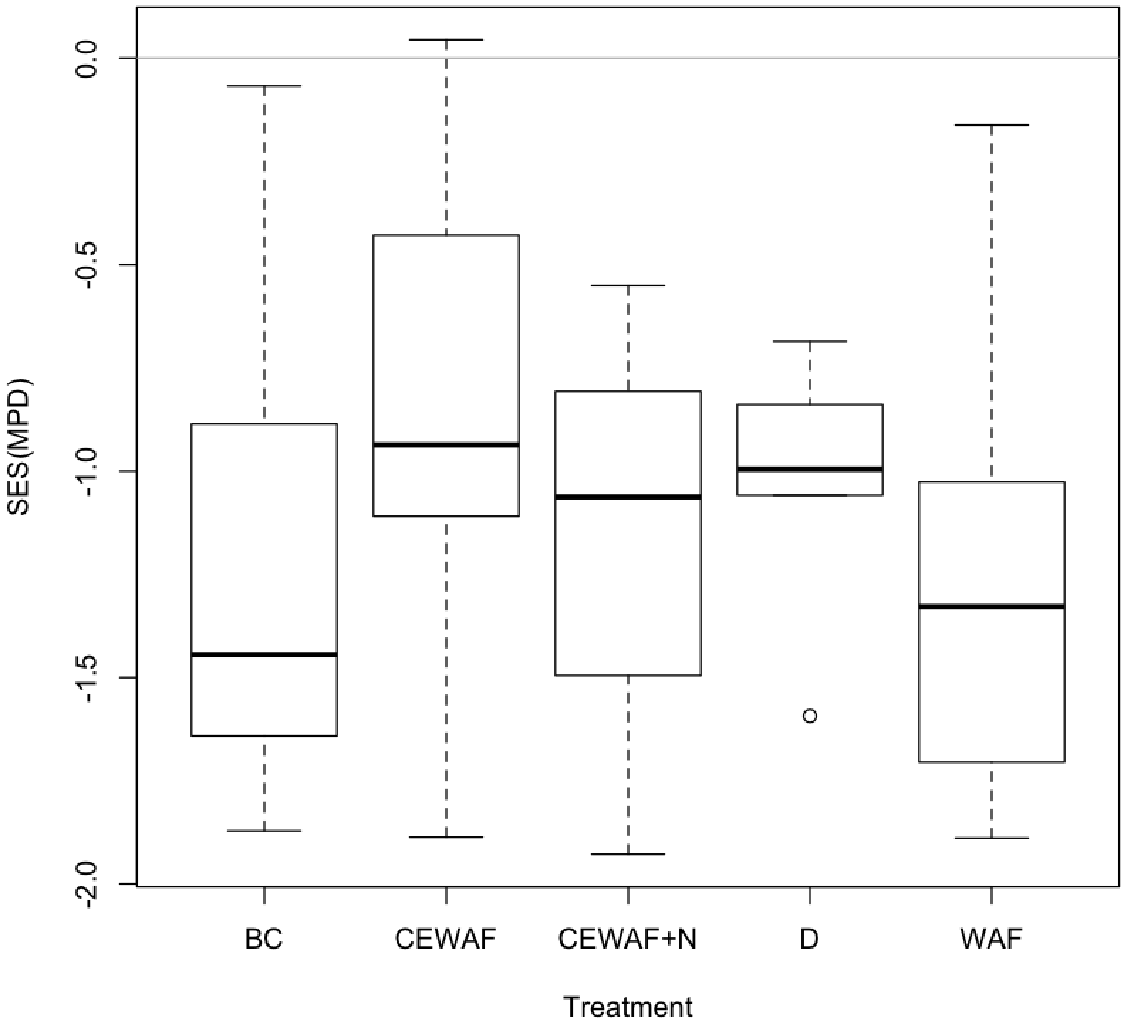

In [31]:

```
# compare ses.mpd between habitats
plot(comm.sesmpd$mpd.obs.z ~ metadata$Time, xlab = "Time", ylab =
"SES(MPD)")
abline(h = 0, col = "gray")
```

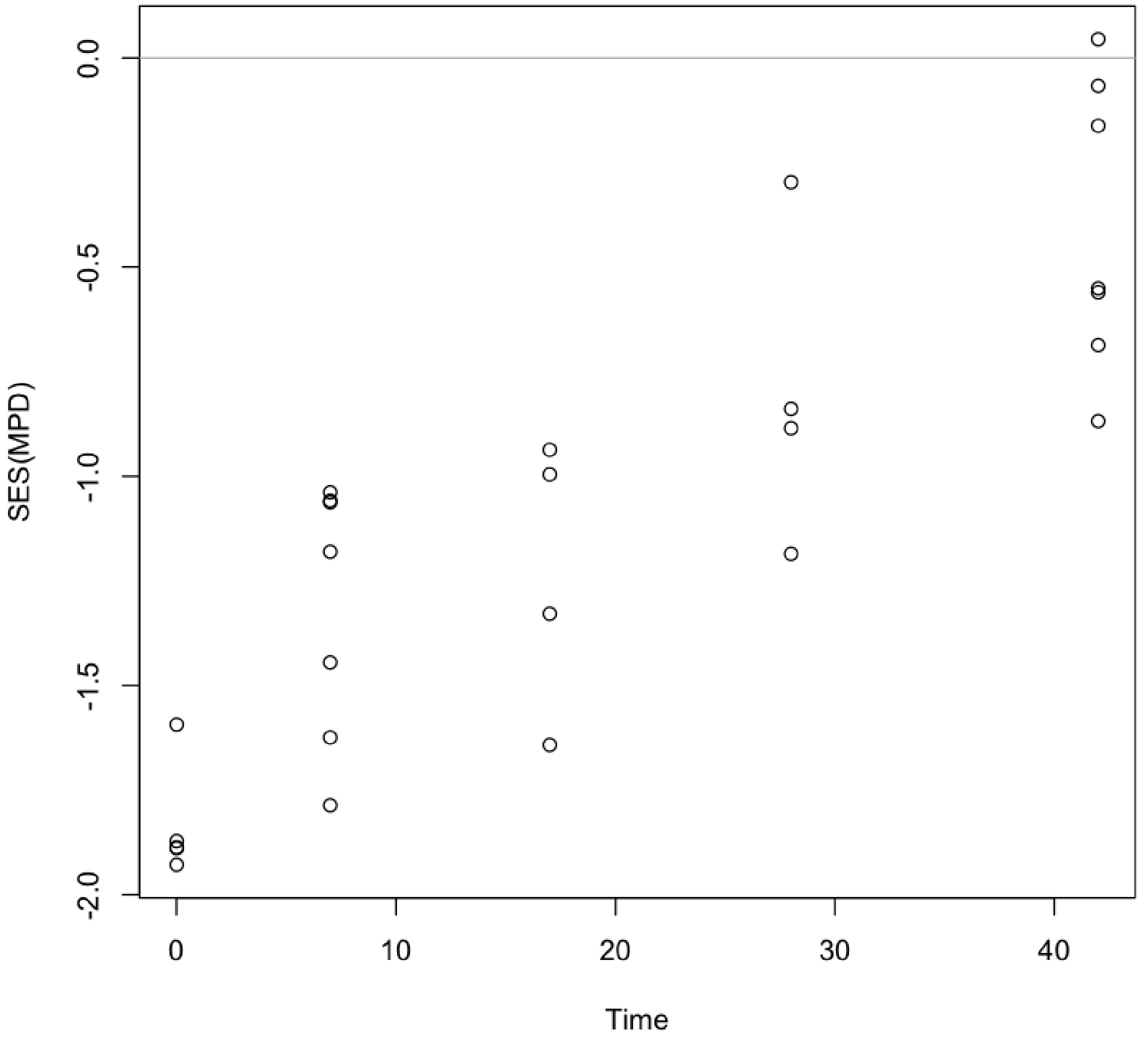

In [45]:

```
# Compute the analysis of variance
res.aov <- aov(comm.sesmpd$mpd.obs.z ~ metadata$Treatment + metadata$Tim
e, data = comm)
```

```
# Summary of the analysis
summary(res.aov)
```

	Df	Sum Sq	Mean Sq	F value	Pr(>F)	
metadata$Treatment	4	0.743	0.186	2.313	0.0912	.
metadata$Time	1	6.436	6.436	80.174	1.29e-08	***
Residuals	21	1.686	0.080			

Signif. codes: 0 ‘***’ 0.001 ‘**’ 0.01 ‘*’ 0.05 ‘.’ 0.1 ‘ ’ 1

In [46]:

```
# compare ses.mntd between habitats
plot(comm.sesmntd$mntd.obs.z ~ metadata$Treatment, xlab = "Treatment", y
lab = "SES(MNTD)")
abline(h = 0, col = "gray")
```

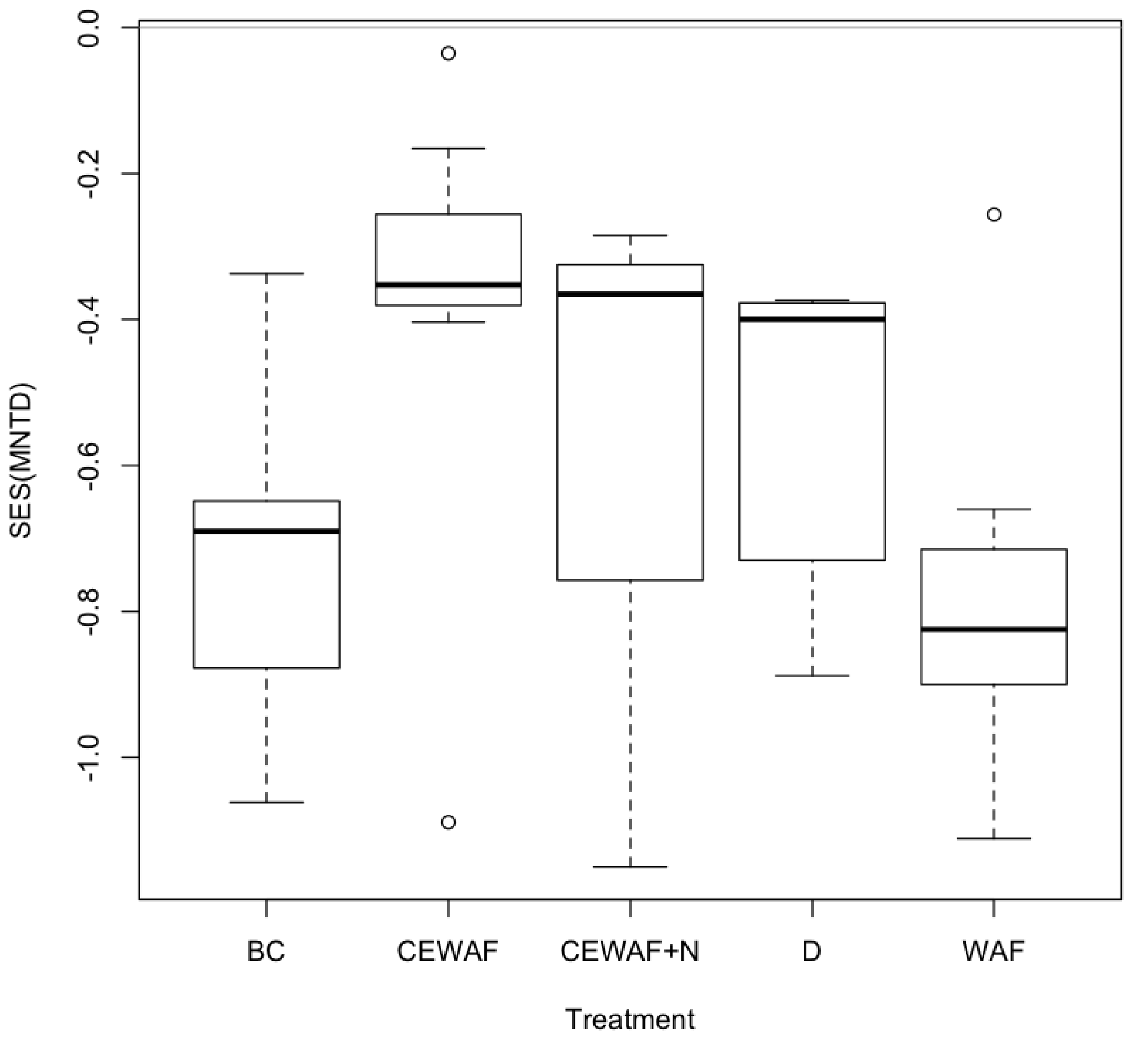

In [47]:

```
# compare ses.mntd between habitats
plot(comm.sesmntd$mntd.obs.z ~ metadata$Time, xlab = "Time", ylab = "SES
(MNTD)")
abline(h = 0, col = "gray")
```

In [48]:

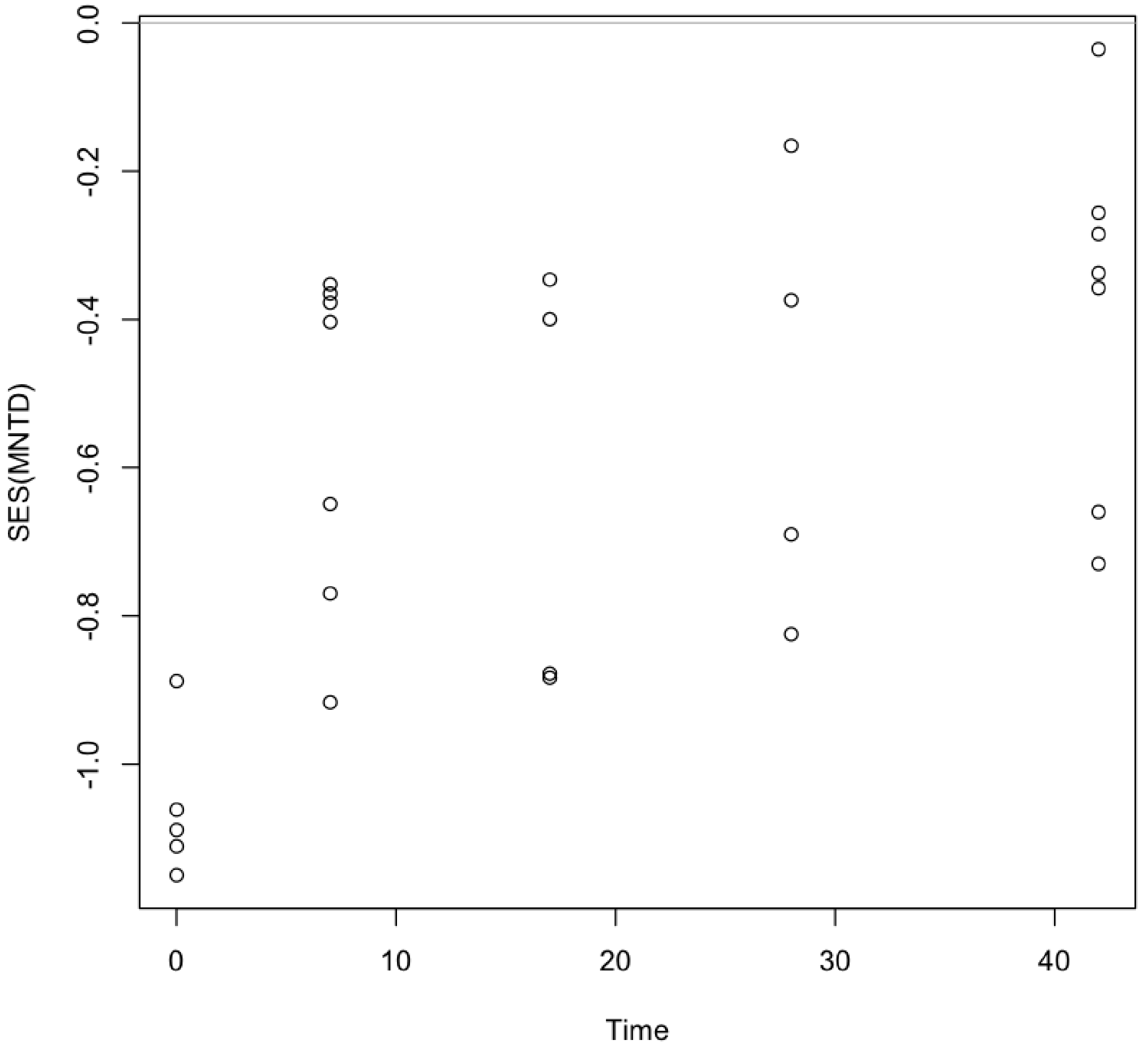


```
# Compute the analysis of variance
res.aov <- aov(comm.sesmntd$mntd.obs.z ~ metadata$Treatment +
metadata$Time, data = comm)
# Summary of the analysis
summary(res.aov)
```

```
                          Df   Sum Sq    Mean Sq  F value  Pr(>F)
metadata$Treatment  4    0.5994    0.1499    2.636    0.06293 .
metadata$Time     1    0.8851    0.8851   15.569   0.00074 ***
Residuals                  21   1.1939    0.0569
---
Signif. codes: 0 '***' 0.001 '**' 0.01 '*' 0.05 '.' 0.1 ' '
```

Phylogenetic diversity analysis

One of the earliest measures of phylogenetic relatedness in ecological communities was the phylogenetic diversity (PD) index proposed by Faith. Faith's PD is defined as the total branch length spanned by the tree including all species in a local community, optionally including the root node of the phylogeny. The pd function returns two values for each community, Faith's PD and species richness (SR).

In [49]:

```
#TEST IF IT IS ROOTED
is.rooted(phy)
```

TRUE

In [49]:

```
#rootedphy = root(phy, outgroup = 'Cenarchaeum',resolve.root = TRUE)
```

In [50]:

```
# Calculate Faith's PD comm.pd <- pd(comm, phy)
head(comm.pd)
```

A data.frame: 6 × 2

	PD	SR
	<dbl>	<dbl>
**BC_0**	4.402632	27
**BC_1**	4.402632	27
**BC_2**	4.402632	27
**BC_3**	4.402632	27
**BC_4**	4.402632	27
**D_0**	4.241044	26

In [51]:

```
# Plot Faith's PD by habitat
boxplot(comm.pd$PD ~ metadata$Treatment, xlab = "Treatment", ylab =
"Faith's PD")
```

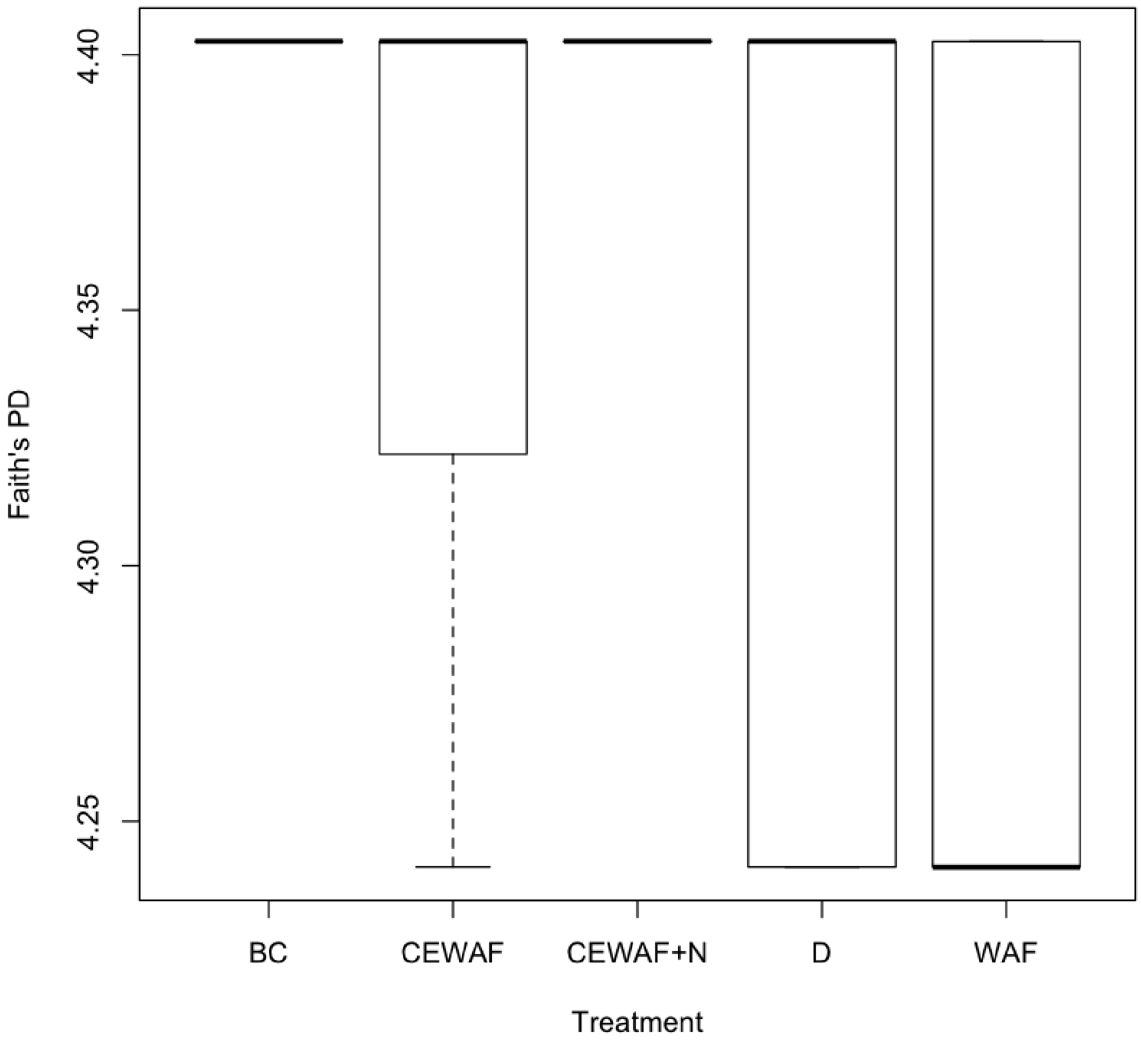

In [52]:

```
# Test for PD differences among habitats
# Compute the analysis of variance
res.aov <- aov(comm.pd$PD ~ metadata$Treatment + metadata$Time, data = c omm)

# Summary of the analysis
summary(res.aov)
```

	Df	Sum Sq	Mean Sq	F value	Pr(>F)
metadata$Treatment	4	0.03360	0.00840	2.308	0.09166 .
metadata$Time	1	0.03698	0.03698	10.161	0.00443 **
Residuals	21	0.07642	0.00364		

Signif. codes: 0 ‘***’ 0.001 ‘**’ 0.01 ‘*’ 0.05 ‘.’ 0.1 ‘ ’ 1

In [53]:

```
# Compare PD and species richness
plot(comm.pd$PD ~ comm.pd$SR, xlab = "Species richness", ylab = "Faith's
PD")
```

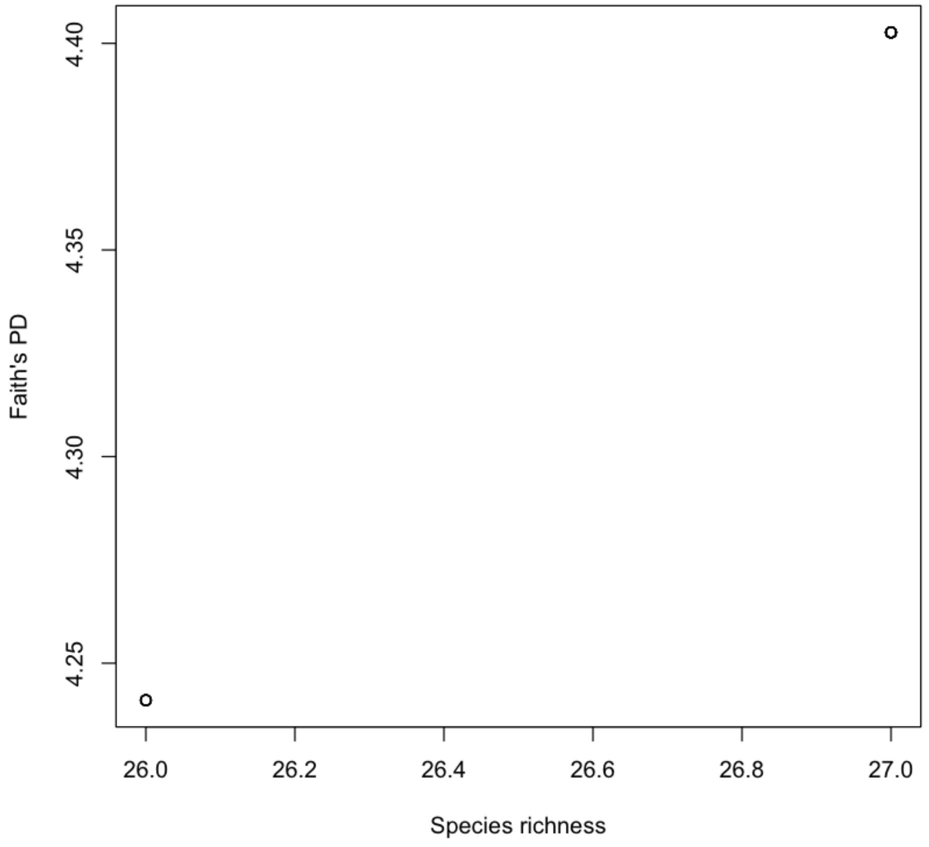

In [17]:

```
# convert phylogenety to a distance matrix
phy.dist <- cophenetic(phy)
```

Phylogenetic beta-diversity

We can measure patterns of phylogenetic relatedness among communities in a manner similar to the within-community phylogenetic diversity measures described above. The unifrac and phylosor functions measure the among-community equivalent of Faith's PD, the total unique/shared branch length between communities. The comdist and comdistnt functions measure the among-community equivalent of MPD and MNTD, the mean pairwise distance or mean nearest taxon distance between pairs of species drawn from two distinct communities. Let's compare a few different ways of measuring dissimilarity among communities. We've already calculated the Bray-Curtis distance among communities based on shared species (comm.bc.dist). Since the Bray-Curtis distance incorporates species abundances, we should use abundance information when calculating phylogenetic and trait diversity as well.

In [23]:

```
# calculate phylogenetic MNTD beta diversity
comm.mntd.dist <- comdistnt(comm, phy.dist, abundance.weighted = TRUE)
# calculate Mantel correlation for taxonomic Bray-Curtis vs.
phylogenetic
# MNTD diversity
mantel(comm.bc.dist, comm.mntd.dist)
```

```
Mantel statistic based on Pearson's product-moment correlation

Call:
mantel(xdis = comm.bc.dist, ydis = comm.mntd.dist)

Mantel statistic r: 0.04673 Significance:
      0.219

Upper quantiles of permutations (null model):
   90%    95% 97.5%    99%
0.0806 0.1090 0.1351 0.1617
Permutation: free
Number of permutations: 999
```

In [26]:

```
# calculate phylogenetic MPD beta diversity
comm.mpd.dist <- comdist(comm, phy.dist, abundance.weighted = TRUE)
```

Phylogeny/trait-based ordinations

Since we can calculate phylogeny- and trait-based measures of dissimilarity among samples, we can also perform an ordination of samples based on these metrics. Let's compare phylogeny- and trait-based ordinations with the species-based ordination we performed earlier.

In [23]:

```
# NMDS ordination of phylogenetic distances - use monoMDS since we only
```

```r
# have among-sample distances
comm.mntd.mds <- monoMDS(comm.mntd.dist)


# Assess goodness of ordination fit (stress plot)
stressplot(comm.mntd.mds)
```

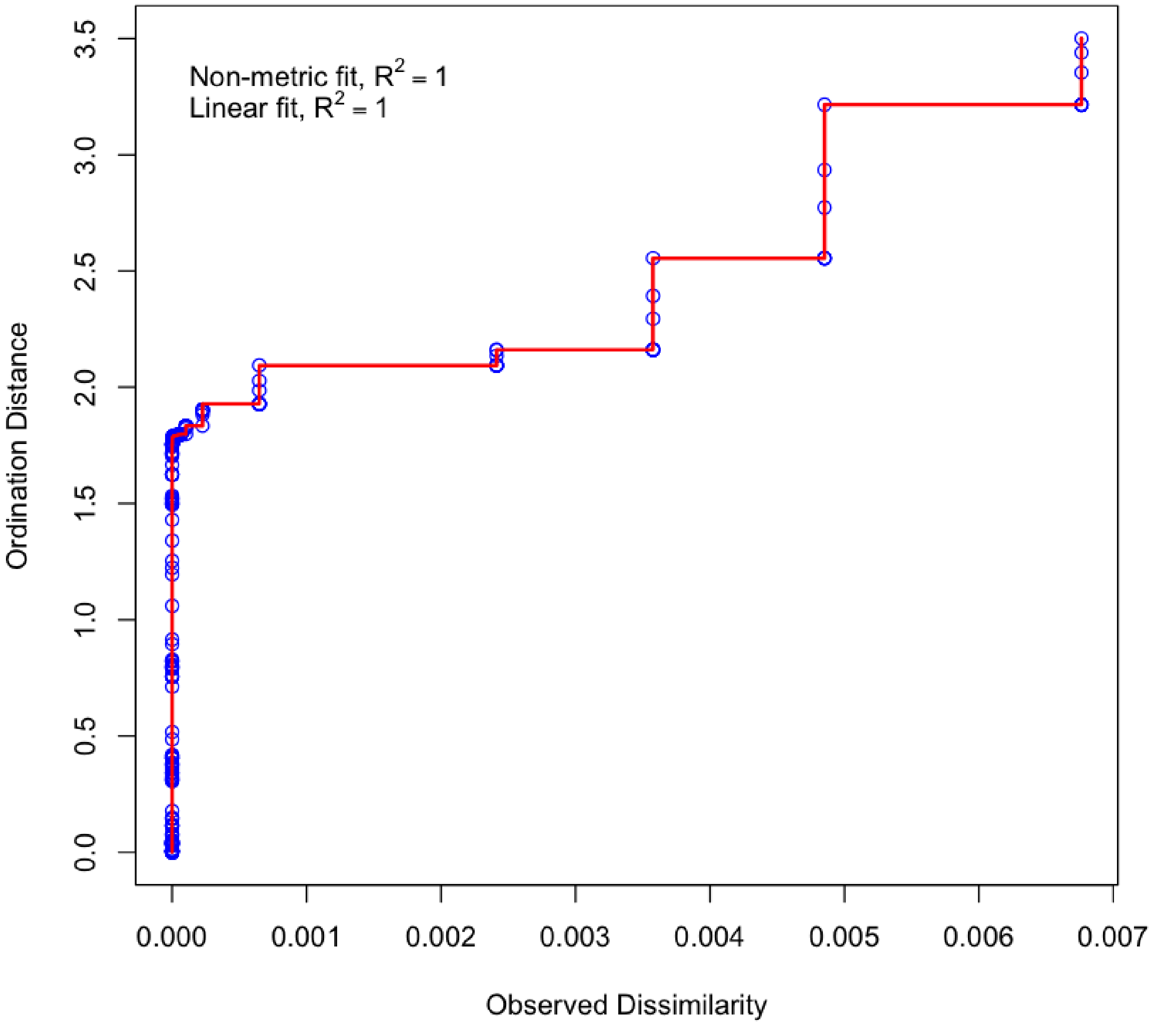

In [26]:

```
# plot site scores as text
ordiplot(comm.mntd.mds, display = "sites", type = "text")
```

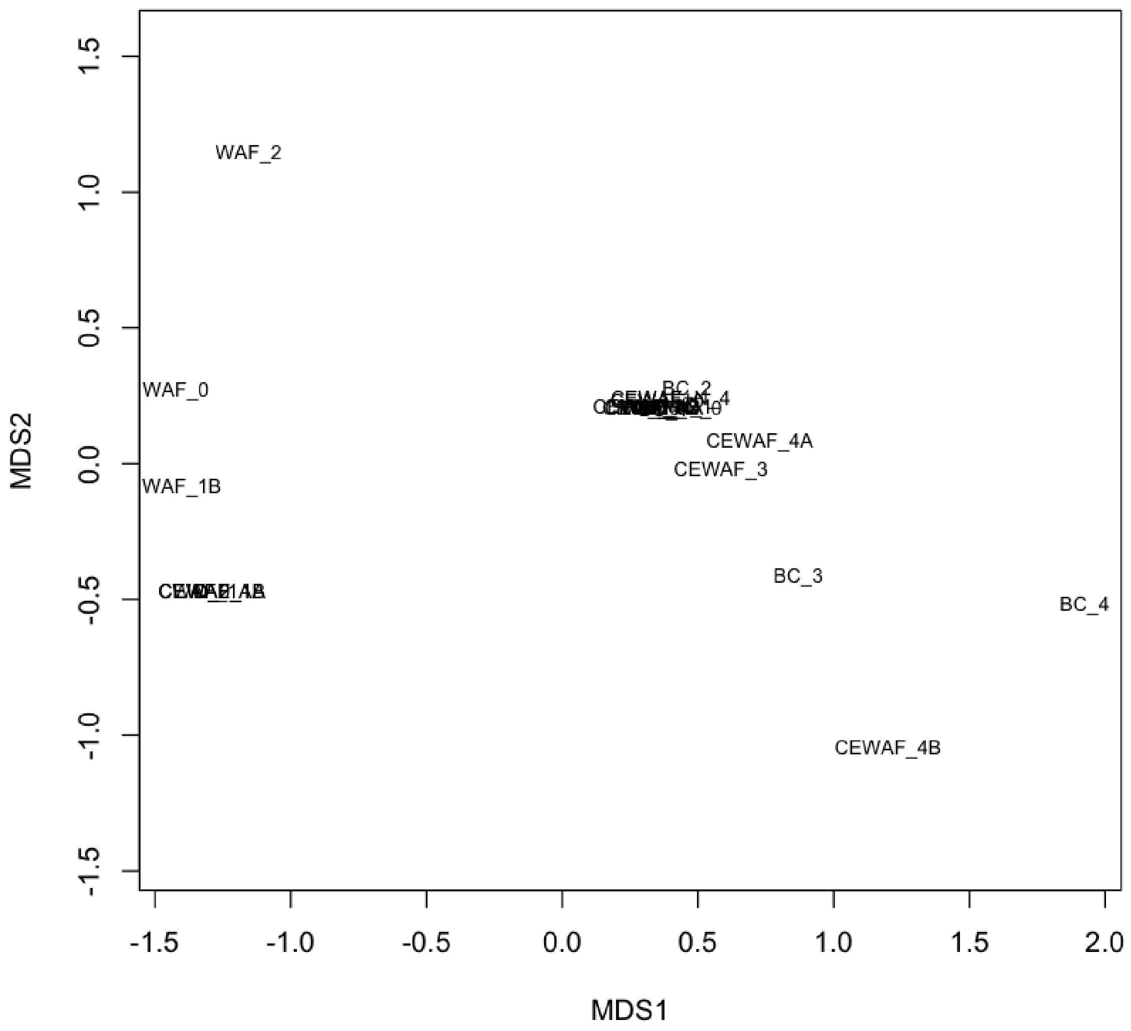

In [28]:

```
# automated plotting of results - tries to eliminate overlapping labels
ordipointlabel(comm.mntd.mds)
```

```
species scores not available
```

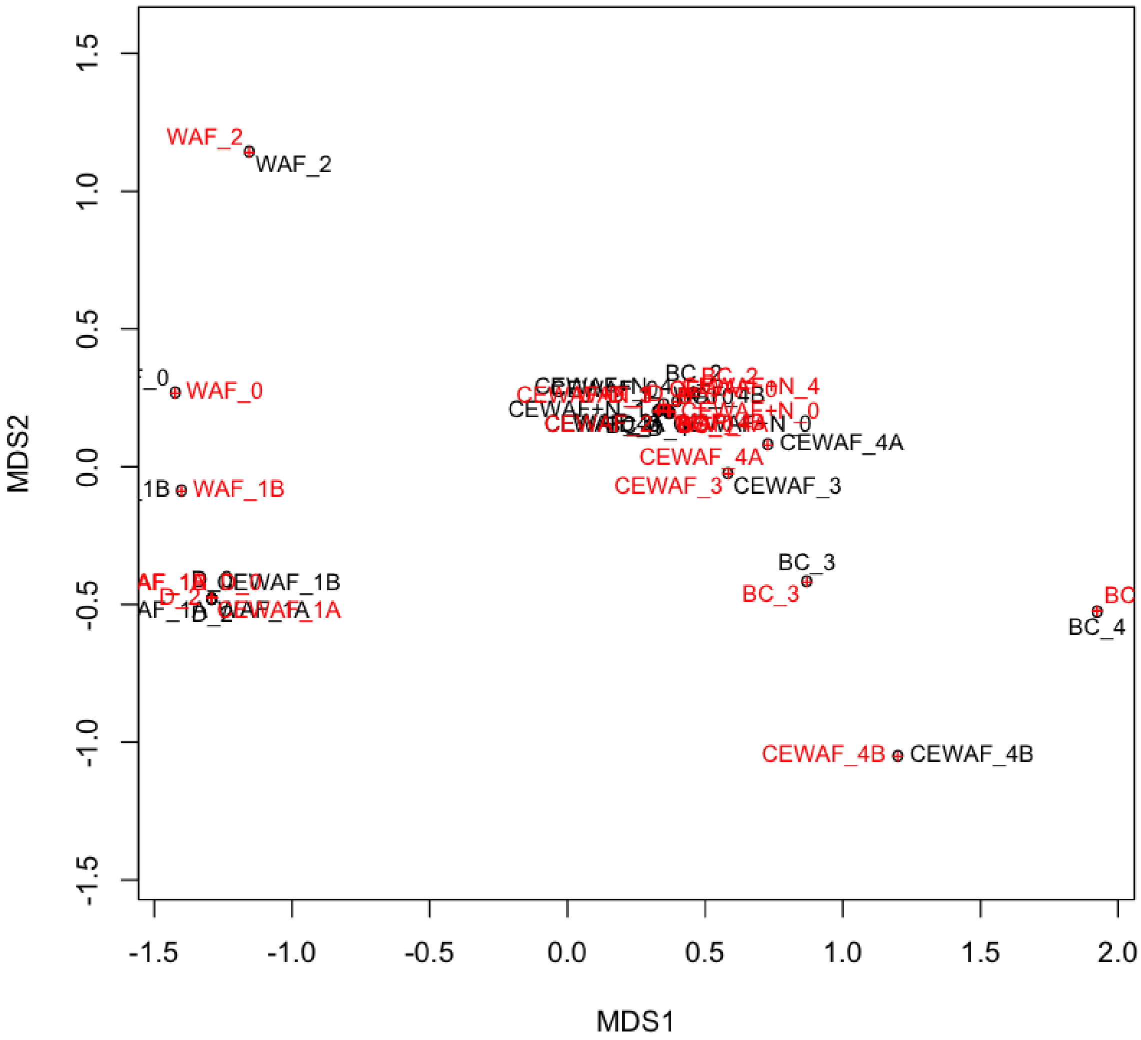

Testing for multivariate differences among groups

We can quantify the relationship between dissimilarity measures and different explanatory variables using the permutational MANOVA (a.k.a. AMOVA) framework in the adonis function in vegan. This method allows ANOVA- like tests of the variance in beta diversity explained by categorical or continuous variables.

Let's quantify the degree to which habitat can explain taxonomic, phylogenetic, and trait dissimilarity among grasslands.

In [24]:

```
# Taxonomic (Bray-Curtis) dissimilarity explained
adonis(comm.bc.dist ~ Dispersant + Oil + Dispersant*Oil + Dispersant*Oil
*Nutrients + Time + Dispersant*Time + Oil*Time + Dispersant*Oil*Time + D
ispersant*Oil*Nutrients*Time, data = metadata)
```

Call:
adonis(formula = comm.bc.dist ~ Dispersant + Oil + Dispersant * Oil + Dispersant * Oil * Nutrients + Time + Dispersant * Time + Oil * Time + Dispersant * Oil * Time + Dispersant * Oil * Nutrients * Time, data = metadata)

Permutation: free
Number of permutations: 999

Terms added sequentially (first to last)

	Df	SumsOfSqs	MeanSqs	F.Model	R2	Pr(>F)	
Dispersant	1	1.0459	1.04588	14.3833	0.26960	0.001	***
Oil	1	0.1286	0.12857	1.7681	0.03314	0.151	
Nutrients	1	0.0681	0.06810	0.9366	0.01756	0.421	
Time	1	0.6823	0.68226	9.3827	0.17587	0.001	***
Dispersant:Oil	1	0.1906	0.19056	2.6206	0.04912	0.071	.
Dispersant:Time	1	0.1986	0.19861	2.7314	0.05120	0.054	.
Oil:Time	1	0.1051	0.10505	1.4448	0.02708	0.232	
Nutrients:Time	1	0.0668	0.06679	0.9185	0.01722	0.463	
Dispersant:Oil:Time	1	0.1574	0.15743	2.1650	0.04058	0.098	.
Residuals	17	1.2362	0.07271		0.31864		
Total	26	3.8794			1.00000		

Signif. codes: 0 '***' 0.001 '**' 0.01 '*' 0.05 '.' 0.1 ' ' 1

In [25]:

```
# Phylogenetic MNTD dissimilarity explained
adonis(comm.mntd.dist ~ Dispersant + Oil + Dispersant*Oil + Dispersant*O
il*Nutrients + Time + Dispersant*Time + Oil*Time + Nutrients*Time + + Di
spersant*Oil*Nutrients*Time, data = metadata)
```

Call:
adonis(formula = comm.mntd.dist ~ Dispersant + Oil + Dispersant *
Oil + Dispersant * Oil * Nutrients + Time + Dispersant * Time
+ Oi l * Time + Nutrients * Time + +Dispersant * Oil *
Nutri
ents * Tim e, data = metadata)

Permutation: free
Number of permutations: 999

Terms added sequentially (first to last)

	Df	SumsOfSqs	MeanSqs	F.Model	R2	Pr(>F)
Dispersant	1	-1.2873e-06	-1.2873e-06	19.75	-0.04915	0.056
.						
Oil	1	3.8791e-06	3.8791e-06	-59.51	0.14811	0.993
Nutrients	1	-8.8700e-07	-8.8700e-07	13.61	-0.03387	0.102
Time	1	2.6194e-05	2.6194e-05	-401.85	1.00013	0.997
Dispersant:Oil	1	1.5973e-05	1.5973e-05	-245.04	0.60986	0.996
Dispersant:Time	1	2.1804e-06	2.1804e-06	-33.45	0.08325	0.976
Oil:Time	1	-4.5807e-06	-4.5807e-06	70.27	-0.17489	0.010**
Nutrients:Time	1	1.2567e-06	1.2567e-06	-19.28	0.04798	0.967
Dispersant:Oil:Time	1	-1.5429e-05	-1.5429e-05	236.70	-0.58911	0.005**
Residuals	17	-1.1081e-06	-6.5200e-08		-0.04231	
Total	26	2.6191e-05			1.00000	

Signif. codes: 0 ‘***’ 0.001 ‘**’ 0.01 ‘*’ 0.05 ‘.’ 0.1 ‘ ’ 1

In [27]:

```
# Phylogenetic MPD dissimilarity explained
adonis(comm.mpd.dist ~ Dispersant + Oil + Dispersant*Oil + Dispersant*Oi
l*Nutrients + Time + Dispersant*Time + Oil*Time + Nutrients*Time + + Dis
persant*Oil*Nutrients*Time, data = metadata)
```

Call:
adonis(formula = comm.mpd.dist ~ Dispersant + Oil + Dispersant * Oil + Dispersant * Oil * Nutrients + Time + Dispersant * Time + Oil * Time + Nutrients * Time + +Dispersant * Oil * Nutrients * Time, data = metadata)

Permutation: free
Number of permutations: 999

Terms added sequentially (first to last)

	Df	SumsOfSqs	MeanSqs	F.Model	R2	Pr(>F)	
Dispersant	1	0.09275	0.092750	2.65097	0.09131	0.001	***
Oil	1	0.03847	0.038471	1.09957	0.03787	0.220	
Nutrients	1	0.02248	0.022479	0.64250	0.02213	0.929	
Time	1	0.05728	0.057278	1.63711	0.05639	0.015	*
Dispersant:Oil	1	0.04122	0.041217	1.17805	0.04058	0.121	
Dispersant:Time	1	0.04988	0.049883	1.42575	0.04911	0.037	*
Oil:Time	1	0.04375	0.043754	1.25059	0.04308	0.108	
Nutrients:Time	1	0.02606	0.026058	0.74478	0.02565	0.792	
Dispersant:Oil:Time	1	0.04909	0.049092	1.40316	0.04833	0.041	*
Residuals	17	0.59478	0.034987		0.58555		
Total	26	1.01576			1.00000		

Signif. codes: 0 ‘***’ 0.001 ‘**’ 0.01 ‘*’ 0.05 ‘.’ 0.1 ‘ ’ 1
