# Supplementary material for: Metatranscriptomic response of deep ocean microbial populations to infusions of oil and/or synthetic chemical dispersant
Source: Appl Environ Microbiol. 2024 Jul 23;90(8):e01083-24. doi: 10.1128/aem.01083-24 (PMC11337851; doi:10.1128/aem.01083-24)
Supplement: Supplemental figures — Figures S1 to S7. [file aem.01083-24-s0005.pdf]

Supplementary Material for:

Metatranscriptomic response of deep ocean microbial populations to infusions of oil and/or synthetic chemical dispersant

Tito D. Peña-Montenegro[a,b,c], Sara Kleindienst[a,†], Andrew E. Allen[d,e], A. Murat Eren[f,g], John P. McCrow[d], Juan David Sánchez-Calderón[h], Jonathan Arnold[b,i], Samantha B. Joye[a,#]

[#]Address correspondence to Samantha B. Joye, mjoye@uga.edu; Tel: 001-706-542-5893; Fax: 001-706-542-5888

This PDF includes:

Figure S1
Figure S2
Figure S3
Figure S4
Figure S5
Figure S6
Figure S7

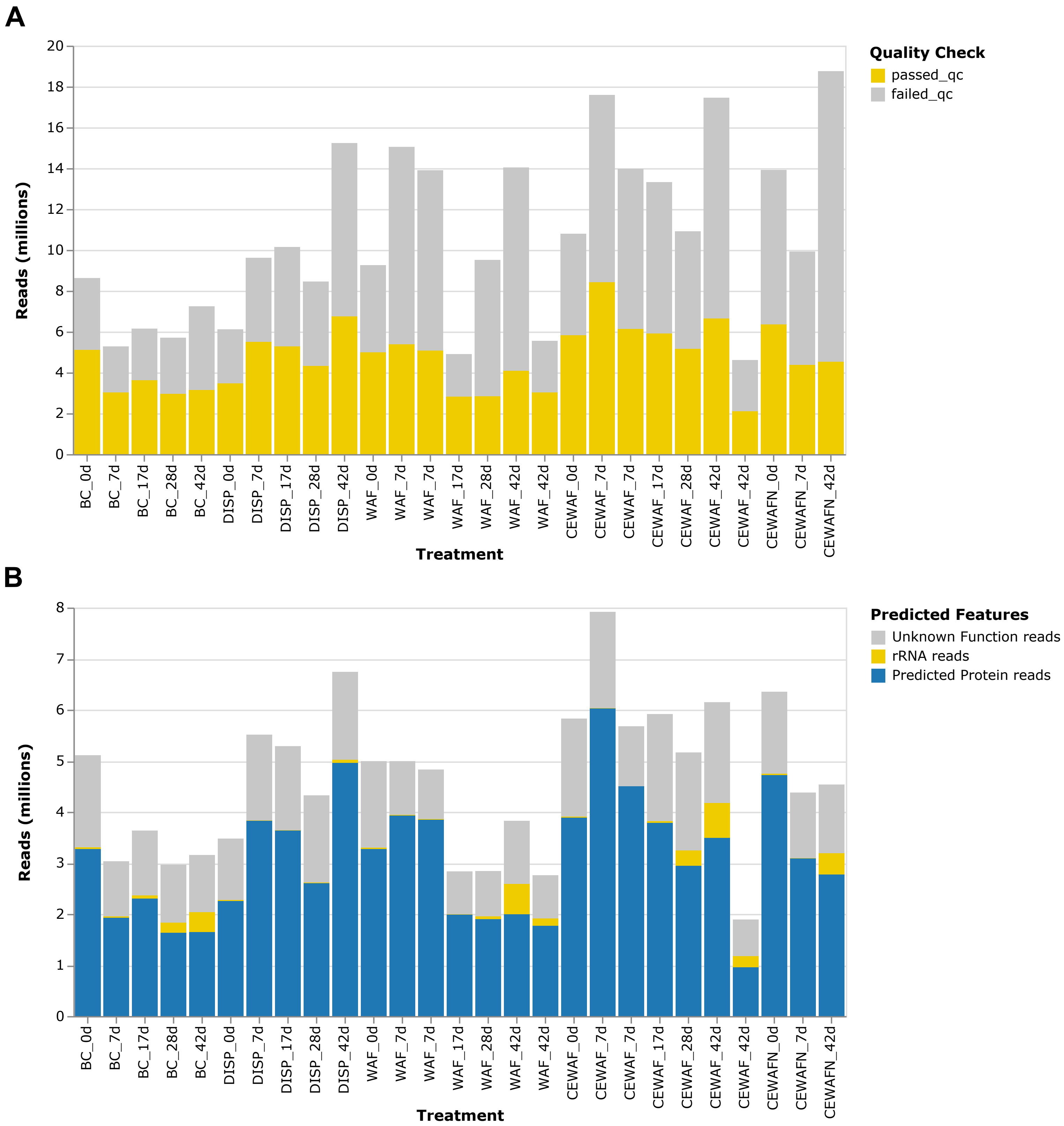
A
Reads (millions)
Quality Check
passed_qc
failed_qc
BC_0d
BC_7d
BC_17d
BC_28d
BC_42d
DISP_0d
DISP_7d
DISP_17d
DISP_28d
DISP_42d
WAF_0d
WAF_7d
WAF_7d
WAF_17d
WAF_28d
WAF_42d
WAF_42d
CEWAF_0d
CEWAF_7d
CEWAF_7d
CEWAF_17d
CEWAF_28d
CEWAF_42d
CEWAF_42d
CEWAFN_0d
CEWAFN_7d
CEWAFN_42d
Treatment
B
Reads (millions)
Predicted Features
Unknown Function reads
rRNA reads
Predicted Protein reads
Treatment

FIG S1. Total reads per sequencing library after quality filter (A) and after annotation (B).

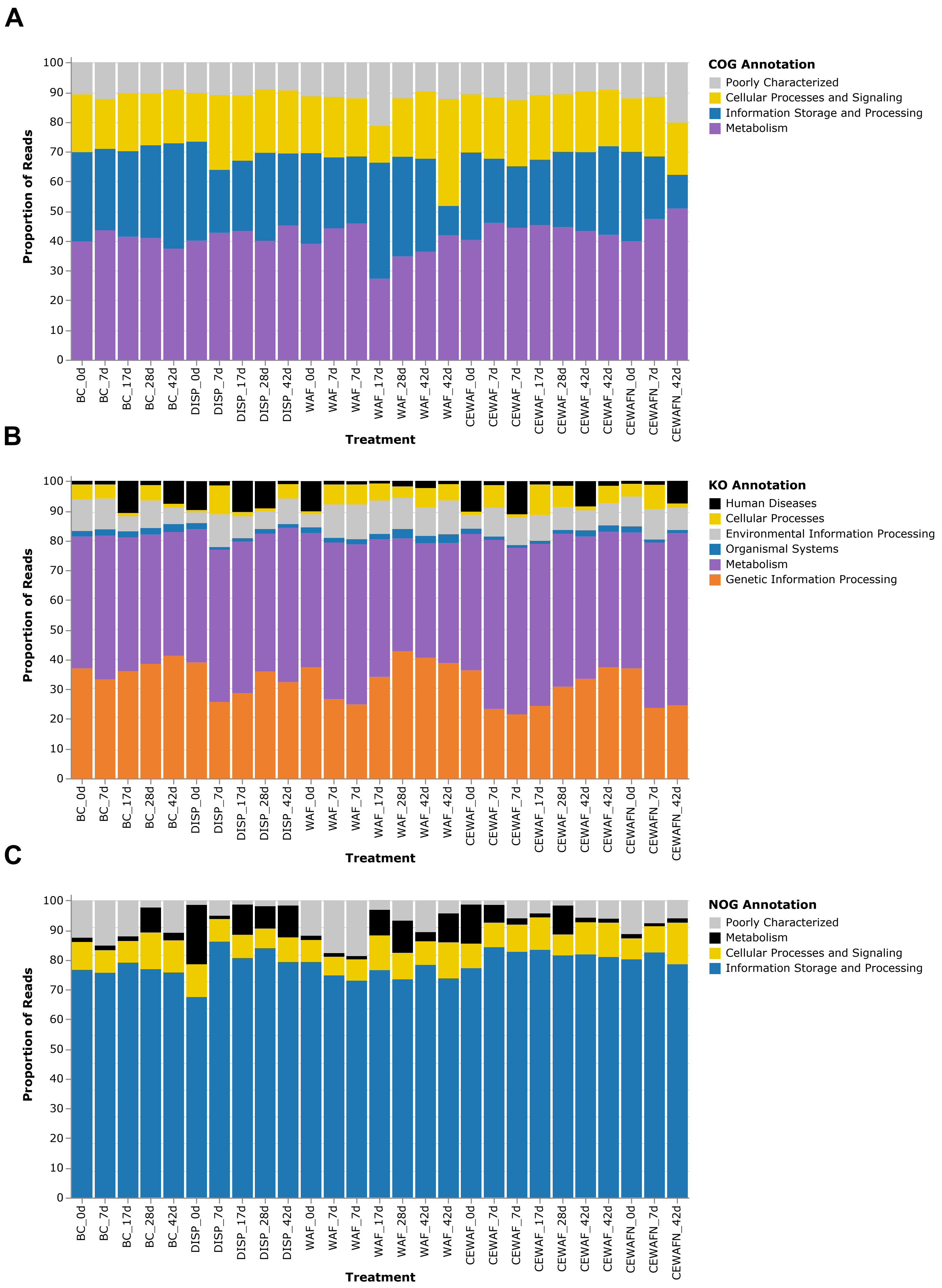
A
Proportion of Reads
Treatment
COG Annotation
Poorly Characterized
Cellular Processes and Signaling
Information Storage and Processing
Metabolism
B
Proportion of Reads
Treatment
KO Annotation
Human Diseases
Cellular Processes
Environmental Information Processing
Organismal Systems
Metabolism
Genetic Information Processing
C
Proportion of Reads
Treatment
NOG Annotation
Poorly Characterized
Metabolism
Cellular Processes and Signaling
Information Storage and Processing
BC_0d
BC_7d
BC_17d
BC_28d
BC_42d
DISP_0d
DISP_7d
DISP_17d
DISP_28d
DISP_42d
WAF_0d
WAF_7d
WAF_7d
WAF_17d
WAF_28d
WAF_42d
WAF_42d
CEWAF_0d
CEWAF_7d
CEWAF_7d
CEWAF_17d
CEWAF_28d
CEWAF_42d
CEWAF_42d
CEWAFN_0d
CEWAFN_7d
CEWAFN_42d

FIG S2. Relative sequence abundance assigned to functional categories using (A) COG, (B) KEGG Orthology, (C) and eggNOG database as references. On average, ‘Metabolism’ was the functional category with the largest fraction of mapped reads from the COG (41.8%) and KEGG (48.1%) annotations; while the ‘Information, Storage and Processing’ category was the top hit mapping category with the eggNOG database (78.6%). Further analysis on a functional or taxonomic level, we used normalized mRNA read counts assigned to known functions or taxa, respectively. The distribution of unique annotation features across the database sources was NR-Blast, 18,869; UNIPROT, 16,547; KO, 2,963; GO, 5,387; COG, 6,468; and eggNOG 3,822 (**Supplementary Data 1**).

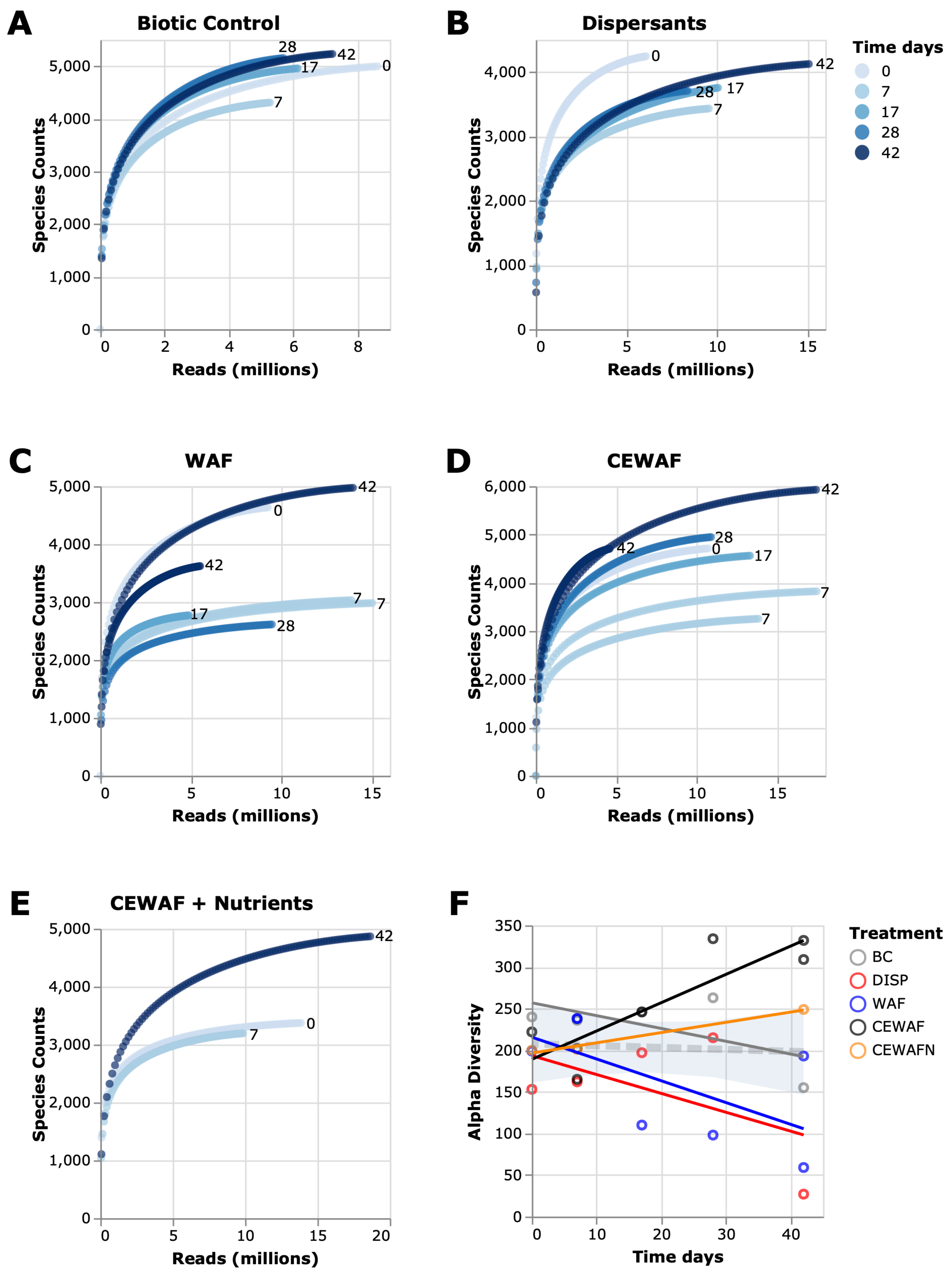
A
Biotic Control
B
Dispersants
Time days
0
7
17
28
42
C
WAF
D
CEWAF
E
CEWAF + Nutrients
F
Treatment
BC
DISP
WAF
CEWAF
CEWAFN
Species Counts
Reads (millions)
Alpha Diversity
Time days

FIG S3. (A-E) Rarefaction analysis showing the distribution of the total distinct species annotations as a function of the number of sequences sampled for each of the libraries in the metatranscriptomic Kleindienst *et al.* data set. (F). Alpha diversity trends across time and treatments of the Kleindienst *et al.* data set.

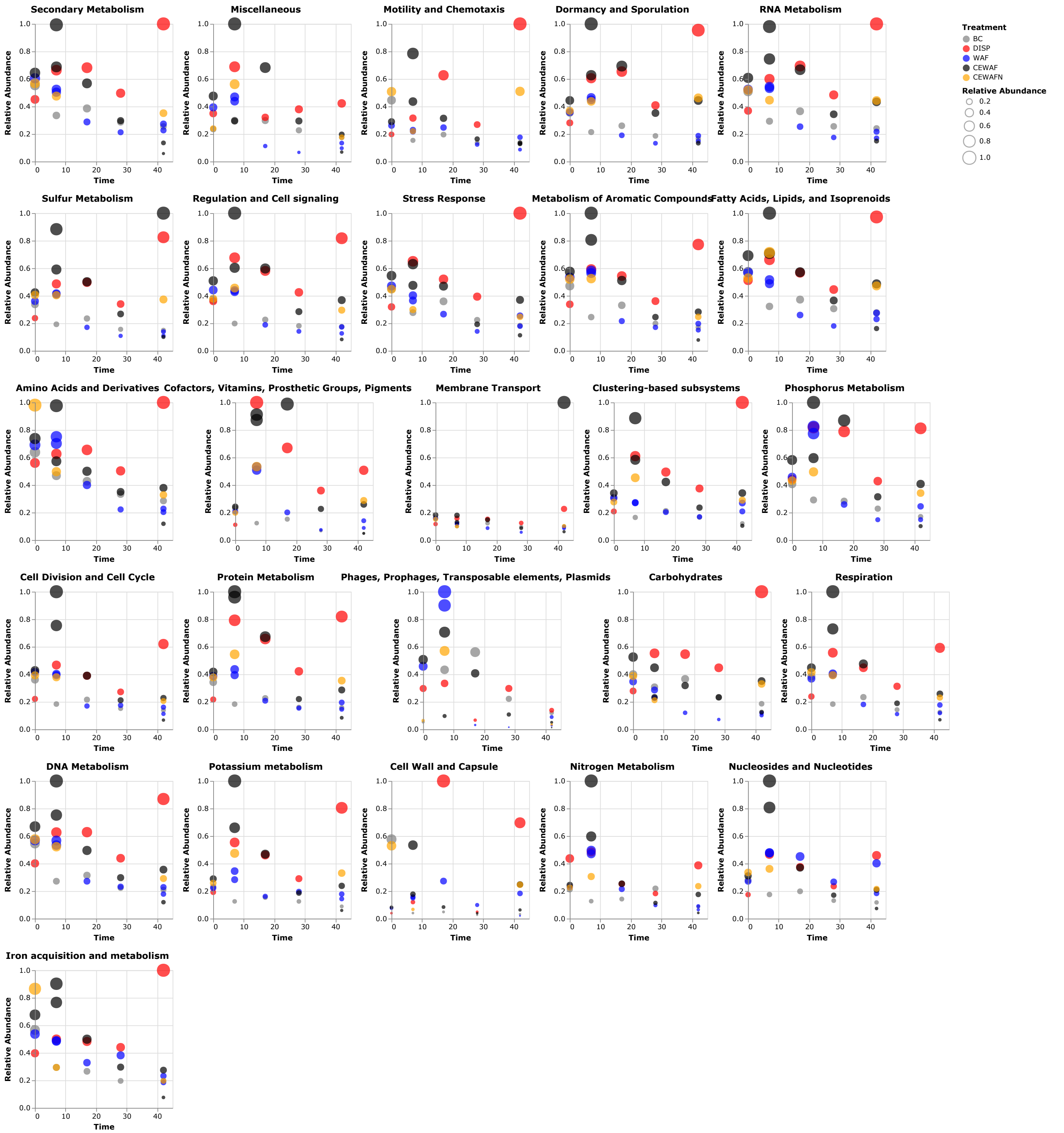

Secondary Metabolism
Miscellaneous
Motility and Chemotaxis
Dormancy and Sporulation
RNA Metabolism
Sulfur Metabolism
Regulation and Cell signaling
Stress Response
Metabolism of Aromatic Compounds
Fatty Acids, Lipids, and Isoprenoids
Amino Acids and Derivatives
Cofactors, Vitamins, Prosthetic Groups, Pigments
Membrane Transport
Clustering-based subsystems
Phosphorus Metabolism
Cell Division and Cell Cycle
Protein Metabolism
Phages, Prophages, Transposable elements, Plasmids
Carbohydrates
Respiration
DNA Metabolism
Potassium metabolism
Cell Wall and Capsule
Nitrogen Metabolism
Nucleosides and Nucleotides
Iron acquisition and metabolism
Relative Abundance
Time
Treatment
BC
DISP
WAF
CEWAF
CEWAFN
Relative Abundance
0.2
0.4
0.6
0.8
1.0

FIG S4. Relative gene expression based on automated SEED subsystems in MG-RAST for the Kleindienst et al. metatranscriptomic data set. Profiles are normalized with respect to the largest expression peak at a given functional category across all treatments. Observations are color coded by treatment: Biotic control (BC), Dispersants (DISP), Oil (WAF), CEWAF, and CEWAFN. Dot sizes are proportional to the relative abundance score.

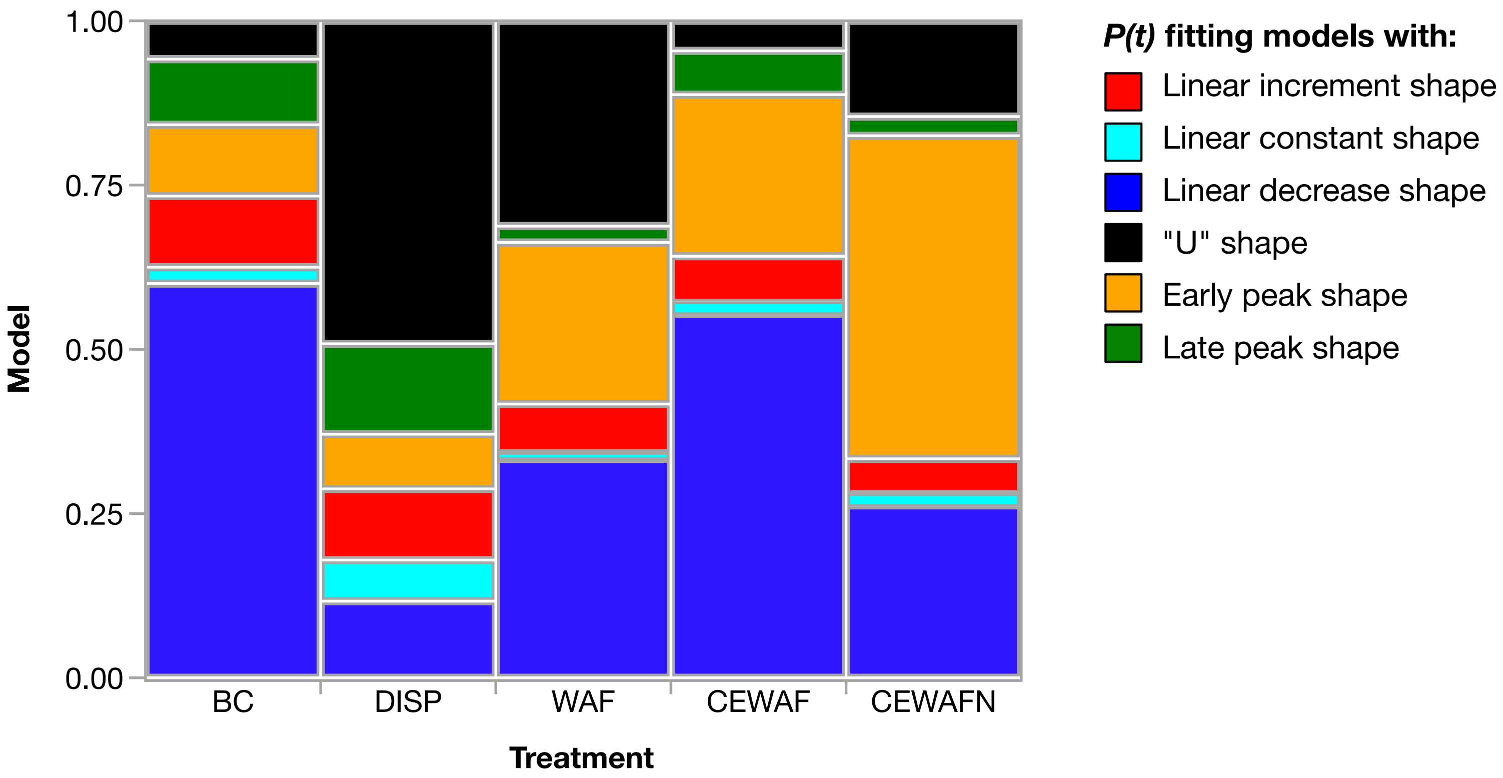

1.00
0.75
0.50
0.25
0.00
Model
BC
DISP
WAF
CEWAF
CEWAFN
Treatment
P(t) fitting models with:
Linear increment shape
Linear constant shape
Linear decrease shape
"U" shape
Early peak shape
Late peak shape

FIG S5. Proportion of time dependent expression *P(t)* fitting models across the experimental treatments in the Kleindienst *et al.* metatranscriptomic data set. Color code matches the distribution of fitting models shown in **Fig. S6**.

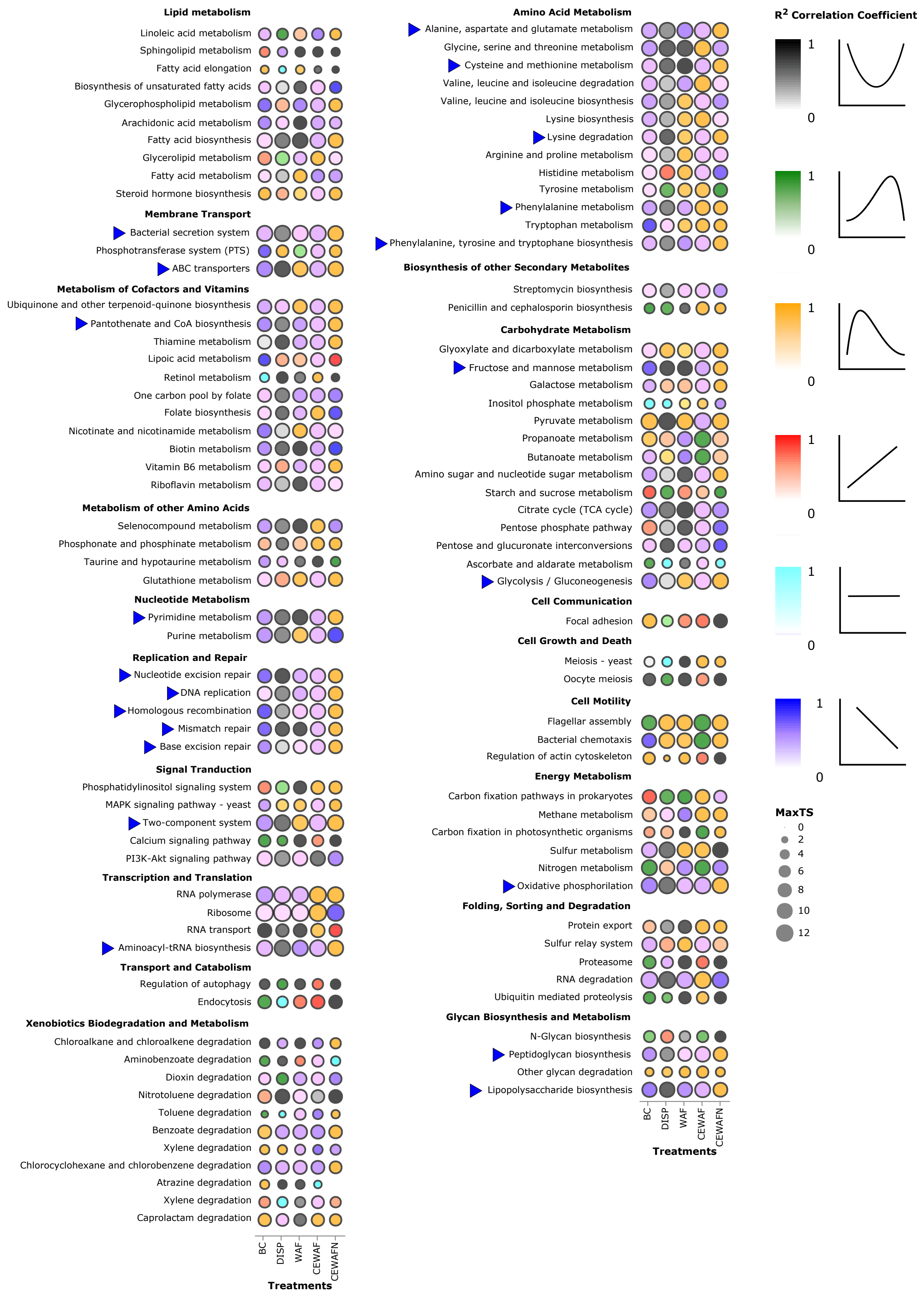
Lipid metabolism
Linoleic acid metabolism
Sphingolipid metabolism
Fatty acid elongation
Biosynthesis of unsaturated fatty acids
Glycerophospholipid metabolism
Arachidonic acid metabolism
Fatty acid biosynthesis
Glycerolipid metabolism
Fatty acid metabolism
Steroid hormone biosynthesis
Membrane Transport
Bacterial secretion system
Phosphotransferase system (PTS)
ABC transporters
Metabolism of Cofactors and Vitamins
Ubiquinone and other terpenoid-quinone biosynthesis
Pantothenate and CoA biosynthesis
Thiamine metabolism
Lipoic acid metabolism
Retinol metabolism
One carbon pool by folate
Folate biosynthesis
Nicotinate and nicotinamide metabolism
Biotin metabolism
Vitamin B6 metabolism
Riboflavin metabolism
Metabolism of other Amino Acids
Selenocompound metabolism
Phosphonate and phosphinate metabolism
Taurine and hypotaurine metabolism
Glutathione metabolism
Nucleotide Metabolism
Pyrimidine metabolism
Purine metabolism
Replication and Repair
Nucleotide excision repair
DNA replication
Homologous recombination
Mismatch repair
Base excision repair
Signal Tranduction
Phosphatidylinositol signaling system
MAPK signaling pathway - yeast
Two-component system
Calcium signaling pathway
PI3K-Akt signaling pathway
Transcription and Translation
RNA polymerase
Ribosome
RNA transport
Aminoacyl-tRNA biosynthesis
Transport and Catabolism
Regulation of autophagy
Endocytosis
Xenobiotics Biodegradation and Metabolism
Chloroalkane and chloroalkene degradation
Aminobenzoate degradation
Dioxin degradation
Nitrotoluene degradation
Toluene degradation
Benzoate degradation
Xylene degradation
Chlorocyclohexane and chlorobenzene degradation
Atrazine degradation
Xylene degradation
Caprolactam degradation
BC
DISP
WAF
CEWAF
CEWAFN
Treatments
Amino Acid Metabolism
Alanine, aspartate and glutamate metabolism
Glycine, serine and threonine metabolism
Cysteine and methionine metabolism
Valine, leucine and isoleucine degradation
Valine, leucine and isoleucine biosynthesis
Lysine biosynthesis
Lysine degradation
Arginine and proline metabolism
Histidine metabolism
Tyrosine metabolism
Phenylalanine metabolism
Tryptophan metabolism
Phenylalanine, tyrosine and tryptophane biosynthesis
Biosynthesis of other Secondary Metabolites
Streptomycin biosynthesis
Penicillin and cephalosporin biosynthesis
Carbohydrate Metabolism
Glyoxylate and dicarboxylate metabolism
Fructose and mannose metabolism
Galactose metabolism
Inositol phosphate metabolism
Pyruvate metabolism
Propanoate metabolism
Butanoate metabolism
Amino sugar and nucleotide sugar metabolism
Starch and sucrose metabolism
Citrate cycle (TCA cycle)
Pentose phosphate pathway
Pentose and glucuronate interconversions
Ascorbate and aldarate metabolism
Glycolysis / Gluconeogenesis
Cell Communication
Focal adhesion
Cell Growth and Death
Meiosis - yeast
Oocyte meiosis
Cell Motility
Flagellar assembly
Bacterial chemotaxis
Regulation of actin cytoskeleton
Energy Metabolism
Carbon fixation pathways in prokaryotes
Methane metabolism
Carbon fixation in photosynthetic organisms
Sulfur metabolism
Nitrogen metabolism
Oxidative phosphorilation
Folding, Sorting and Degradation
Protein export
Sulfur relay system
Proteasome
RNA degradation
Ubiquitin mediated proteolysis
Glycan Biosynthesis and Metabolism
N-Glycan biosynthesis
Peptidoglycan biosynthesis
Other glycan degradation
Lipopolysaccharide biosynthesis
BC
DISP
WAF
CEWAF
CEWAFN
Treatments
R2 Correlation Coefficient
1
0
1
0
1
0
1
0
1
0
1
0
MaxTS
0
2
4
6
8
10
12

FIG S6. Fitting models for each pathway and treatment aiming for the greatest correlation coefficient R^2. Dot sizes are proportional to largest normalized transcriptomic signal (MaxTS) at a given pathway and treatment. Color scale is matching the assigned best fitting model among the tested models from top to bottom: U-shaped (U) second order linear model in black, negatively skewed log-normal model to shape a late peak in green, positively skewed log-normal model to shape an early peak (EP) in golden, first order increasing linear model (slope>1.88) in red, first order constant linear model (|slope|≤1.88) in cyan, and first order decreasing linear (DL) model (slope<-1.88) in blue. Blue triangles indicate those pathways that followed a DL trend in the biotic control, a U trend in the dispersant-only treatment, and an EP trend in the CEWAFN treatment.

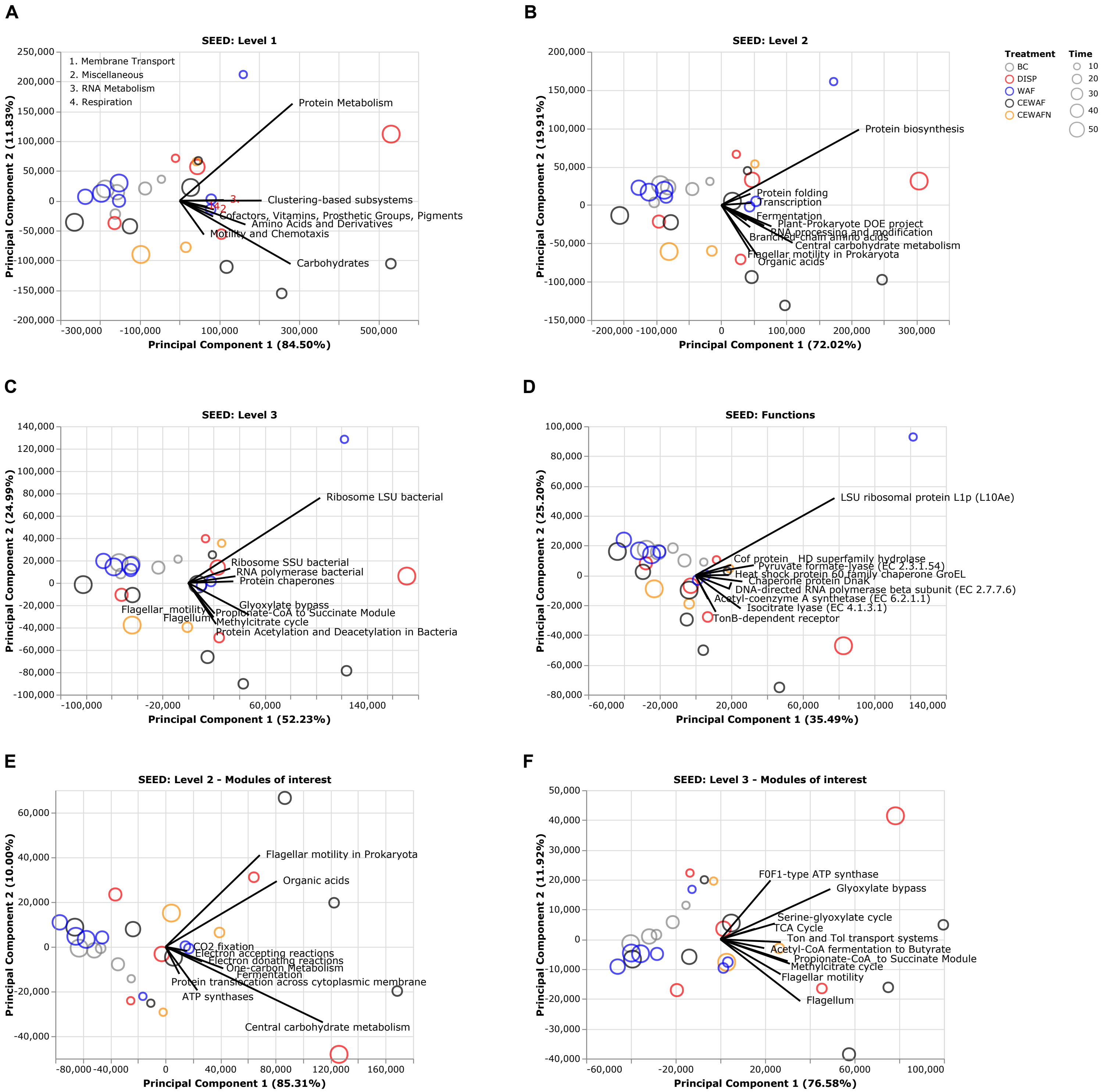

A
SEED: Level 1
1. Membrane Transport
2. Miscellaneous
3. RNA Metabolism
4. Respiration
Protein Metabolism
Clustering-based subsystems
Cofactors, Vitamins, Prosthetic Groups, Pigments
Amino Acids and Derivatives
Motility and Chemotaxis
Carbohydrates
Principal Component 2 (11.83%)
Principal Component 1 (84.50%)
B
SEED: Level 2
Protein biosynthesis
Protein folding
Transcription
Fermentation
Plant-Prokaryote DOE project
RNA processing and modification
Branched-chain amino acids
Central carbohydrate metabolism
Flagellar motility in Prokaryota
Organic acids
Principal Component 2 (19.91%)
Principal Component 1 (72.02%)
Treatment
BC
DISP
WAF
CEWAF
CEWAFN
Time
10
20
30
40
50
C
SEED: Level 3
Ribosome LSU bacterial
Ribosome SSU bacterial
RNA polymerase bacterial
Protein chaperones
Glyoxylate bypass
Flagellar_motility
Flagellum
Propionate-CoA to Succinate Module
Methylcitrate cycle
Protein Acetylation and Deacetylation in Bacteria
Principal Component 2 (24.99%)
Principal Component 1 (52.23%)
D
SEED: Functions
LSU ribosomal protein L1p (L10Ae)
Cof protein, HD superfamily hydrolase
Pyruvate formate-lyase (EC 2.3.1.54)
Heat shock protein 60 family chaperone GroEL
Chaperone protein DnaK
DNA-directed RNA polymerase beta subunit (EC 2.7.7.6)
Acetyl-coenzyme A synthetase (EC 6.2.1.1)
Isocitrate lyase (EC 4.1.3.1)
TonB-dependent receptor
Principal Component 2 (25.20%)
Principal Component 1 (35.49%)
E
SEED: Level 2 - Modules of interest
Flagellar motility in Prokaryota
Organic acids
CO2 fixation
Electron accepting reactions
Electron donating reactions
One-carbon Metabolism
Fermentation
Protein translocation across cytoplasmic membrane
ATP synthases
Central carbohydrate metabolism
Principal Component 2 (10.00%)
Principal Component 1 (85.31%)
F
SEED: Level 3 - Modules of interest
F0F1-type ATP synthase
Glyoxylate bypass
Serine-glyoxylate cycle
TCA Cycle
Ton and Tol transport systems
Acetyl-CoA fermentation to Butyrate
Propionate-CoA to Succinate Module
Methylcitrate cycle
Flagellar motility
Flagellum
Principal Component 2 (11.92%)
Principal Component 1 (76.58%)

FIG S7. Principal component analysis (PCA) of functional features abundance for the Kleindienst *et al.* data set across SEED annotation levels 1 (A), 2 (B), 3 (C), and SEED functions (D). PCA focused on the functional categories Motility and Chemotaxis, Carbohydrates, Membrane Transport and Respiration are shown for levels 2 (E), and 3 (F). Solid lines represent the top ten loading vectors explaining the variation of expressed genes in the analysis.
